# Hepatitis C virus NS3-4A protease regulates the lipid environment for RNA replication by cleaving host enzyme 24-dehydrocholesterol reductase

**DOI:** 10.1074/jbc.RA120.013455

**Published:** 2020-07-08

**Authors:** Lorillee Tallorin, Valerie A. Villareal, Chih-Yun Hsia, Mary A. Rodgers, Dominique J. Burri, Marc-Philipp Pfeil, Paula Montero Llopis, Brett D. Lindenbach, Priscilla L. Yang

**Affiliations:** 1 Department of Microbiology and Blavatnik Institute, Harvard Medical School, Boston, Massachusetts, USA; 2 Department of Microbial Pathogenesis, Yale Medical School, New Haven, Connecticut, USA

**Keywords:** hepatitis C virus (HCV), RNA replication, lipid environment, membrane remodeling, host–pathogen interaction, viral replication, post-translational modification (PTM), viral protease, membrane lipid, cholesterol metabolism, desmosterol

## Abstract

Many RNA viruses create specialized membranes for genome replication by manipulating host lipid metabolism and trafficking, but in most cases, we do not know the molecular mechanisms responsible or how specific lipids may impact the associated membrane and viral process. For example, hepatitis C virus (HCV) causes a specific, large-fold increase in the steady-state abundance of intracellular desmosterol, an immediate precursor of cholesterol, resulting in increased fluidity of the membrane where HCV RNA replication occurs. Here, we establish the mechanism responsible for HCV's effect on intracellular desmosterol, whereby the HCV NS3-4A protease controls activity of 24-dehydrocholesterol reductase (DHCR24), the enzyme that catalyzes conversion of desmosterol to cholesterol. Our cumulative evidence for the proposed mechanism includes immunofluorescence microscopy experiments showing co-occurrence of DHCR24 and HCV NS3-4A protease; formation of an additional, faster-migrating DHCR24 species (DHCR24*) in cells harboring a HCV subgenomic replicon RNA or ectopically expressing NS3-4A; and biochemical evidence that NS3-4A cleaves DHCR24 to produce DHCR24* *in vitro* and *in vivo*. We further demonstrate that NS3-4A cleaves DHCR24 between residues Cys^91^ and Thr^92^ and show that this reduces the intracellular conversion of desmosterol to cholesterol. Together, these studies demonstrate that NS3-4A directly cleaves DHCR24 and that this results in the enrichment of desmosterol in the membranes where NS3-4A and DHCR24 co-occur. Overall, this suggests a model in which HCV directly regulates the lipid environment for RNA replication through direct effects on the host lipid metabolism.

Viruses are well-known for their hijacking of cellular processes to enable their own replication while also evading or counteracting the host response to infection (1). This is accomplished using diverse mechanisms that include viral regulation of host transcription and translation, as well as post-translational regulation of host factors (2, 3). For example, hepatitis C virus (HCV) disrupts sensing by the host innate immune system by directly cleaving two critical adaptor proteins, mitochondrial antiviral signaling protein (MAVS) and Toll–interleukin-1 receptor domain containing adaptor-inducing interferon-β (TRIF) (4–6) HCV. Other viruses are also known to alter host metabolism to meet the energetic and metabolic needs of replication, for example, by altering the expression and/or localization of host proteins responsible for synthesis or trafficking of critical metabolites. One such example is the interaction of HCV nonstructural protein 5A (NS5A) with phosphatidylinositol 4-kinase α, which appears to regulate the phosphorylation status and function of NS5A while also stimulating the accumulation of phosphatidylinositol 4-phosphates that recruit viral and host factors required for RNA replication (7, 8). The clinical association of HCV infection with steatosis and hyperlipidemia positions it as a good experimental system for studying how viruses perturb and exploit the host lipid machinery (9–13).

Several studies of chronic HCV patients have confirmed that the presence of HCV affects host sterol metabolism (9–13). Both proteomic and lipidomic profiling by targeted liquid chromatography - mass spectrometry (LC-MS) have shown that HCV perturbs multiple host lipid biosynthetic pathways (14). Additionally, unbiased lipidomic MS profiling by our laboratory previously discovered that HCV causes a 10-fold increase in intracellular desmosterol, an immediate precursor to cholesterol, without affecting the abundance of cholesterol (15–17). We demonstrated that the HCV-induced accumulation of desmosterol is functionally important for HCV replication, as evidenced by strong reduction of accumulated HCV RNA when desmosterol is depleted from cells and restoration of HCV RNA levels upon addition of exogenous desmosterol but not cholesterol (15, 17). However, how HCV regulates host metabolism to cause an increase in the steady-state abundance of desmosterol remains unknown.

Here, we establish a mechanism used by HCV to regulate desmosterol abundance. The mechanism involves post-translational regulation of the key enzyme in desmosterol metabolism, 24-dehydrocholesterol reductase (DHCR24), which converts desmosterol to cholesterol. Our study reveals that DHCR24 is proteolytically cleaved between Cys^91^ and Thr^92^ by HCV NS3-4A protease, resulting in inactivation of DHCR24 and accumulation of desmosterol in the replication membranes where NS3-4A and DHCR24 co-reside. This, in turn, is associated with robust HCV replication. Together, our studies suggest a model in which the HCV NS3-4A protease remodels the host lipid membrane by directly interacting with and cleaving a host biosynthetic enzyme to generate a chemical environment that promotes increased viral RNA replication.

## Results

### HCV reduces the intracellular conversion of desmosterol to cholesterol without affecting the expression or abundance of cholesterol biosynthetic enzymes DHCR7 and DHCR24

**Figure 1.**
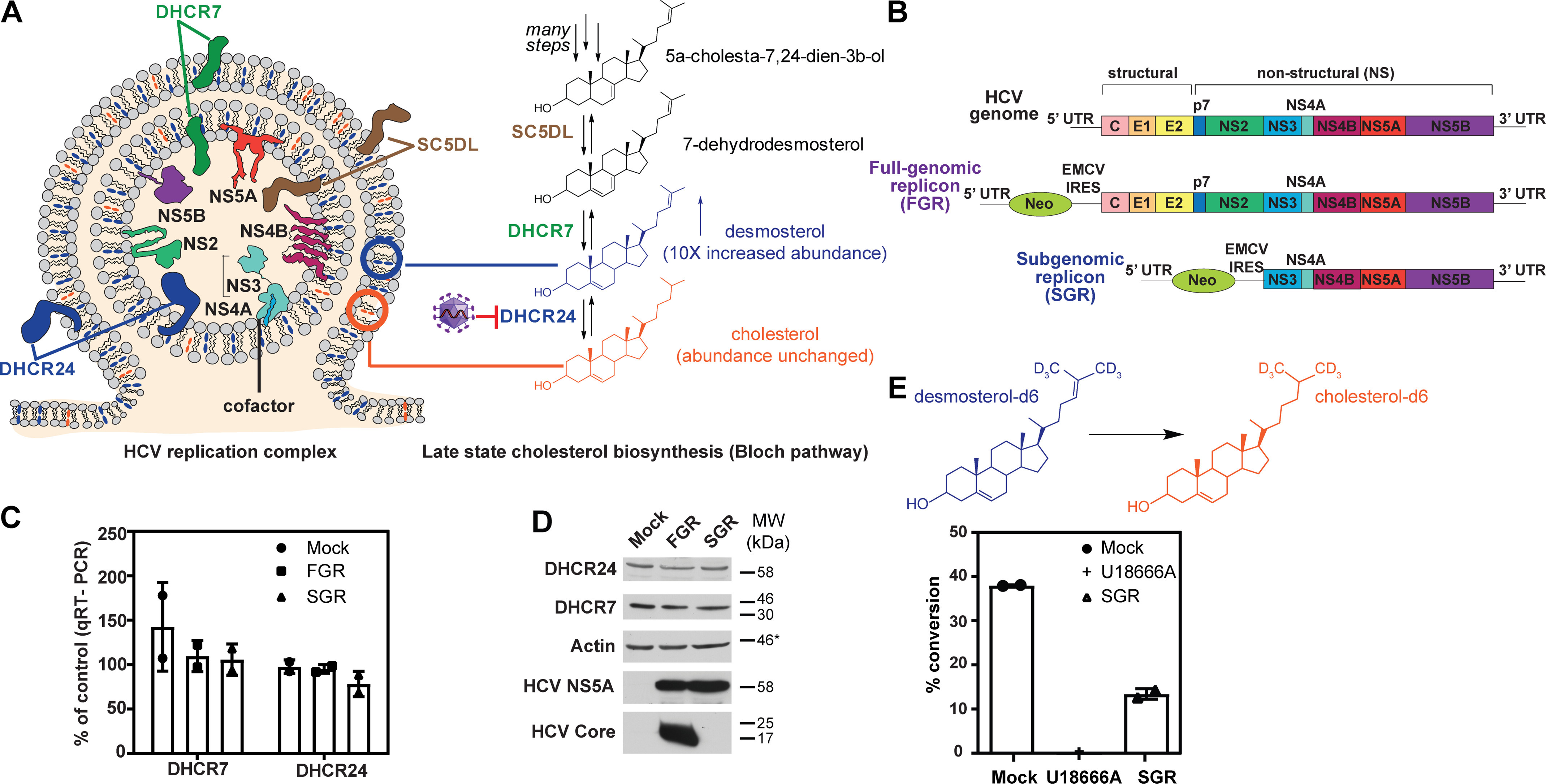
**Effect of HCV on enzymes in the Bloch pathway of late-stage cholesterol biosynthesis.** *A*, the nonstructural (*NS*) proteins of HCV form a replication complex located on a specialized membrane derived from the host ER. Our data indicate that the enzymes that catalyze cholesterol biosynthesis, which are integral membrane proteins found on the ER surface, co-reside with the nonstructural proteins on the replication membrane in addition to being present on ER membrane itself. Desmosterol, the penultimate molecule in the Bloch branch of cholesterol biosynthesis, is depicted as *blue ovals*; cholesterol is depicted as *orange ovals*. *B*, FGR and SGR RNAs derived from HCV clone JFH1 were used as experimental models to examine the effects of HCV on desmosterol synthesis and metabolism. Replicons were stably expressed in Huh7.5 cells. *C*, FGR and SGR RNAs do not significantly alter mRNA abundance of DHCR7 or DHCR24, as measured by RT-qPCR. The *bar graph* represents the average of two biological replicates with *error bars* equal to the standard deviation. Neither FGR nor SGR caused a significant change in the abundance of DHCR7 or DHCR24 transcripts. *D*, the SGR RNA does not cause a significant change in the steady-state abundance of DHCR7 or DHCR24 proteins as assessed by immunoblot of whole-cell lysates. *, The molecular mass (*MW*) marker for the immunoblot probed for actin was excised during processing; however, the actin band relative to core and NS5A was consistent between this and other immunoblots in which the molecular mass markers were recorded. The location of the 46-kDa marker relative to actin on comparable immunoblots is indicated. *E*, cellular conversion of deuterated desmosterol (desmosterol-d6) to deuterated cholesterol (cholesterol-d6) was monitored by extraction of cellular lipids and quantification of desmosterol-d6 and cholesterol-d6 by LC-MS. No conversion of desmosterol-d6 to cholesterol-d6 was observed in cells treated with U18666A, a DHCR24 inhibitor, which was used as a positive control. The presence of the SGR RNA causes a significant decrease in conversion of desmosterol-d6 to cholesterol-d6 (Student's *t* test *p* value of 0.0012 when compared with Huh7.5-negative control cells, “Mock”). The *bar graph* represents the average of two biological replicates with *error bars* equal to standard deviation.

Desmosterol is produced in the Bloch branch of late-stage cholesterol biosynthesis by reduction of 7-dehydrodesmosterol by 7-dehydrodesmosterol reductase (DHCR7). Under normal physiological conditions, desmosterol does not accumulate and is rapidly converted to cholesterol by DHCR24 (Fig. 1*A*). Because HCV has no known gene products capable of catalyzing lipid synthesis or metabolism, we investigated whether HCV perturbs the expression of DHCR7 and DHCR24. For these experiments, we used autonomously replicating RNAs encoding either the full-length HCV polyprotein (full-length genomic replicon, or FGR) or encoding only the NS3-5B proteins (subgenomic replicon, or SGR) (Fig. 1*B*) (18). These and other replicon systems are well-established models for studying HCV gene expression and RNA replication in the absence of the complicating effects of viral entry, assembly, or egress.

We first quantified DHCR7 and DHCR24 transcripts by reverse-transcription quantitative PCR (RT-qPCR) assay and found that the abundance of these transcripts is unaffected by the presence or absence of the HCV full-genomic and subgenomic replicon RNAs (Fig. 1*C*). This finding is consistent with transcript profiling studies showing that HCV infection induces no changes in DHCR7 or DHCR24 mRNA abundance (19, 20) and indicates that the HCV-associated increase in intracellular desmosterol is not due to viral perturbation at the mRNA level. Next, we examined the steady-state abundance of DHCR7 or DHCR24 proteins by immunoblot analysis of whole cell lysates and detected no differences between cells harboring HCV replicon RNAs and negative control cells (Fig. 1*D*). Although we cannot exclude the possibility that noncoding RNAs might regulate translation of DHCR7 and/or DHCR24 mRNAs, this does not appear to happen to a significant extent based on the steady-state abundance of DHCR7 and DHCR24 proteins. These data show that HCV's effect on desmosterol homeostasis does not involve changes in the expression or abundance of DHCR7 and DHCR24. This led us to consider the possibility that HCV increases intracellular desmosterol by either increasing DHCR7 activity or decreasing DHCR24 activity. Because neither enzyme is known to be rate-determining for the pathway and because desmosterol is rapidly converted to cholesterol under normal physiological conditions, we deemed a virus-associated reduction in the DHCR24-catalyzed conversion of desmosterol to cholesterol to be the more likely scenario.

To investigate this possibility, cells harboring HCV replicon RNAs and negative control cells were treated with medium containing deuterated desmosterol (desmosterol-d6), and conversion of the desmosterol-d6 to cholesterol-d6 was monitored over time by LC-MS analysis of extracted lipidomes. Conversion of desmosterol-d6 to cholesterol-d6 was reduced in cells harboring the HCV subgenomic replicon or treated with U18666A, a small molecule known to inhibit DHCR24's enzymatic activity and intracellular sterol transport (21–24), when compared with cells lacking the replicon (Mock) (Fig. 1*E*). This observation supports the proposal that HCV negatively regulates DHCR24 activity.

### DHCR24 is post-translationally modified in cells expressing the active NS3-4A protease

**Figure 2.**
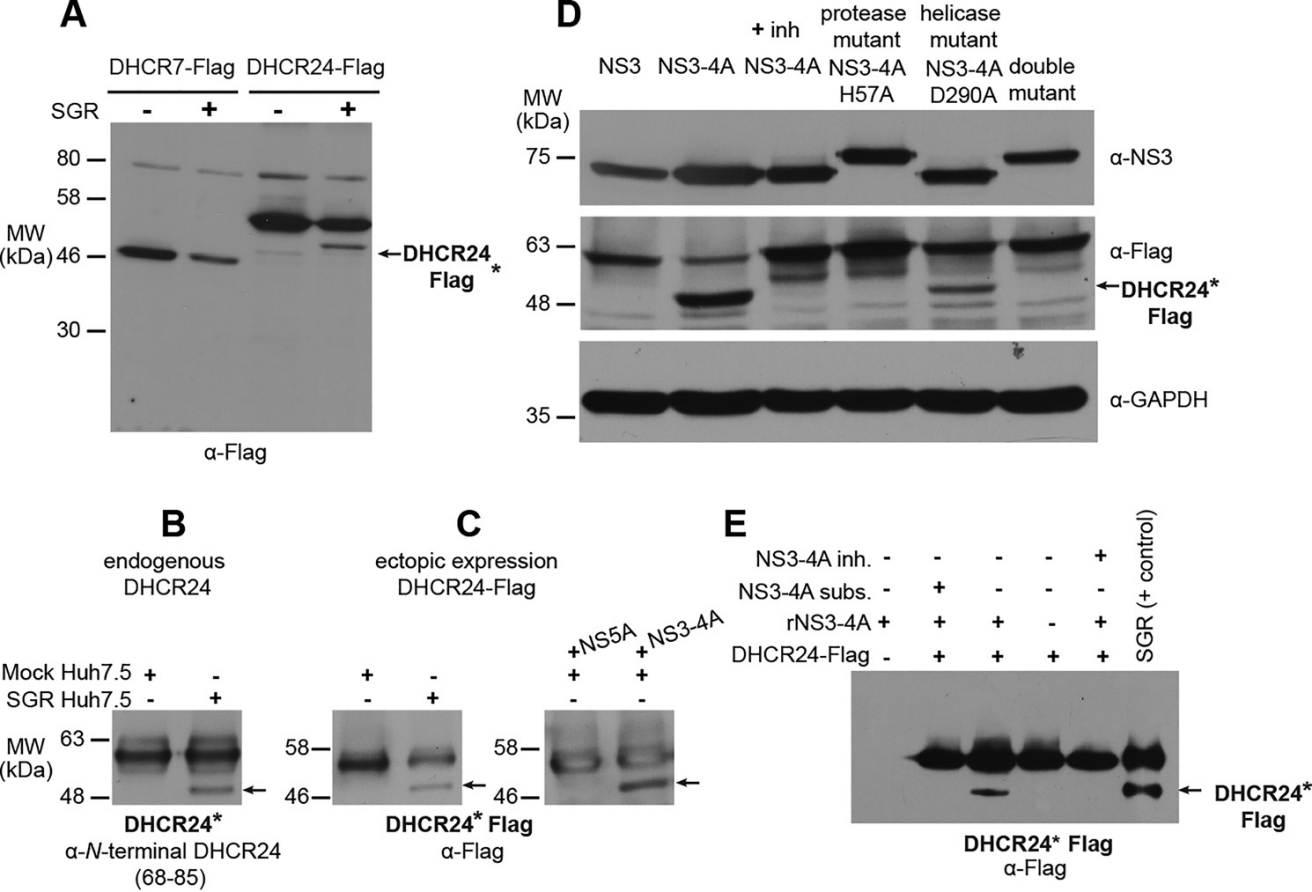
**DHCR24 is post-translationally modified in the presence of NS3-4A.** *A*, FLAG-tagged DHCR7 or DHCR24 was immunoprecipitated and analyzed by immunoblot. A faster-migrating DHCR24-FLAG species, which we dubbed DHCR24*–*FLAG*, is detected in cells stably expressing SGR. DHCR24* is marked in this figure with an *arrow*. *B*, an analogously faster-migrating DHCR24* species was also detected when endogenous DHCR24 was immunoprecipitated from Huh7.5 cells stably expressing SGR but not in samples prepared from negative control Huh7.5 cells. *C*, formation of DHCR24*–FLAG induced in the presence of the SGR RNA (*left blot*) is recapitulated with ectopic expression of NS3-4A but not NS5A (*right blot*). *D*, formation of DHCR24*–FLAG occurs in cells expressing the active NS3-4A protease, but this is blocked in the presence of danoprevir, an NS3-4A inhibitor (*inh*), or when NS3 is expressed alone without NS4A. DHCR24*–FLAG also fails to form when the protease active site is mutated to destroy catalytic function (NS3-4A-H57A), whereas DHCR24*–FLAG formation is not impaired by mutation of the NS3 helicase active site (NS3-4A D290A). *E*, the formation of DHCR24*–FLAG in cells expressing NS3-4A is recapitulated *in vitro* when DHCR24-FLAG immunoprecipitated from Huh7.5 cells is incubated with recombinant NS3-4A. This *in vitro* reaction is blocked in the presence of NS3-4A inhibitor or when a peptide substrate (*subst*) of NS3-4A is added in excess. DHCR24–FLAG immunoprecipitated from Huh7.5 SGR cells is included as a positive control in the *far right lane*. Although molecular mass markers are not indicated for this blot because they were excised during processing, the relative locations of the *bands* are consistent with DHCR24 (*upper band*) and DHCR24* (*lower band*) observed in other experiments. *MW*, molecular mass.

Because HCV has no effect on DHCR7 or DHCR24 expression or abundance yet appears to affect the DHCR24-catalyzed reaction in cells, we next investigated whether DHCR24 is post-translationally modified in the presence of the virus. Immunoblot analysis of cells transfected with a C-terminal FLAG-tagged DHCR7 or DHCR24 (DHCR7–FLAG and DHCR24–FLAG respectively) revealed an additional, faster-migrating DHCR24-FLAG species in the presence of HCV SGR (Fig. 2*A*). We confirmed that formation of this faster-migrating DHCR24 species (DHCR24*) was not an artifact of ectopic expression of DHCR24–FLAG by demonstrating that DHCR24* is detected when endogenous DHCR24 is immunoprecipitated from cells harboring the SGR (Fig. 2*B*).

To map which of the HCV proteins expressed by the subgenomic replicon (NS3, NS4A, NS4B, NS5A, and NS5B) is sufficient to induce formation of DHCR24*, we first co-expressed NS3-4A and NS5A individually with DHCR24–FLAG (Fig. 2*C*). NS3 contains a C-terminal helicase domain and an N-terminal serine protease domain that requires its cofactor NS4A for catalytic activity (25). Although no inherent enzymatic function has been reported for NS5A, it is known to affect host lipids through interaction with phosphatidylinositol 4-kinase α (8) and is hypothesized to have a significant role in coordinating genome replication and viral assembly (26). We found that expression of the NS3-4A protease is sufficient to induce formation of DHCR24*–FLAG. Production of DHCR24*–FLAG requires active protease activity because no DHCR24*–FLAG is produced in the presence of the NS3-4A inhibitor danoprevir or when NS3 is expressed alone without NS4A. Further, production of DHCR24* is lost when a catalytic residue in the protease active site is mutated (NS3-4A–H57A) (28–30) but is unaffected by a point mutation introduced to the NS3 helicase active site (NS3-4A–D290A) (29) (Fig. 2*D*). Together, these results demonstrate that formation of DHCR24* requires the active NS3-4A serine protease domain.

We further validated that the active NS3-4A protease is sufficient to generate DHCR24* by immunoprecipitating DHCR24–FLAG from uninfected cells and incubating this protein with recombinant NS3-4A. This successfully recapitulated formation of DHCR24*–FLAG *in vitro* (Fig. 2*E*). This *in vitro* reaction is blocked in the presence of telaprevir, a Food and Drug Administration–approved NS3-4A inhibitor, or a competitive peptide mimetic (31, 32) (Fig. 2*E*). Taken together, these experiments demonstrate that the NS3-4A protease causes production of DHCR24*.

### DHCR24 is a direct substrate of the NS3-4A protease

To examine the possibility that NS3-4A directly cleaves DHCR24, we isolated the two DHCR24 species observed by SDS-PAGE following incubation of immunoprecipitated DHCR24–FLAG with recombinant NS3-4A protease *in vitro*. “Bottom-up” LC-MS/MS analysis of the isolated species demonstrated that both bear the FLAG-derived DYKDDDDK sequence and thus are derived from DHCR24–FLAG (Fig. S1). Likewise, LC-MS/MS analysis of endogenous DHCR24 immunoprecipitated from cells harboring the HCV subgenomic replicon verified that both products are derived from DHCR24 (Fig. 2*B*). These results indicate that DHCR24 is a substrate for NS3-4A–mediated proteolysis. Due to several technical issues, including difficulty in driving complete conversion of immunoprecipitated DHCR24 to DHCR24*, poor ionization of the reaction products, and limited recovery of recombinant DHCR24 proteins due to high hydrophobicity, we were unable to identify the position of the cleavage site by LC-MS/MS.

### Mapping of cleavage of DHCR24 by NS3-4A

**Figure 3.**
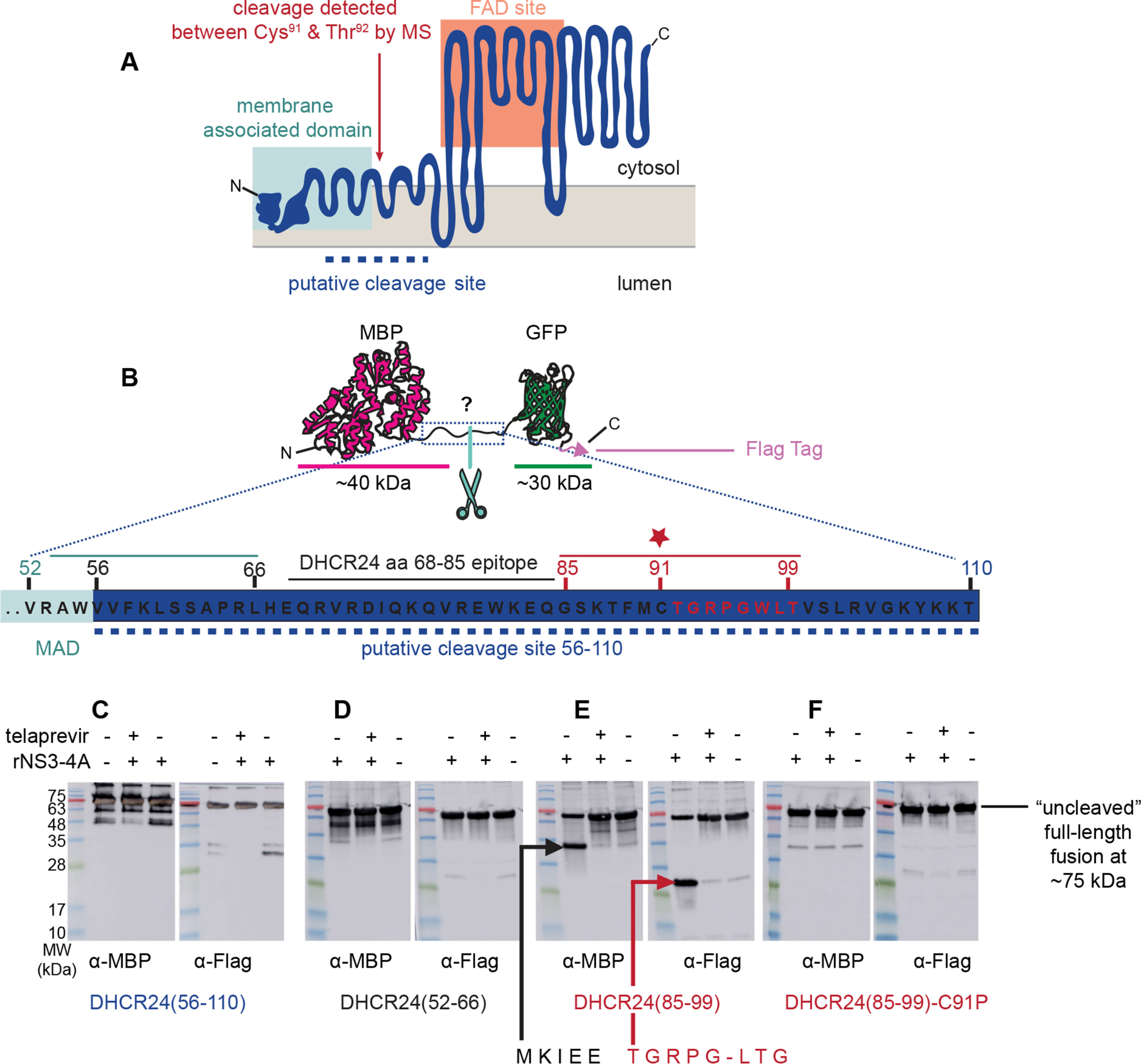
**DHCR24 is proteolytically cleaved by NS3-4A between Cys^91^ and Thr^92^.** *A*, the topology model of DHCR24 predicts (54) an N-terminal membrane-associated domain that tethers a C-terminal FAD-binding site to the surface of the ER membrane. The dashed line indicates the region suggested by antibody reactivity to contain the cleavage site. Antibody mapping of the cleavage products from *in vitro* reaction of recombinant DHCR24–FLAG and NS3-4A proteins predicted cleavage between the N- and C-terminal domains. The experimentally mapped cleavage site between Cys^91^ (*C91*) and Thr^92^ (*T92*) is indicated. *B*, to map the cleavage site, peptides spanning different regions of the putative cleavage region were cloned between an N-terminal MBP domain and a *C*-terminal GFP domain with a FLAG tag. *C*, incubation of the candidate substrate containing DHCR24 residues 56–110 with recombinant NS3-4A produced a faster-migrating band when probed with an anti-FLAG antibody. This was blocked in the presence of telaprevir, an NS3-4A inhibitor. Analogous reactions were performed with test substrates containing DHCR24 residues 52–66 or 85–99. *D*, no cleavage was observed for the DHCR24(52–66) substrate. *E*, cleavage of the DHCR24(85–99) substrate by recombinant NS3-4A was detectable by immunoblot using both anti-MBP and anti-GFP. Edman degradation of the GFP-containing peptide revealed the amino acid sequence of NH_2_-TGRPGLTG-COOH, indicating that cleavage occurs between Cys^91^ and Thr^92^. *F*, cleavage of the DHCR24(85–99) substrate was abrogated by mutation of Cys^91^ to Pro. Note that the peptide spanning map in *B* and the immunoblot in *E* are reproduced in Fig. S5 to help orient the reader in viewing the Edman degradation traces. *MW*, molecular mass.

To facilitate expression and purification of DHCR24, we fused it to a maltose-binding protein (MBP) at its N terminus. We then expressed the MBP–DHCR24 fusion with a bacteria-derived cell-free, *in vitro* translation system (33) and purified it by using magnetic amylose resin. Incubation of recombinant NS3-4A with the MBP–DHCR24 from this heterologous expression system produced a species akin to DHCR24* from HCV replicon cells (Fig. S2). Immunoblotting with an antibody that recognizes an epitope in the region between 68 and 85 of DHCR24 suggested that the cleavage site is located between the putative transmembrane region (residue 52) and the catalytic region (residue 110) of DHCR24 (Fig. 3*A*).

To specifically investigate the 56–110 region of DHCR24 as a substrate for NS3-4A, a surrogate peptide spanning this region was expressed as a fusion between the soluble proteins MBP and GFP, with a FLAG tag at the C terminus (Fig. 3*B*). Incubation of this purified test substrate with recombinant NS3-4A produces cleavage fragments (Fig. 3*C*) that are not observed in the absence of NS3-4A or in the presence of the NS3-4A inhibitor telaprevir. Next, we used two 15-mer surrogate peptides corresponding to residues 52–66 (Fig. 3*D*) and 85–99 (Fig. 3*E*) of DHCR24 to map the NS3-4A proteolysis site. Reaction of recombinant NS3-4A with the substrate spanning residues 85–99 led to two distinct products and a proportionate decrease in the full-length substrate. We purified the two product species and subjected them to N-terminal sequencing by Edman degradation. This revealed an N-terminal sequence of TGRPG, corresponding to the sequence of DHCR24 beginning at Thr^92^, and suggested that the peptide bond between residues Cys^91^ and Thr^92^ is the target of NS3-4A cleavage (Fig. 3*E*). Consistent with this hypothesis, NS3-4A is well-known to have a strong preference for Cys in the P1 site, and we found that replacement of Pro with Cys, a substitution known to inhibit proteolysis of membrane-bound enzymes by proteases (34, 35), prevented cleavage of the MBP–DHCR24(52–66)-GFP-FLAG substrate by NS3-4A *in vitro* (Fig. 3*F*).

### NS3-4A inactivation results in increased conversion of desmosterol

**Figure 4.**
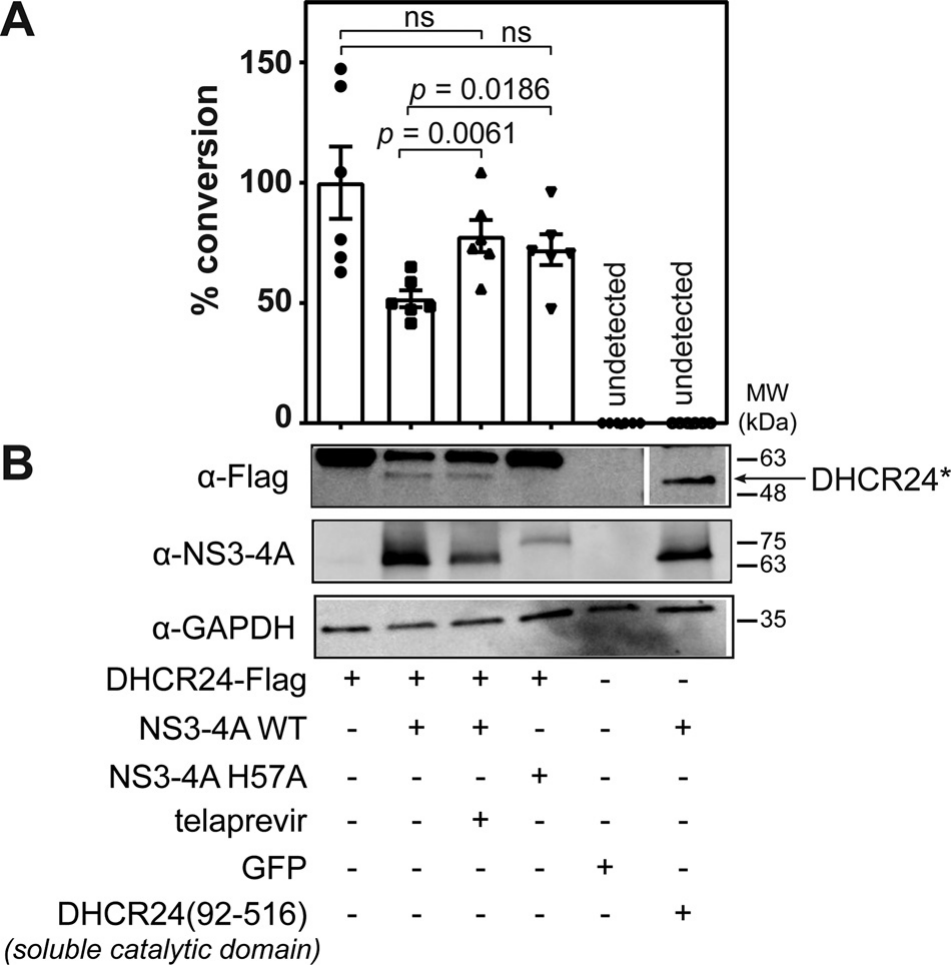
**Expression of active NS3-4A protease reduces conversion of desmosterol-d6 to cholesterol-d6 in Huh7.5-*DHCR24^KO^* cells.** Conversion of desmosterol-d6 to cholesterol-d6 and formation of DHCR24* were monitored in Huh7.5-*DHCR24^KO^* cells. The extent of conversion in Huh7.5-*DHCR24^KO^* cells expressing DHCR24 alone was set as 100%. *A*, the conversion of desmosterol-d6 to cholesterol-d6 was monitored by GC-MS analysis of extracted lipidomes. Each bar of the graph represents an average of 6 biological replicates, with *error bars* representing the standard deviation. Although no conversion of desmosterol-d6 to cholesterol-d6 is observed in the Huh7.5-*DHCR24^KO^* cells expressing a GFP control protein, ectopic expression of DHCR24-FLAG is sufficient to restore intracellular conversion of desmosterol-d6 to cholesterol-d6. Co-expression of active NS3-4A with DHCR24-FLAG reduces this reaction significantly, and this is correlated with formation of DHCR24*. NS3-4A′s effect on the intracellular reaction is abrogated in the presence of telaprevir (*p* = 0.0061) or when NS3 bears the H57A mutation in the protease active site (*p* = 0.0186). There was no significant (*ns*) difference in conversion of desmosterol-d6 to cholesterol-d6 between the Huh7.5-*DHCR24^KO^* cells expressing DHCR24 alone, telaprevir-treated cells co-expressing DHCR24 with NS3-4A (*p* = 0.2064) or cells co-expressing DHCR24 with the inactive NS3-4A H57A mutant (*p* = 0.1189). *B*, DHCR24-FLAG species were immunoprecipitated and analyzed by immunoblots with anti-FLAG and anti-NS3 antibodies. Analysis of glyceraldehyde-3-phosphate dehydrogenase (GAPDH) in cell lysates performed as an internal control is shown. Co-expression of active NS3-4A with DHCR24-FLAG results in formation of DHCR24*, which is correlated with reduced conversion of desmosterol-d6 to cholesterol-d6. DHCR24 residues 92-516, which correspond to the soluble catalytic domain, has mobility comparable to DHCR24*-FLAG, but ectopic expression of this construct does not support intracellular conversion of desmosterol-d6 to cholesterol-d6. Note that the α-FLAG blot was cut between the GFP and DHCR24(92-516) lanes to remove the intervening lane with the molecular weight ladder.

To examine the effect of proteolysis of DHCR24 by NS3-4A on the conversion of desmosterol to cholesterol in cells, we performed genome editing to “knock out” DHCR24 expression in Huh7.5 cells (Fig. S3) and then monitored the fate of desmosterol-d6 in cells ectopically expressing DHCR24–FLAG. As expected, WT DHCR24–FLAG supports conversion of desmosterol-d6 to cholesterol-d6 in Huh7.5-*DHCR24^KO^* cells. Co-expression of recombinant NS3-4A reduces the conversion of desmosterol-d6 to cholesterol-d6 to approximately half of the conversion observed in the control cells expressing only DHCR24–FLAG (Fig. 4*A*). This reduction in intracellular DHCR24 activity is furthermore associated with proteolytic cleavage of DHCR24 by the viral protease, as reflected by the appearance of a species corresponding to DHCR24*-FLAG (Fig. 4*B*). Importantly, the conversion of desmosterol-d6 to cholesterol-d6 is far less affected when telaprevir is used to inhibit NS3-4A protease activity or when the inactive NS3(H57A)-4A mutant is expressed (Fig. 4*A*). Conversion of desmosterol-d6 to cholesterol-d6 in these protease-inhibited samples does not differ in a statistically significant manner from the control samples in which DHCR24-FLAG is expressed alone without NS3-4A present. In contrast, the telaprevir-treated and NS3(H57A)-4A mutant samples do exhibit significantly reduced conversion of desmosterol-d6 to cholesterol-d6 compared to the samples in which NS3-4A and DHCR24 are co-expressed. These data thus provide a link between proteolytic activity of NS3-4A, cleavage of DHCR24, and changes in the DHCR24-catalyzed conversion of desmosterol to cholesterol.

Since cleavage of DHCR24 between Cys^91^ and Thr^92^ is predicted to separate the N-terminal membrane-associated domain from the FAD-binding and catalytic domain, we hypothesized that this might affect DHCR24-catalyzed conversion of desmosterol to cholesterol by releasing the catalytic domain from the ER membrane. Supporting this hypothesis, no conversion of desmosterol-d6 to cholesterol-d6 was observed upon ectopic expression of DHCR24(Thr^92^–His^516^) in Huh7.5-*DHCR24^KO^* cells (Fig. 4*A*). Consistent with a model in which DHCR24 is a direct substrate of the HCV NS3-4A protease *in vivo*, we detect co-occurrence of DHCR24 with NS3-4A when the NS3-5B polyprotein is expressed and processed at physiological levels in the Huh-7.5[VEEV/NS3–5B] cell culture model (36) (Fig. S4). We attempted to block cleavage of DHCR24 by mutation of Cys^91^ (C91S, C91A, C91F) but were unable to evaluate the impact of these substitutions on proteolysis by NS3-4A or HCV replication because these substitutions significantly reduce or completely inactivate DHCR24's catalytic activity (data not shown).

## Discussion

Many viruses compartmentalize their replication machinery to avoid the host defense but also to improve the efficiency of the viral process. For example, many RNA viruses, including HCV, are known to remodel sections of the host ER membrane to form specialized replication compartments (37–41). In our previous work, we have documented specific HCV-induced changes to host lipid metabolism, including a large-fold increase in desmosterol (15, 16). Enrichment of desmosterol in the membrane where viral RNA replication occurs is, moreover, correlated with an increase in the diffusivity of the membranes where RNA replication occurs (17). Prior to the work we describe here, however, the mechanism responsible for HCV's regulation of desmosterol was unknown.

**Figure 5.**
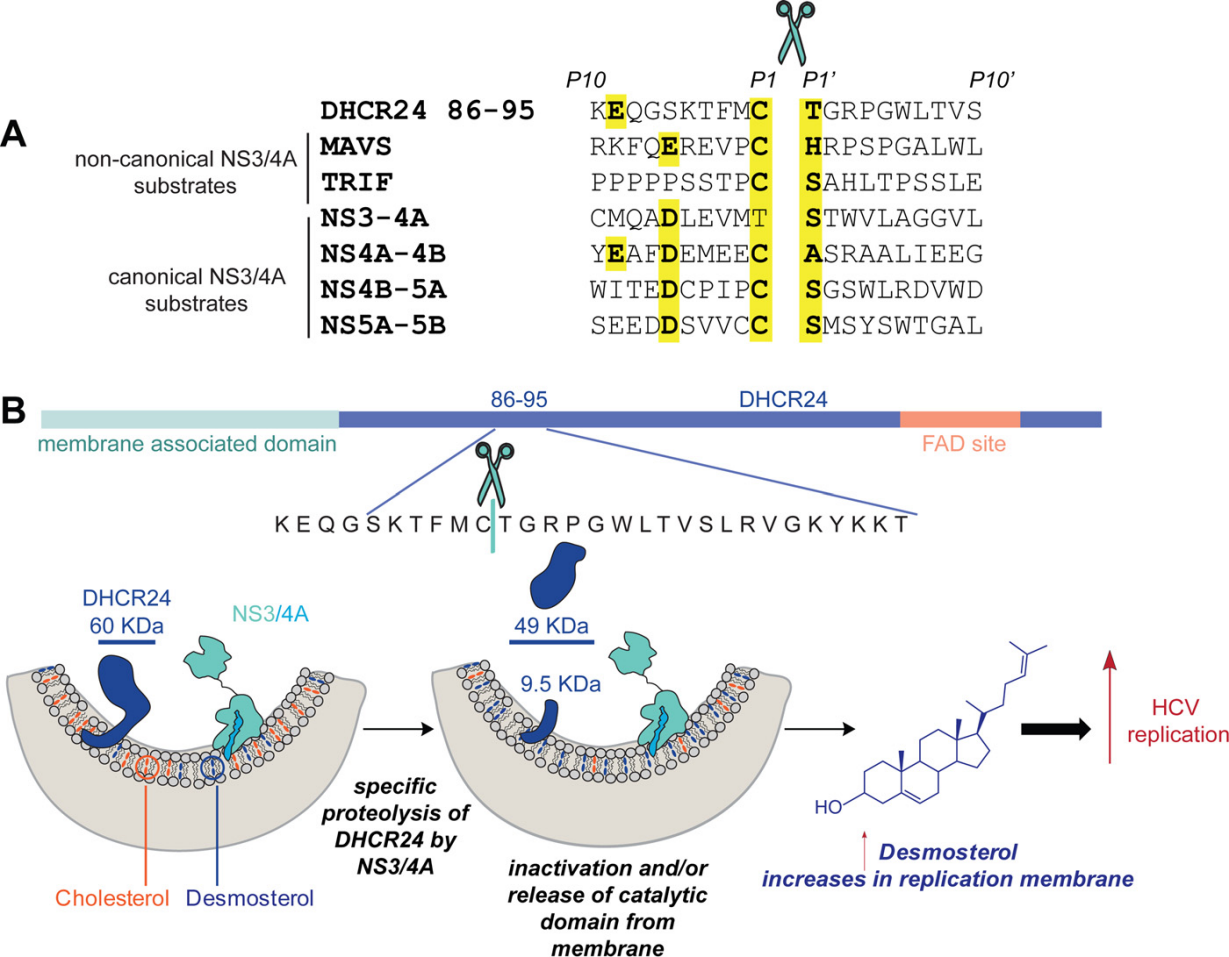
**Proposed mechanism: HCV increases the abundance of desmosterol in replication membranes through NS3-4A–mediated proteolysis of DHCR24.** *A*, sequence alignment of other known NS3-4A protein substrates with DHCR24 reveals previously unnoticed sequence similarity. *B*, during HCV replication, NS3-4A located on the ER membrane interacts with DHCR24 and cleaves it at the peptide bond between Cys^91^ and Thr^92^. This inactivates the enzyme and/or leads to its diffusion away from the membrane. The result is decreased conversion of desmosterol to cholesterol in the membrane where NS3-4A resides and where RNA replication occurs. This is associated with increased membrane diffusivity and RNA replication. HCV sequences are from HCV-J6 (genotype 2a).

We now report that HCV decreases the intracellular conversion of desmosterol to cholesterol by inactivating DHCR24, the enzyme that catalyzes this reaction, thus leading to accumulation of desmosterol. We establish that HCV inactivates DHCR24 post-translationally via targeted proteolysis. Furthermore, we demonstrate that the HCV NS3-4A protease co-occurs with DHCR24 on ER-derived membranes and/or on the ER membrane itself and cleaves DHCR24 at the peptide bond between Cys^91^ and Thr^92^ (Fig. 5). The proteolysis reaction, which we confirmed both *in vitro* and *in cellulo* under a range of conditions, thus separates the membrane-associated domain of DHCR24 from the FAD-binding and catalytic domains. The proteolytic cleavage of DHCR24 by NS3-4A may inactivate the enzyme and/or may release the enzyme from the membrane, thereby limiting its access to substrate. Both events would reduce the intracellular conversion of desmosterol to cholesterol.

Interestingly, we do not observe complete conversion of DHCR24 to DHCR24* when we probe endogenous or ectopically expressed DHCR24 in SGR cells (Fig. 2, *A–C*), when we ectopically express recombinant NS3-4A with DHCR24 (Fig. 2*D*), or in the *in vitro* reaction of immunoprecipitated DHCR24–FLAG with recombinant NS3-4A (Fig. 2*E*). Although this might reflect product inhibition of NS3-4A by DHCR24*, the absence of detectable, uncleaved polyprotein intermediates in the *in cellulo* experiments would seem to argue against this occurring to a significant extent in the context of viral replication. Another potential explanation is that not all DHCR24 is accessible to NS3-4A. This could be because only a fraction of DHCR24 co-occurs with NS3-4A and the fraction that does not cannot be cleaved to form DHCR24*. Alternatively, DHCR24 may exist in conformations or in complexes with other proteins that prevent interaction with and cleavage by NS3-4A. Our immunofluorescence imaging is consistent with the idea that DHCR24 is distributed between NS3-4A–containing and NS3-4A–lacking membranes in the cell (Fig. S4). Our prior work characterizing the HCV-induced enrichment of desmosterol in whole cell lipidomes and quantifying the enrichment of desmosterol in crude replication complexes when compared with membranes lacking the viral replicase (15, 17) is also consistent with the idea that modification of DHCR24 in these membranes is responsible for the enrichment observed in whole cell lipidomes.

We also think it worth noting that cleavage of DHCR24 may not be the only mechanism whereby HCV NS3-4A regulates desmosterol. Although in this study we utilized the appearance of DHCR24* as a biomarker for HCV-induced changes in the DHCR24-catalyzed conversion of desmosterol to cholesterol, these two phenomena are not perfectly correlated. For example, in the representative experiment depicted in Fig. 4, telaprevir-treatment is sufficient to block the effect of NS3-4A on conversion of desmosterol to cholesterol, yet some DHCR24* is observed in this sample. Although this could reflect technical issues (*e.g.* incomplete inhibition of the protease), this could also reflect NS3-4A's regulation of DHCR24 activity via a cleavage-independent mechanism. Related to this, an additional regulatory mechanism might explain why inhibition of NS3-4A activity (telaprevir, NS3-H57A mutant) may not completely restore intracellular DHCR24 activity. Additional experiments are needed to address these and other possibilities unequivocally.

Collectively, these findings support a model in which NS3-4A cleaves DHCR24 co-occurring in the replication membrane, leading to reduced conversion of desmosterol to cholesterol and, hence, accumulation of desmosterol at this site. Taken with prior work showing that HCV RNA replication is strongly correlated with desmosterol abundance in replication membranes, the specific proteolytic cleavage of DHCR24 by NS3-4A now provides a biochemical mechanism by which HCV regulates the lipid environment to support replication of the viral RNA genome. We note that NS3-4A and other HCV nonstructural proteins may further affect intracellular desmosterol (and other lipids) by mechanisms that limit access of biosynthetic enzymes to their lipid substrates or that affect enzyme structure, dynamics, and activity. Additional study to investigate these possibilities is warranted.

To our knowledge, we are the first to report that an enzyme involved in the cholesterol biosynthetic pathway is a substrate for NS3-4A. A previous study (42) reported proteolytic activation of host proteins involved in lipid metabolism induced by HCV protein expression and suggested that sterol regulatory element binding proteins, major regulators in cholesterol/fatty acid biosynthesis pathways, may be subject to regulation by HCV. The proposed mechanism, however, was through ER stress and structural changes caused by expression of HCV NS4B and core proteins (42) rather than through direct proteolytic regulation of these host proteins by NS3-4A. Thus, in addition to the complex and indirect effects of HCV on lipid biosynthesis, metabolism, and signaling, our studies show that viruses can also directly affect specific lipid species that are functionally important in viral replication.

Current HCV treatments target the viral NS3-4A serine protease, the NS5B RNA polymerase, and NS5A, which are critical for viral replication (43); however, there has been interest in targeting cholesterol biosynthesis as an alternative strategy. Statins, classically used to treat high cholesterol levels by targeting HMG-CoA reductase, have been shown to inhibit HCV replication in cell culture. Several clinical trials have examined the prospect of repurposing statins for use against HCV and other viruses (12, 44–46). Inhibition of HMG-CoA reductase affects both cholesterol synthesis and protein lipidation, both of which affect HCV replication (47–49). The disadvantage of using statins as probes or as drugs is that they inhibit the first rate-determining step in isoprenoid biosynthesis, and its product, mevalonate, is the principal component of terpenes and sterols, as well as nonisoprenoid products including heme, vitamin D, and coenzyme Q (50). Consequently, small molecules targeting HMG-CoA reductase have pleiotropic effects on cell metabolism. This makes it challenging to deconvolute which of these effects is the source of antiviral activity (Fig. 1), and increases the risk of deleterious effects mediated by off-targets. We note that Takano *et al.* (21) previously identified DHCR24 and DHCR7 as potential targets for anti-HCV drug development. This was based on findings that DHCR24 expression is augmented in liver samples from HCV-infected patients and that U18666 has an antiviral effect on HCV. Although there are differences between our studies that may be due to the complexity of mechanisms regulating sterol biosynthesis, as well as our use of different experimental models and assays, we believe that their findings are at least partially consistent with our model. First, the reduction in HCV reporter replicon activity observed in the presence of U18666A was only partially rescued by the addition of exogenous cholesterol. We posit that an unmet requirement for desmosterol may be responsible for the “unrescued” U18666A antiviral activity. Second, release or inactivation of DHCR24 by NS3-4A may trigger compensatory mechanisms (51, 52) that up-regulate DHCR24 expression in other parts of the ER under conditions of chronic liver infection.

Our work here provides insight into a specific requirement for desmosterol in HCV replication. In contrast to mevalonate, the first step in cholesterol biosynthesis that is shared with other lipid pathways, desmosterol is a penultimate and nonessential intermediate in cholesterol biosynthesis. We have identified how HCV remodels the host machinery involved in cholesterol biosynthesis through proteolysis of DHCR24 by the HCV NS3-4A protease. Going forward, elucidation of the discrete, specific lipidome changes that regulate viral RNA replication and other viral processes will continue to be important for understanding how host lipids contribute to and regulate these processes and may provide new avenues for development of specific antiviral strategies.

## Experimental procedures

Detailed methods and descriptions of plasmids are provided in the supporting information.

### Cell culture

Huh7.5 cells and Huh7.5[VEEV-NS3-5B] cells under selective pressure (puromycin at final concentration of 5 µg/ml) were cultured in Dulbecco's modified Eagle's medium supplemented with nonessential amino acids and 10% fetal bovine serum (Invitrogen) in a 37 °C incubator with 5% CO_2_ (53). Huh7.5–SGR cells stably replicating the HCV JFH1 (2a) subgenomic replicon were generated by electroporation of Huh7.5 cells with *in vitro* transcribed replicon RNA as previously described (18). Huh7.5–SGR cells were maintained in Dulbecco's modified Eagle's medium supplemented with nonessential amino acids, 10% fetal bovine serum (Invitrogen), and G418 sulfate (750 µg/ml final concentration) (15, 16, 18).

### Reverse transcription–quantitative real-time PCR

Total RNA from was isolated with TRIzol reagent (Invitrogen), and cDNA was generated using iScript reagents following the manufacturer's protocol (Bio-Rad). cDNAs were digested with RNase H, diluted 1:10 with nuclease-free water, and analyzed by real-time qPCR using the iQTM SYBR Green Supermix kit (Bio-Rad) according to the manufacturer's instructions. The reactions were run on a MyiQTM iCycler (Bio-Rad) and analyzed with the MyiQTM Optical System Software (Bio-Rad). qPCR conditions were an initial 95 °C for 5 min, followed by 40 cycles of 95 °C for 15 s and 60 °C for 30 s. Threshold cycle (*Ct*) ratios were determined by normalizing to actin and a control sample using the following equation: Ratio = 100 × 2"(*AACt*), where *AACt* = (CtHCV + gene – CtHCV + Actin) − (CtHCV − gene – CtHCV + Actin), where HCV reflects either the HCV FGR or SGR replicon. Statistical analysis was conducted using a two-tailed unpaired *t* test with a statistical significance set at a *p* value of 0.05 using GraphPad Prism release 5.0 (GraphPad Software, San Diego, CA).

### Immunoprecipitation and immunoblot analysis of DHCR7–FLAG, DHCR24–FLAG, and endogenous DHCR24

Detailed descriptions of the co-transfection experiments for ectopic expression of viral genes and DHCR24–FLAG with NS3, NS3-4A, and NS5A proteins and immunoprecipitations are provided in the supporting information. Briefly, Huh7.5 cells or Huh7.5–SGR cells were transfected with plasmids to direct ectopic expression of DHCR24-FLAG or DHCR7-FLAG. Immunoprecipitations were performed on 50 µg of protein for each of two biological replicates) using a magnetic anti-DYKDDDDK resin (Clontech) (overnight incubation at 4 °C). The samples were boiled and eluted with 1× SDS denaturing loading dye, separated by SDS-PAGE, and then transferred to a polyvinylidene difluoride membrane for immunoblotting.

### Edman degradation for N-terminal amino acid sequence determination

NS3-4A reaction products were separated by SDS-gel electrophoresis (8–16% TGX mini-protean gel, Bio-Rad). The gel was transferred onto polyvinylidene difluoride membrane (Bio-Rad) using a Bio-Rad Trans-blot Turbo apparatus (TGX mixed-MW setting: 2.5 A at 25 V for 3 min). The membrane was washed three times with LC-MS grade water (Millipore–Sigma) and stained for 30 s in 0.02% Coomassie Brilliant Blue (Sigma) in 40% methanol, 5% acetic acid for 20–30 s at room temperature and then destained in 40% methanol, 5% acetic acid for 1 min at room temperature. The membrane was rinsed in LC-MS grade water three times (5 min/rinse) and air-dried overnight. The protein bands were excised from the membrane and sequenced by automated Edman degradation on a gas phase sequencer for 5 and 10 cycles (ABI Procise 494HT instrument). There is a standard cycle of the 19 amino acids run at the beginning of each run depicting how the retention time for each amino acid is assigned. Data analysis was conducted using SequencePro version 2.1. This was conducted in single biological and technical replicate. The negative control was conducted in the absence of NS3-4A. The chromatograms are reported in Fig. S5; the peak (amino acid) in each cycle is indicated by an *arrow*.

### Measuring intracellular conversion of desmosterol-d6 to cholesterol-d6

For experiments using the HCV SGR replicon, the cells were electroporated with SGR RNA or buffer (“mock transfection”) and then immediately treated with 10 mm deuterated desmosterol (desmosterol-d6; Avanti Polar Lipids). Mock electroporated cells were additionally treated with 5 μm U18666A or an equal volume of solvent control. At 48 h post-electroporation, lipid extractions were performed, and the samples were analyzed by LC-MS or GC-MS (supporting information). Percent conversion was calculated by the following equation: (peak area cholesterol-d6)/(peak area cholesterol-d6 + peak area desmosterol-d6) × 100 = percent conversion. Statistical analysis was conducted using a two-tailed unpaired *t* test with a statistical significance set at a *p* value of 0.05 using GraphPad Prism release 5.0 (GraphPad Software). For experiments utilizing the *DHCR24^KO^* Huh7.5 cells, *DHCR24^KO^* Huh7.5 cells were transfected using Lipofectamine 2000 (Thermo Fisher Scientific). 1 µg of pIRES_DHCR24-FLAG along with 1 µg of pCMV-NS34A or 1 µg of pCMV_NS34A-H47A mutant plasmids were diluted together in 200 μl of Opti-MEM (Gibco). In a separate tube, 5 μl of Lipofectamine 2000 was added. The plasmid diluted in Opti-MEM was added to the Lipofectamine 2000–Opti-MEM mixture and incubated for 10 min at room temperature prior to addition to the cell medium. Desmosterol-d6 (Avanti Polar Lipids, 5 μm) was added immediately to all wells, and telaprevir (10 μm) was added to the NS3-4A inhibitor wells. The cells were cultured for 36 h (37 °C, 5% CO_2_) until lipid extraction and analysis by LC-MS or GC-MS as described in supporting information. Statistical analysis was conducted using a two-tailed unpaired *t* test with a statistical significance set at a *p* value of 0.05 using GraphPad Prism release 5.0 (GraphPad Software).

## Data availability

Raw data sets for the MS experiment reported in Fig. S1 have been uploaded to MassIVE (MSV000085492). Chromatograms for Edman degradation experiments are reported in Fig. S5. All other data are located in the article. Original immunoblot scans showing molecular weight markers for Figs. 1*D*, 2, and 4*B* are provided in Fig. S6. Data files for LC-MS, GC-MS, and Sanger sequencing will be shared upon request made to the corresponding author (priscilla_yang@hms.harvard.edu).

## Supplementary Material

Supporting Information
