# Supplementary material for: Hepatitis C virus NS3-4A protease regulates the lipid environment for RNA replication by cleaving host enzyme 24-dehydrocholesterol reductase
Source: J Biol Chem. 2020 Jul 8;295(35):12426–36. doi: 10.1074/jbc.RA120.013455 (PMC7458815; doi:10.1074/jbc.RA120.013455)
Supplement: Supporting Information [file supp_295_35_12426__index.html]

Hepatitis C virus NS3-4A protease regulates the lipid environment for RNA replication by cleaving host enzyme 24-dehydrocholesterol reductase — HCV NS3-4A protease regulates desmosterol metabolism — Supporting Information 

# Hepatitis C virus NS3-4A protease regulates the lipid environment for RNA replication by cleaving host enzyme 24-dehydrocholesterol reductase

## Supporting Information

- Supporting Information (to be published online) - Revised Supporting Information (clean)
