# Supplementary material for: Hepatitis C virus NS3-4A protease regulates the lipid environment for RNA replication by cleaving host enzyme 24-dehydrocholesterol reductase
Source: J Biol Chem. 2020 Jul 8;295(35):12426–36. doi: 10.1074/jbc.RA120.013455 (PMC7458815; doi:10.1074/jbc.RA120.013455)
Supplement: Supporting Information [file supp_RA120.013455_159496_3_supp_559437_qd2f31.pdf]

# Hepatitis C virus NS3-4A protease regulates the lipid environment for RNA replication by cleaving host enzyme 24-dehydrocholesterol reductase

Lorillee Tallorin**[1], Valerie A. Villareal**[,1], Chih-Yun Hsia[1], Mary A. Rodgers[1,3], Dominique J. Burri[1], Marc-Philipp Pfeil[1], Paula Montero Llopis[1], Brett D. Lindenbach[2], Priscilla L. Yang[*,1]

[1] Department of Microbiology and Blavatnik Institute, Harvard Medical School, Boston, MA 02115, USA.

[2] Department of Microbial Pathogenesis, Yale Medical School, New Haven, CT 06520, USA.

[3] Current affiliation: Abbott Laboratories, Department of Infectious Disease Research, 100 Abbott Park Rd, Abbott Park, IL 60064

* Corresponding author: Priscilla L. Yang

Email: priscilla_yang@hms.harvard.edu

** Contributed equally

**Running title:** HCV NS3-4A protease regulates desmosterol metabolism

## Supporting Information


| CONTENTS | PAGE # |
|---|---|
| **Figure S1.** Mass spectrometry analysis of transient expressed DHCR24-Flag in Huh7.5 and Huh7.5 cells containing the SGR | 2 |
| **Mass spectrometry tables for peaks A and B** | 3-4 |
| **Figure S2.** Recombinant protein NS3-4A cleaves MBP-DHCR24 in biochemical assay. | 5 |
| **Figure S3.** Characterization of Huh7.5 DHCR24$^{KO}$ cells by PCR, immunoblot, and Sanger sequencing. | 6 |
| **Figure S4.** Co-occurrence of NS3-4A and DHCR24 associated with the ER of Huh-7.5[VEEV/NS3–5B] cells | 7 |
| **Figure S5.** Edman degradation experiment and chromatograms | 8-24 |
| **Figure S6.** Original immunoblot scans showing molecular weight markers for Figures 1D, 2, and 4B | 25-27 |
| **Detailed Experimental Procedures** | 28-34 |
| **References for Supporting Information** | 35 |

**Figure S1. Mass spectrometry analysis of DHCR24 and DHCR24* isolated from Huh7.5 and Huh7.5-SGR cells**. Samples were separated by SDS-PAGE. One half of the gel was silver-stained (left) and peptides (marked by arrows) were excised for mass spectrometry. The other half of the gel (right) was transferred to PVDF membrane and probed with anti-Flag antibody as described in Experimental Procedures. The molecular weight markers were used to align the two images. Mass spectrometry analysis of the excised bands, marked "a" and "b," detected multiple peptides derived from DHCR24. Lower signal was detected for peptides prior to residue 113, but a definitive cleavage site for the "b" sample could not be identified. Peak intensities for the indicated N-terminal charged peptide fragment ions in the "a" and "b" samples are reported in tables on pages 3 and 4. Raw data sets have been uploaded to MassIVE (accession number MSV000085492.

| | | peak intensity | |
|---|---|---|---|
| **Peptide** | **residues** | **a - 46739** | **b - 46740** |
| GLEFVLIHQR | 22-31 | 8.01E+04 | 2.37E+04 |
| AWVVFK | 54-59 | 3.51E+06 | 1.56E+05 |
| VRDIQK | 71-76 | 1.53E+06 | 3.19E+05 |
| NIMINLMDILEVDTKK | 113-128 | 4.00E+06 | 3.94E+06 |
| HVENYLK | 307-313 | 1.08E+08 | 4.11E+07 |
| LISEEDLAANDILDYKDDDDKV | 526-547 | 7.97E+07 | 4.08E+07 |

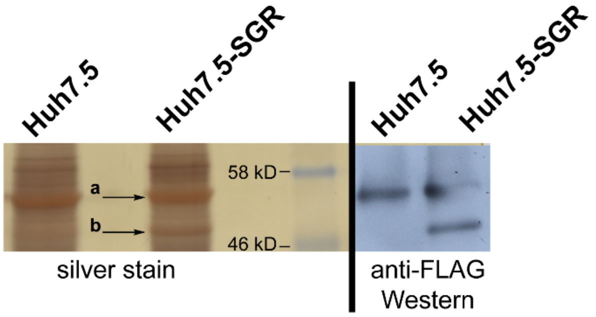

**Figure S1 continued**

**DHCR24 Peak A**

| Protein ID | Obs m/z | z | XCorr | Peptide | Missed Cleavage | Modification |
|---|---|---|---|---|---|---|
| DHRC24 | 619.3264 | 2 | 3.572 | R.YLFGWMVPPK.I | 0 | |
| DHRC24 | 603.3447 | 3 | 1.471 | R.YLFGWM*VPPKISLLK.L | 1 | M - Oxidation |
| DHRC24 | 627.3240 | 2 | 3.550 | R.YLFGWM*VPPK.I | 0 | M - Oxidation |
| DHRC24 | 571.3442 | 2 | 2.377 | R.VRDIQKQVR.E | 2 | |
| DHRC24 | 379.7295 | 2 | 2.202 | R.VRDIQK.Q | 1 | |
| DHRC24 | 443.7479 | 2 | 1.842 | R.VKHFEAR.S | 1 | |
| DHRC24 | 436.2536 | 2 | 2.381 | R.TRPLEQK.L | 0 | |
| DHRC24 | 669.9560 | 3 | 4.026 | R.SVHGFQMLYADCYM*NR.E | 0 | M - Oxidation |
| DHRC24 | 669.9563 | 3 | 4.278 | R.SVHGFQM*LYADCYMNR.E | 0 | M - Oxidation |
| DHRC24 | 675.2881 | 3 | 4.305 | R.SVHGFQM*LYADCYM*NR.E | 0 | M - Oxidation |
| DHRC24 | 460.2716 | 2 | 1.387 | R.QLEKFVR.S | 1 | |
| DHRC24 | 699.0340 | 3 | 4.124 | R.KLYEQHHVVQDMLVPMK.C | 0 | |
| DHRC24 | 704.3654 | 3 | 3.116 | R.KLYEQHHVVQDMLVPM*K.C | 0 | M - Oxidation |
| DHRC24 | 528.5256 | 4 | 2.666 | R.KLYEQHHVVQDM*LVPMK.C | 0 | M - Oxidation |
| DHRC24 | 709.6968 | 3 | 3.195 | R.KLYEQHHVVQDM*LVPM*K.C | 0 | M - Oxidation |
| DHRC24 | 388.1853 | 2 | 1.886 | R.HYYHR.H | 0 | |
| DHRC24 | 431.2283 | 2 | 2.661 | R.GLEAICAK.F | 0 | |
| DHRC24 | 497.2661 | 3 | 2.100 | R.FEPVRGLEAICAK.F | 1 | |
| DHRC24 | 496.2462 | 2 | 1.677 | R.EWKEQGSK.T | 1 | |
| DHRC24 | 734.0204 | 3 | 3.514 | R.EKLGCQDAFPEVYDKICK.A | 2 | |
| DHRC24 | 600.2821 | 3 | 2.986 | R.EKLGCQDAFPEVYDK.I | 1 | |
| DHRC24 | 747.3853 | 3 | 1.897 | R.EGLEYIPLRHYYHRHTR.S | 2 | |
| DHRC24 | 615.9855 | 3 | 1.663 | R.EGLEYIPLRHYYHR.H | 1 | |
| DHRC24 | 545.2997 | 2 | 3.095 | R.EGLEYIPLR.H | 0 | |
| DHRC24 | 606.6004 | 3 | 1.368 | R.EEFWEMFDGSLYHK.L | 0 | |
| DHRC24 | 611.9322 | 3 | 2.274 | R.EEFWEM*FDGSLYHK.L | 0 | M - Oxidation |
| DHRC24 | 375.2207 | 2 | 2.412 | R.AWVVFK.L | 0 | |
| DHRC24 | 436.2594 | 3 | 1.574 | K.YVKLRFEPVR.G | 2 | |
| DHRC24 | 853.7638 | 3 | 3.039 | K.YGLFQHICTAYELVLADGSFVR.C | 0 | |
| DHRC24 | 487.6003 | 3 | 3.601 | K.TNREGLEYIPLR.H | 1 | |
| DHRC24 | 568.8072 | 4 | 3.260 | K.THKNIMINLM*DILEVDTKK.Q | 1 | M - Oxidation |
| DHRC24 | 763.4065 | 3 | 2.966 | K.THKNIM*INLM*DILEVDTKK.Q | 1 | M - Oxidation |
| DHRC24 | 687.8608 | 2 | 3.057 | K.QVREWKEQGSK.T | 2 | |
| DHRC24 | 636.0069 | 3 | 2.129 | K.NIMINLM*DILEVDTKK.Q | 0 | M - Oxidation |
| DHRC24 | 889.4600 | 2 | 5.273 | K.NIMINLM*DILEVDTK.K | 0 | M - Oxidation |
| DHRC24 | 636.0068 | 3 | 1.705 | K.NIM*INLMDILEVDTKK.Q | 0 | M - Oxidation |
| DHRC24 | 641.3383 | 3 | 2.652 | K.NIM*INLM*DILEVDTKK.Q | 0 | M - Oxidation |
| DHRC24 | 897.4567 | 2 | 5.556 | K.NIM*INLM*DILEVDTK.K | 0 | M - Oxidation |
| DHRC24 | 656.3351 | 3 | 4.274 | K.LYEQHHVVQDMLVPMK.C | 0 | |
| DHRC24 | 661.6669 | 3 | 4.128 | K.LYEQHHVVQDMLVPM*K.C | 0 | M - Oxidation |
| DHRC24 | 661.6665 | 3 | 4.083 | K.LYEQHHVVQDM*LVPMK.C | 0 | M - Oxidation |
| DHRC24 | 666.9984 | 3 | 4.001 | K.LYEQHHVVQDM*LVPM*K.C | 0 | M - Oxidation |
| DHRC24 | 523.3033 | 2 | 2.648 | K.LTQGETLRK.L | 0 | |
| DHRC24 | 459.2550 | 2 | 2.387 | K.LTQGETLR.K | 0 | |
| DHRC24 | 586.9951 | 3 | 3.231 | K.LRFEPVRGLEAICAK.F | 2 | |
| DHRC24 | 458.7714 | 2 | 2.138 | K.LRFEPVR.G | 1 | |
| DHRC24 | 592.9760 | 3 | 1.404 | K.LNSIGNYYKPWFFK.H | 0 | |
| DHRC24 | 837.0683 | 3 | 4.708 | K.LISEEDLAANDILDYKDDDDKV.- | 2 | |
| DHRC24 | 804.0453 | 3 | 3.100 | K.LISEEDLAANDILDYKDDDDK.V | 1 | |
| DHRC24 | 911.4618 | 2 | 5.347 | K.LISEEDLAANDILDYK.D | 0 | |
| DHRC24 | 863.7204 | 3 | 2.851 | K.LIS#EEDLAANDILDYKDDDDKV.- | 2 | S - Phosphoryl. |
| DHRC24 | 561.0259 | 4 | 2.029 | K.LGCQDAFPEVYDKICKAAR.H | 2 | |
| DHRC24 | 648.3071 | 3 | 3.928 | K.LGCQDAFPEVYDKICK.A | 1 | |
| DHRC24 | 771.3503 | 2 | 4.288 | K.LGCQDAFPEVYDK.I | 0 | |
| DHRC24 | 533.9984 | 3 | 2.429 | K.ISLLKLTQGETLRK.L | 1 | |
| DHRC24 | 491.3000 | 3 | 3.121 | K.ISLLKLTQGETLR.K | 1 | |
| DHRC24 | 425.2260 | 3 | 1.964 | K.HVENYLKTNR.E | 1 | |
| DHRC24 | 451.7391 | 2 | 3.191 | K.HVENYLK.T | 0 | |
| DHRC24 | 933.9473 | 2 | 5.450 | K.GNEAELYIDIGAYGEPR.V | 0 | |
| DHRC24 | 404.5680 | 3 | 2.328 | K.GLEFVLIHQR.W | 0 | |
| DHRC24 | 452.7174 | 2 | 2.318 | K.FTHESQR.Q | 0 | |

**Figure S1 continued**

**DHCR24 Peak B**

| Protein ID | Obs m/z | z | XCorr | Peptide | Missed Cleavage | Modification |
|---|---|---|---|---|---|---|
| DHRC24 | 452.7172 | 2 | 2.329 | K.FTHESQR.Q | 0 | |
| DHRC24 | 404.5678 | 3 | 2.239 | K.GLEFVLIHQR.W | 0 | |
| DHRC24 | 933.9495 | 2 | 5.510 | K.GNEAELYIDIGAYGEPR.V | 0 | |
| DHRC24 | 451.7399 | 2 | 3.277 | K.HVENYLK.T | 0 | |
| DHRC24 | 491.2999 | 3 | 2.517 | K.ISLLKLTQGETLR.K | 1 | |
| DHRC24 | 771.3510 | 2 | 4.475 | K.LGCQDAFPEVYDK.I | 0 | |
| DHRC24 | 863.7235 | 3 | 3.200 | K.LIS#EEDLAANDILDYKDDDDKV.- | 2 | S - Phosphoryl. |
| DHRC24 | 863.7207 | 3 | 2.941 | K.LISEEDLAANDILDY#KDDDDKV.- | 2 | Y - Phosphoryl. |
| DHRC24 | 911.4619 | 2 | 5.262 | K.LISEEDLAANDILDYK.D | 0 | |
| DHRC24 | 804.0450 | 3 | 3.418 | K.LISEEDLAANDILDYKDDDDK.V | 1 | |
| DHRC24 | 837.0670 | 3 | 3.944 | K.LISEEDLAANDILDYKDDDDKV.- | 2 | |
| DHRC24 | 592.9760 | 3 | 1.972 | K.LNSIGNYYKPWFFK.H | 0 | |
| DHRC24 | 665.8485 | 4 | 1.975 | K.LNSIGNYYKPWFFKHVENYLK.T | 1 | |
| DHRC24 | 458.7719 | 2 | 2.164 | K.LRFEPVR.G | 1 | |
| DHRC24 | 459.2557 | 2 | 2.306 | K.LTQGETLR.K | 0 | |
| DHRC24 | 523.3037 | 2 | 2.156 | K.LTQGETLRK.L | 0 | |
| DHRC24 | 666.9981 | 3 | 4.060 | K.LYEQHHVVQDM*LVPM*K.C | 0 | M - Oxidation |
| DHRC24 | 661.6667 | 3 | 3.766 | K.LYEQHHVVQDM*LVPMK.C | 0 | M - Oxidation |
| DHRC24 | 656.3352 | 3 | 4.429 | K.LYEQHHVVQDMLVPMK.C | 0 | |
| DHRC24 | 897.4580 | 2 | 4.341 | K.NIM*INLM*DILEVDTK.K | 0 | M - Oxidation |
| DHRC24 | 641.3384 | 3 | 3.143 | K.NIM*INLM*DILEVDTKK.Q | 0 | M - Oxidation |
| DHRC24 | 953.5079 | 2 | 3.457 | K.NIM*INLMDILEVDTKK.Q | 0 | M - Oxidation |
| DHRC24 | 889.4602 | 2 | 5.421 | K.NIMINLM*DILEVDTK.K | 0 | M - Oxidation |
| DHRC24 | 636.0068 | 3 | 1.548 | K.NIMINLM*DILEVDTKK.Q | 0 | M - Oxidation |
| DHRC24 | 487.6007 | 3 | 3.485 | K.TNREGLEYIPLR.H | 1 | |
| DHRC24 | 853.7640 | 3 | 2.916 | K.YGLFQHICTAYELVLADGSFVR.C | 0 | |
| DHRC24 | 436.2593 | 3 | 1.673 | K.YVKLRFEPVR.G | 2 | |
| DHRC24 | 375.2209 | 2 | 2.346 | R.AWVVFK.L | 0 | |
| DHRC24 | 611.9324 | 3 | 2.331 | R.EEFWEM*FDGSLYHK.L | 0 | M - Oxidation |
| DHRC24 | 606.6003 | 3 | 2.839 | R.EEFWEMFDGSLYHK.L | 0 | |
| DHRC24 | 545.3007 | 2 | 3.068 | R.EGLEYIPLR.H | 0 | |
| DHRC24 | 747.3849 | 3 | 1.921 | R.EGLEYIPLRHYYHRHTR.S | 2 | |
| DHRC24 | 600.2829 | 3 | 2.361 | R.EKLGCQDAFPEVYDK.I | 1 | |
| DHRC24 | 497.2664 | 3 | 1.203 | R.FEPVRGLEAICAK.F | 1 | |
| DHRC24 | 431.2285 | 2 | 2.736 | R.GLEAICAK.F | 0 | |
| DHRC24 | 388.1852 | 2 | 1.758 | R.HYYHR.H | 0 | |
| DHRC24 | 532.5242 | 4 | 2.542 | R.KLYEQHHVVQDM*LVPM*K.C | 0 | M - Oxidation |
| DHRC24 | 704.3655 | 3 | 2.772 | R.KLYEQHHVVQDM*LVPMK.C | 0 | M - Oxidation |
| DHRC24 | 524.5266 | 4 | 2.084 | R.KLYEQHHVVQDMLVPMK.C | 0 | |
| DHRC24 | 1162.214 | 3 | 2.873 | R.QENHFVEGLLYSLDEAVIM*TGVM*TDEAEPSK.L | 0 | M - Oxidation |
| DHRC24 | 675.2881 | 3 | 4.015 | R.SVHGFQM*LYADCYM*NR.E | 0 | M - Oxidation |
| DHRC24 | 1004.433 | 2 | 4.883 | R.SVHGFQM*LYADCYMNR.E | 0 | M - Oxidation |
| DHRC24 | 664.6248 | 3 | 4.176 | R.SVHGFQMLYADCYMNR.E | 0 | |
| DHRC24 | 436.2535 | 2 | 2.203 | R.TRPLEQK.L | 0 | |
| DHRC24 | 379.7295 | 2 | 1.522 | R.VRDIQK.Q | 1 | |
| DHRC24 | 627.3239 | 2 | 3.264 | R.YLFGWM*VPPK.I | 0 | M - Oxidation |
| DHRC24 | 619.3265 | 2 | 3.722 | R.YLFGWMVPPK.I | 0 | |

**Figure S2. Recombinant NS3-4A cleaves MBP-DHCR24 in an *in vitro* biochemical assay***.* The MBP-DHCR24 protein was produced by heterologous expression in an *E. coli*- derived i*n vitro* translation system. Incubation of the MBP-DHCR24 fusion protein with recombinant NS3-4A resulted in formation of a faster-migrating species (red arrow head). This reaction was blocked in the presence of telaprevir ("inh").

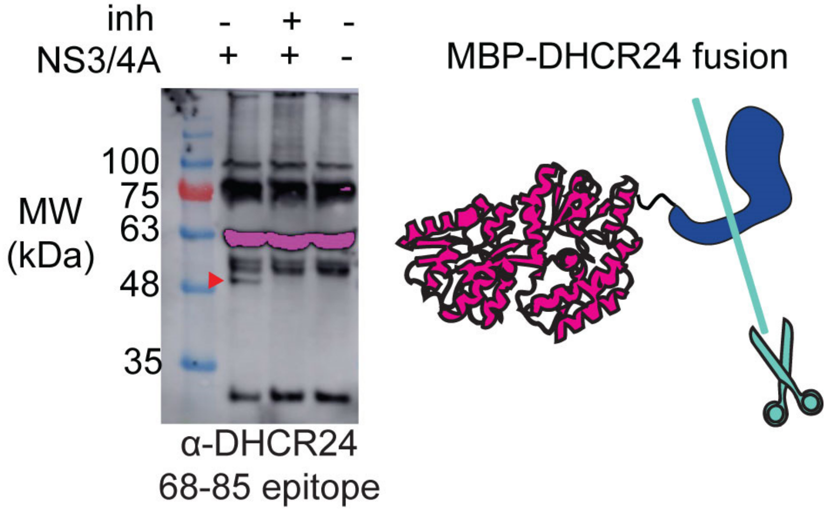

**Figure S3. Characterization of Huh7.5 DHCR24$^{KO}$ cells by PCR, immunoblot, and Sanger sequencing.** A) Genomic DNA from Huh7.5 CRISPR-Cas9 knockout of DHCR24 (clone 3) shows no band corresponding to wild type (WT) DHCR24 in PCR assays using forward primer: 5'-AGA TGC CTA GTG TGT TGG GAA T-3' and reverse primer: 5'-AAA GGG TTC CCC TGA CCT ATT A-3'. B) Immunoblot analysis of lysates from candidate Huh7.5 DHCR24$^{KO}$ clones using an N-terminal DHCR24 antibody (residues 68-85). No band was observed for clone 3. C) Nucleotide region of WT DHCR24 between exon 1 and 2. D) Region between exon 1 and 2 excised by CRISPR-Cas9. E) Sanger sequencing results of clone 3 band from agarose gel (shown in A) depicting removal of the designated region, resulting in removal of ~280 nucleotide bases in DHCR24 in clone 3. This clonal cell line is referred to as Huh7.5-DHCR24$^{KO}$ in the text.

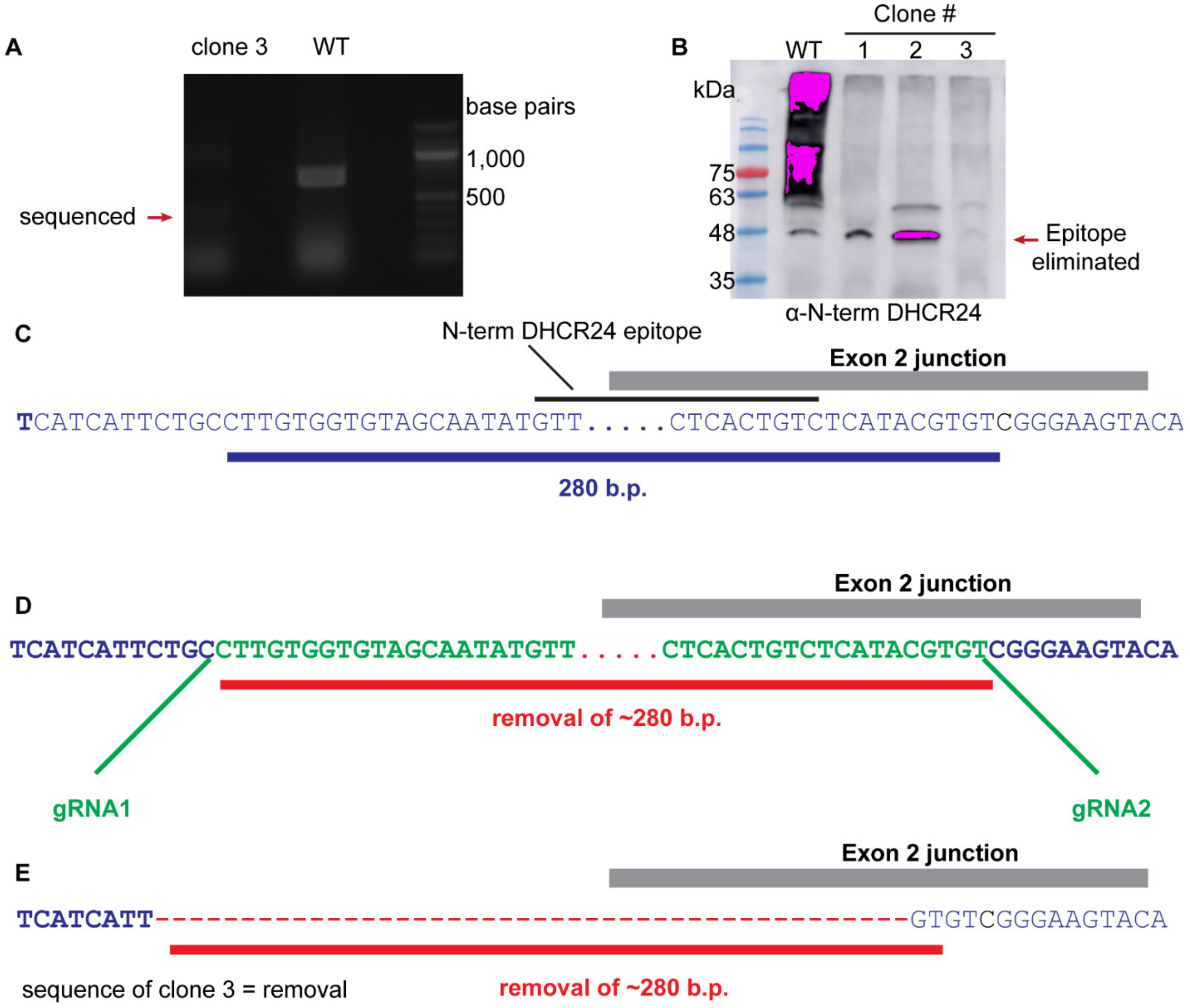

**Figure S4. Co-occurrence of NS3-4A and DHCR24 associated with the ER of Huh-7.5[VEEV/NS3–5B] cells.** Immunofluorescence detection of NS3-4A, DHCR24, and the ER marker protein disulfide isomerase (PDI) in Huh-7.5[VEEV/NS3–5B] cells. These cells have been transduced with a noncytopathic subgenomic replicon derived from Venezuelan equine encephalitis virus. They are an established system for expression of the NS3-5B polyprotein at physiological levels with appropriate polyprotein processing and allow for studies of protein expression, processing, and localization over several weeks when maintained under selective conditions (36). A) DHCR24 (magenta) and NS3-4A (green) were observed as a fraction co-occurring in the endoplasmic reticulum (ER). B) DHCR24 (magenta, gamma = 1.2) and the ER marker protein disulfide isomerase (PDI, green, gamma=1.4) co-occur in the ER. Insert scale bar = 10 μm.

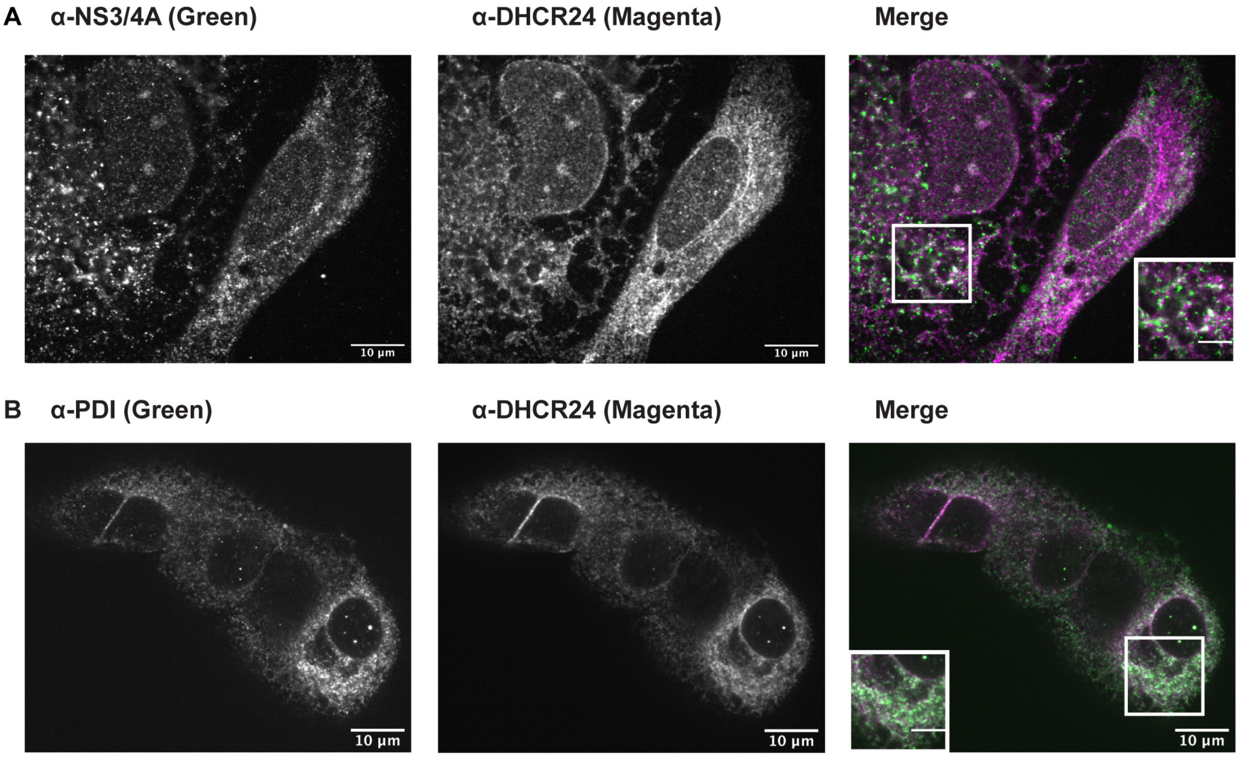

**Figure S5. Edman degradation chromatograms of cleaved DHCR24 by NS3-4A.** A) Shows MBP portion of MBP-85-99-DHCR24-GFP-FlagTag completed in 5 cycles with the residue calls in order from 1-5. Traces are shown on pages 8-13. B) GFP portion of MBP-85-99-DHCR24-GFP-FlagTag completed in 10 cycles with residue calls from 1-10. Cycle 6 and 10 are unassigned. B) begins on page 14 and continues through page 24. The peptide spanning map and immunoblots from Figure 3B have been reproduced here in order to orient the reader in viewing the Edman degradation traces.

## A. STANDARD

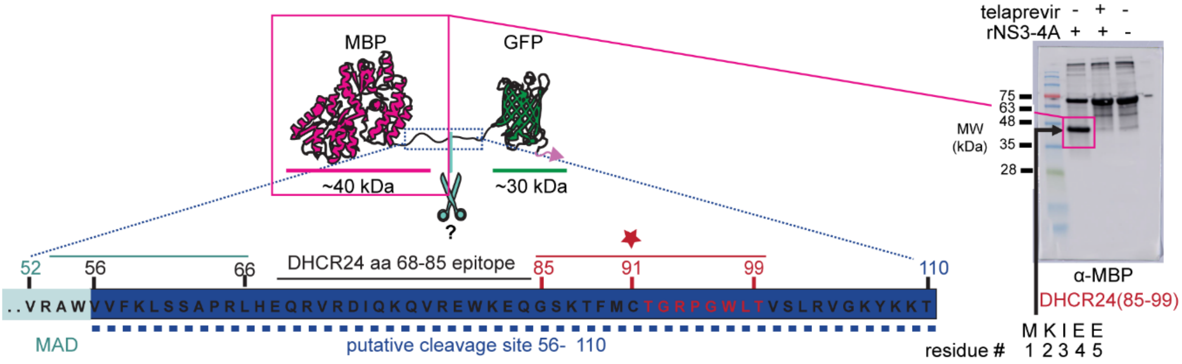


SequencePro™

Tufts Core Facility
Tufts Medical School
136 Harrison Ave
Boston, MA 02111

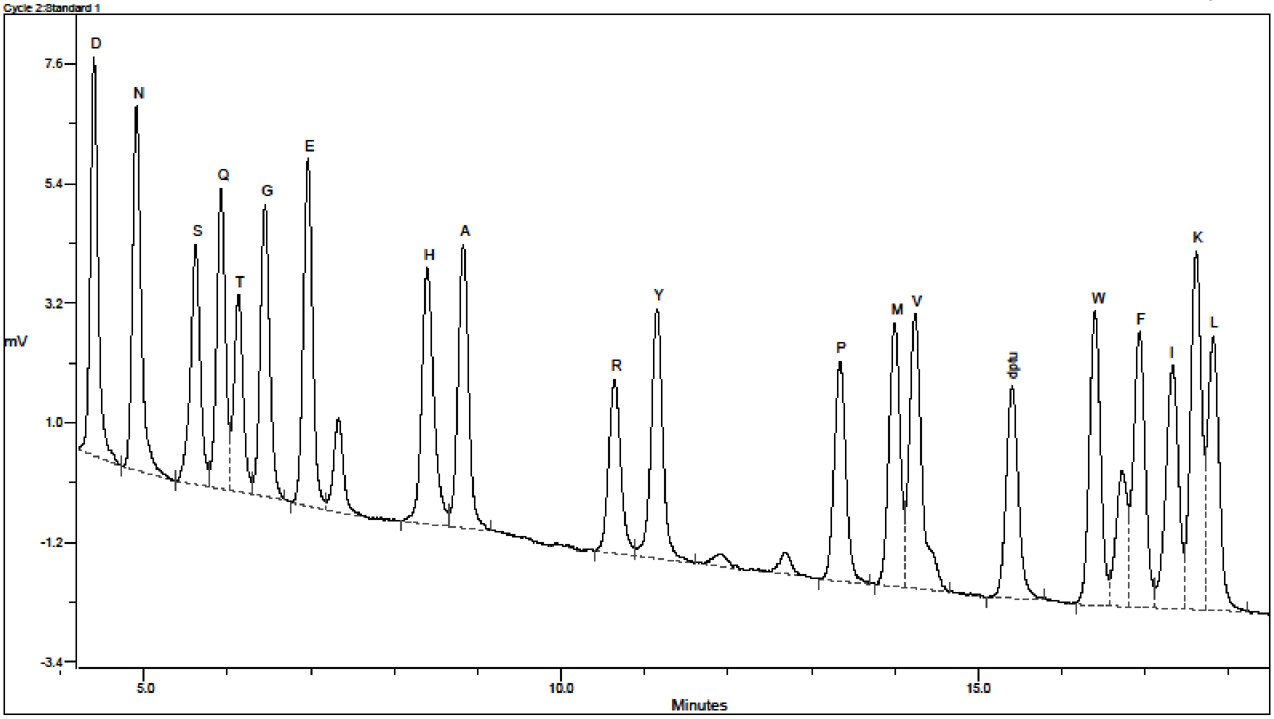


| PEAK ID | R.TIME (mins) | C.TIME (mins) | HEIGHT (mV) | PMOL HT | PEAK ID | R.TIME (mins) | C.TIME (mins) | HEIGHT (mV) | PMOL HT |
|---|---|---|---|---|---|---|---|---|---|
| D | 4.40 | 4.40 | 7.334 | 10.000 | Y | 11.15 | 11.15 | 4.590 | 10.000 |
| N | 4.91 | 4.91 | 6.698 | 10.000 | P | 13.34 | 13.34 | 4.035 | 10.000 |
| S | 5.62 | 5.62 | 4.409 | 10.000 | M | 14.00 | 14.00 | 4.839 | 10.000 |
| Q | 5.92 | 5.92 | 5.525 | 10.000 | V | 14.24 | 14.24 | 5.044 | 10.000 |
| T | 6.13 | 6.13 | 3.618 | 10.000 | dptu | 15.40 | 15.40 | 3.895 | 10.000 |
| G | 6.45 | 6.45 | 5.375 | 10.000 | W | 16.40 | 16.40 | 5.408 | 10.000 |
| E | 6.97 | 6.97 | 6.384 | 10.000 | F | 16.93 | 16.93 | 5.058 | 10.000 |
| H | 8.40 | 8.40 | 4.708 | 10.000 | I | 17.33 | 17.33 | 4.465 | 10.000 |
| A | 8.83 | 8.83 | 5.207 | 10.000 | K | 17.61 | 17.61 | 6.595 | 10.000 |
| R | 10.64 | 10.64 | 3.205 | 10.000 | L | 17.82 | 17.82 | 5.042 | 10.000 |

Wednesday, March 13, 2019 08:43:08

311LT-2 - Page 2 of 8

## Figure S5. A) continued

SequencePro™

Tufts Core Facility
Tufts Medical School
136 Harrison Ave
Boston, MA 02111

Cycle 3:Residue 1

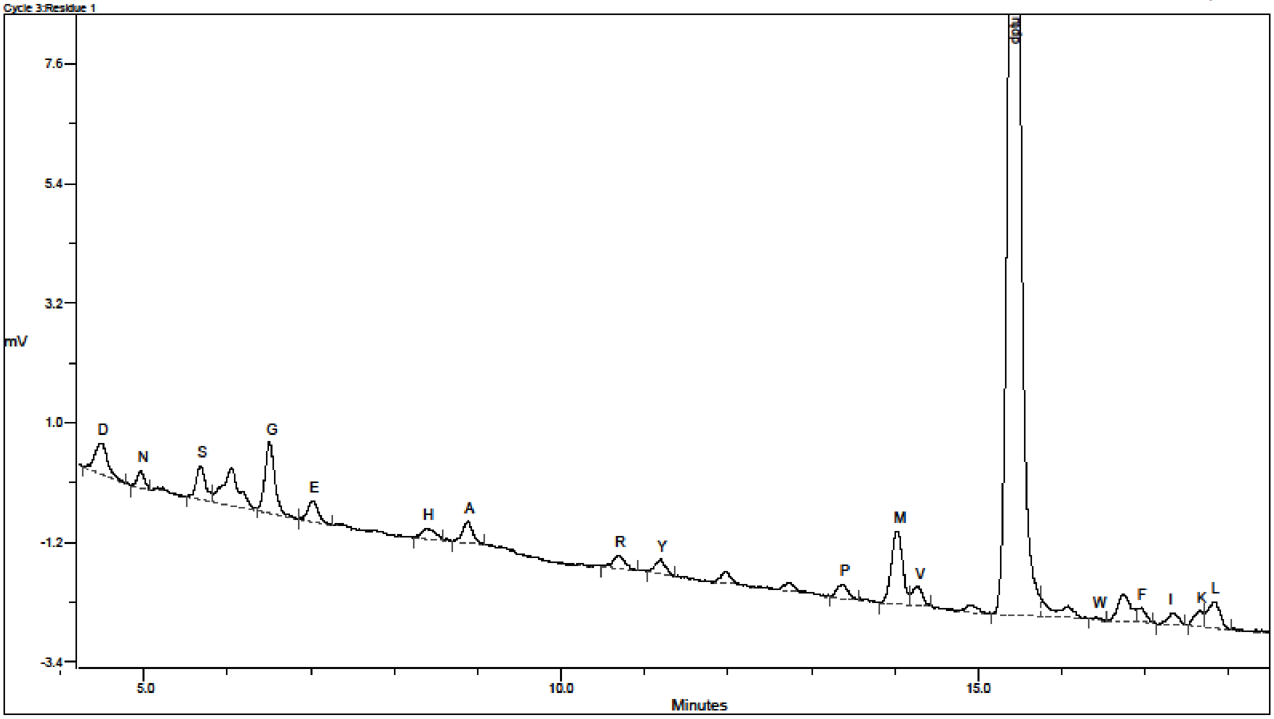


| PEAK ID | R.TIME (mins) | C.TIME (mins) | HEIGHT (mV) | PMOL HT |
|---|---|---|---|---|
| D | 4.49 | 4.40 | 0.570 | 0.777 |
| N | 4.97 | 4.91 | 0.326 | 0.486 |
| S | 5.68 | 5.62 | 0.624 | 1.414 |
| G | 6.50 | 6.45 | 1.304 | 2.426 |
| E | 7.02 | 6.97 | 0.387 | 0.606 |
| H | 8.39 | 8.40 | 0.197 | 0.419 |
| A | 8.88 | 8.83 | 0.404 | 0.776 |
| R | 10.69 | 10.64 | 0.236 | 0.736 |
| Y | 11.19 | 11.15 | 0.263 | 0.574 |
| P | 13.38 | 13.34 | 0.262 | 0.650 |

| PEAK ID | R.TIME (mins) | C.TIME (mins) | HEIGHT (mV) | PMOL HT |
|---|---|---|---|---|
| M | 14.03 | 14.00 | 1.349 | 2.788 |
| V | 14.28 | 14.24 | 0.345 | 0.684 |
| dptu | 15.43 | 15.40 | 19.343 | 49.666 |
| W | 16.41 | 16.40 | 0.051 | 0.095 |
| F | 16.95 | 16.93 | 0.265 | 0.524 |
| I | 17.32 | 17.33 | 0.215 | 0.482 |
| K | 17.66 | 17.61 | 0.288 | 0.436 |
| L | 17.83 | 17.82 | 0.482 | 0.956 |

Wednesday, March 13, 2019 08:43:08

311LT-2 - Page 3 of 8

**RESIDUE #1 = M**

## Figure S5. A) continued

SequencePro™

Tufts Core Facility
Tufts Medical School
136 Harrison Ave
Boston, MA 02111

Cycle 4:Residue 2

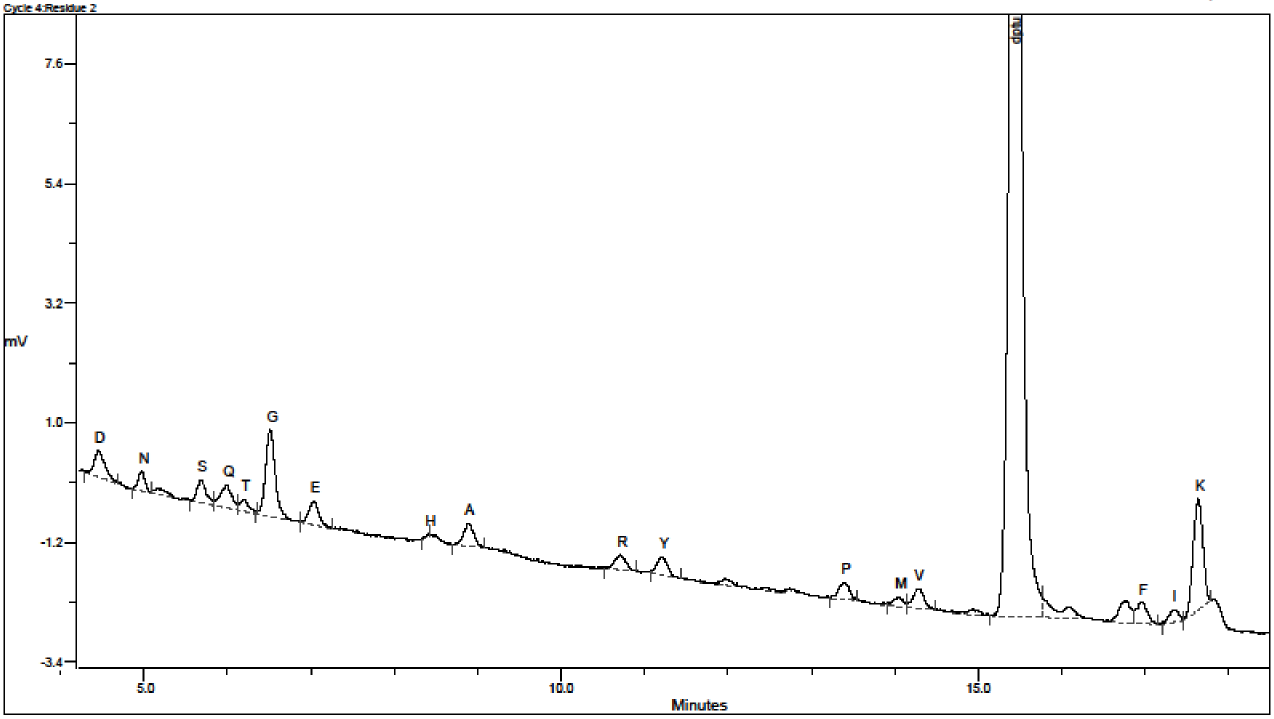


| PEAK ID | R.TIME (mins) | C.TIME (mins) | HEIGHT (mV) | PMOL HT |
|---|---|---|---|---|
| D | 4.45 | 4.40 | 0.479 | 0.653 |
| N | 4.97 | 4.91 | 0.357 | 0.533 |
| S | 5.68 | 5.62 | 0.411 | 0.932 |
| Q | 5.99 | 5.92 | 0.429 | 0.777 |
| T | 6.20 | 6.13 | 0.214 | 0.592 |
| G | 6.51 | 6.45 | 1.589 | 2.956 |
| E | 7.03 | 6.97 | 0.443 | 0.693 |
| H | 8.41 | 8.40 | 0.047 | 0.100 |
| A | 8.88 | 8.83 | 0.409 | 0.786 |
| R | 10.71 | 10.64 | 0.280 | 0.874 |
| Y | 11.21 | 11.15 | 0.329 | 0.718 |
| P | 13.40 | 13.34 | 0.297 | 0.735 |
| M | 14.04 | 14.00 | 0.169 | 0.348 |
| V | 14.27 | 14.24 | 0.357 | 0.708 |
| dptu | 15.45 | 15.40 | 20.367 | 52.297 |
| F | 16.96 | 16.93 | 0.381 | 0.754 |
| I | 17.36 | 17.33 | 0.228 | 0.511 |
| K | 17.64 | 17.61 | 2.038 | 3.090 |

Wednesday, March 13, 2019 08:43:08

311LT-2 - Page 4 of 8

**RESIDUE #2 = K**

## Figure S5. A) continued

SequencePro™

Tufts Core Facility
Tufts Medical School
136 Harrison Ave
Boston, MA 02111

Cycle 5:Residue 3

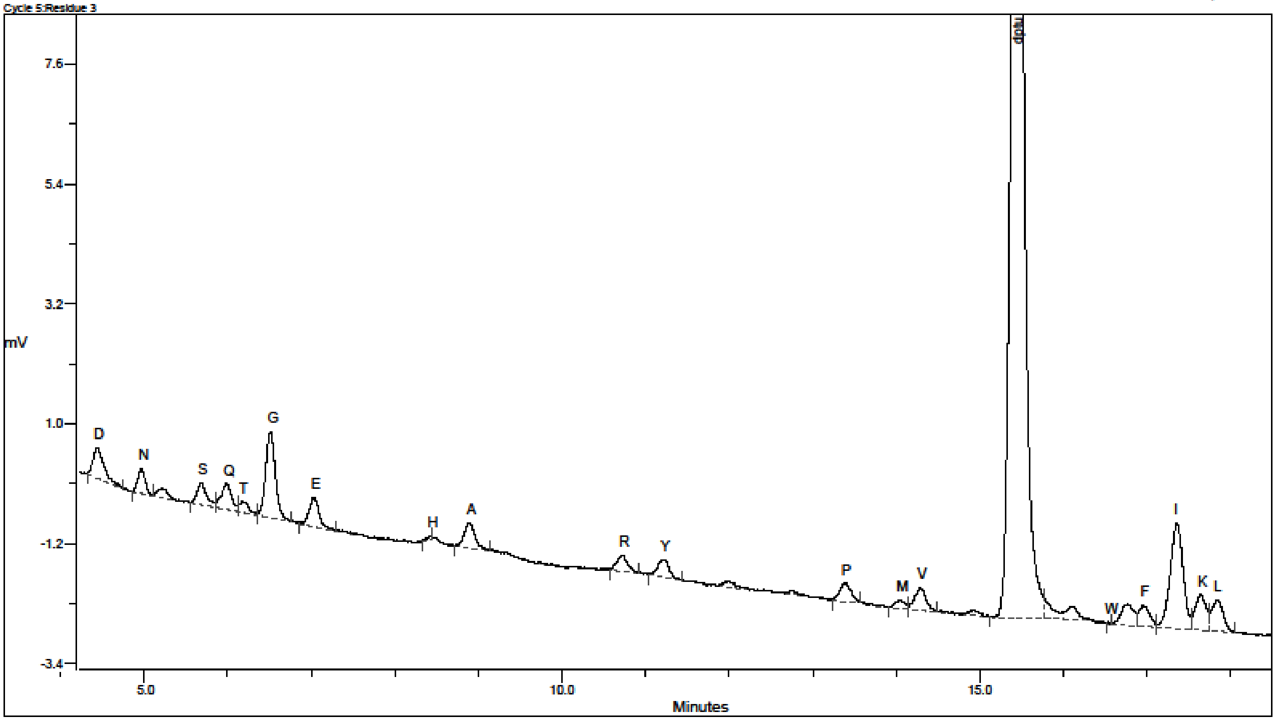


| PEAK ID | R.TIME (mins) | C.TIME (mins) | HEIGHT (mV) | PMOL HT |
|---|---|---|---|---|
| D | 4.43 | 4.40 | 0.566 | 0.772 |
| N | 4.97 | 4.91 | 0.452 | 0.674 |
| S | 5.68 | 5.62 | 0.405 | 0.918 |
| Q | 5.99 | 5.92 | 0.472 | 0.854 |
| T | 6.17 | 6.13 | 0.196 | 0.543 |
| G | 6.51 | 6.45 | 1.580 | 2.940 |
| E | 7.03 | 6.97 | 0.537 | 0.842 |
| H | 8.43 | 8.40 | 0.049 | 0.103 |
| A | 8.89 | 8.83 | 0.462 | 0.887 |
| R | 10.73 | 10.64 | 0.291 | 0.910 |

| PEAK ID | R.TIME (mins) | C.TIME (mins) | HEIGHT (mV) | PMOL HT |
|---|---|---|---|---|
| Y | 11.22 | 11.15 | 0.316 | 0.688 |
| P | 13.38 | 13.34 | 0.336 | 0.832 |
| M | 14.04 | 14.00 | 0.142 | 0.294 |
| V | 14.29 | 14.24 | 0.422 | 0.837 |
| dptu | 15.45 | 15.40 | 22.281 | 57.210 |
| W | 16.53 | 16.40 | 0.031 | 0.057 |
| F | 16.96 | 16.93 | 0.386 | 0.764 |
| I | 17.35 | 17.33 | 1.932 | 4.327 |
| K | 17.65 | 17.61 | 0.658 | 0.997 |
| L | 17.84 | 17.82 | 0.574 | 1.139 |

Wednesday, March 13, 2019 08:43:08

311LT-2 - Page 5 of 8

**RESIDUE #3 = I**

## Figure S5. A) continued

SequencePro™

Tufts Core Facility
Tufts Medical School
136 Harrison Ave
Boston, MA 02111

Cycle 6:Residue 4

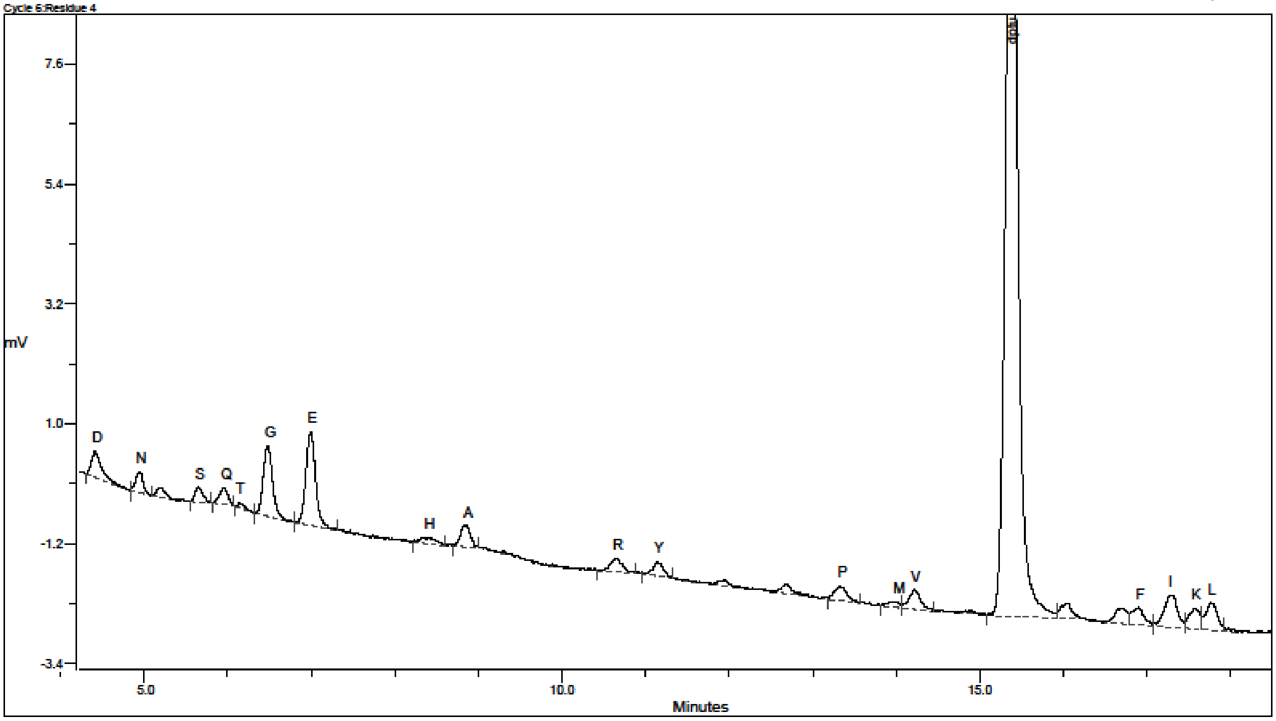


| PEAK ID | R.TIME (mins) | C.TIME (mins) | HEIGHT (mV) | PMOL HT | PEAK ID | R.TIME (mins) | C.TIME (mins) | HEIGHT (mV) | PMOL HT |
|---|---|---|---|---|---|---|---|---|---|
| D | 4.42 | 4.40 | 0.483 | 0.659 | Y | 11.14 | 11.15 | 0.254 | 0.553 |
| N | 4.94 | 4.91 | 0.387 | 0.577 | P | 13.34 | 13.34 | 0.270 | 0.670 |
| S | 5.65 | 5.62 | 0.272 | 0.617 | M | 14.00 | 14.00 | 0.113 | 0.233 |
| Q | 5.95 | 5.92 | 0.309 | 0.559 | V | 14.21 | 14.24 | 0.362 | 0.718 |
| T | 6.13 | 6.13 | 0.092 | 0.253 | dptu | 15.38 | 15.40 | 15.832 | 40.650 |
| G | 6.48 | 6.45 | 1.285 | 2.391 | F | 16.90 | 16.93 | 0.323 | 0.639 |
| E | 6.99 | 6.97 | 1.725 | 2.703 | I | 17.29 | 17.33 | 0.597 | 1.337 |
| H | 8.39 | 8.40 | 0.117 | 0.248 | K | 17.57 | 17.61 | 0.388 | 0.589 |
| A | 8.86 | 8.83 | 0.390 | 0.750 | L | 17.76 | 17.82 | 0.509 | 1.009 |
| R | 10.65 | 10.64 | 0.252 | 0.785 | | | | | |

Wednesday, March 13, 2019 08:43:08

311LT-2 - Page 6 of 8

**RESIDUE #4 = E**

## Figure S5. A) continued

SequencePro™

Tufts Core Facility
Tufts Medical School
136 Harrison Ave
Boston, MA 02111

Cycle 7:Residue 5

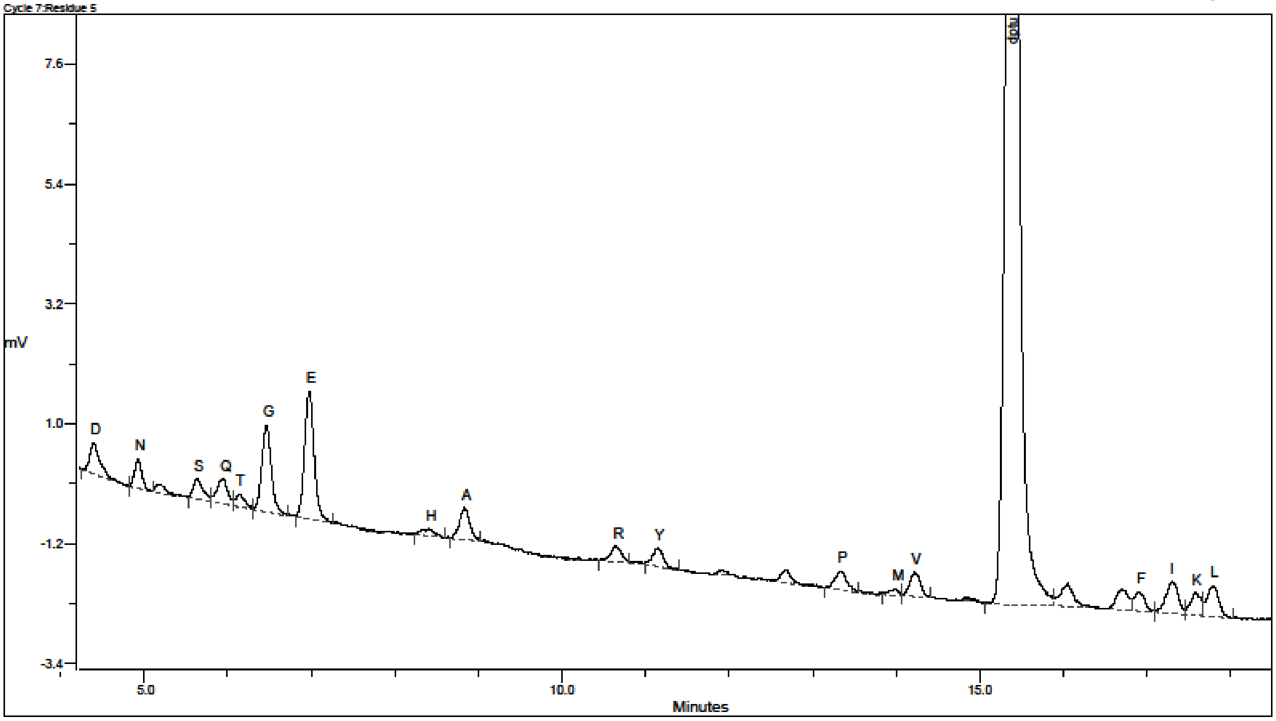


| PEAK ID | R.TIME (mins) | C.TIME (mins) | HEIGHT (mV) | PMOL HT |
|---|---|---|---|---|
| D | 4.40 | 4.40 | 0.566 | 0.771 |
| N | 4.92 | 4.91 | 0.540 | 0.806 |
| S | 5.63 | 5.62 | 0.369 | 0.837 |
| Q | 5.95 | 5.92 | 0.456 | 0.825 |
| T | 6.14 | 6.13 | 0.226 | 0.625 |
| G | 6.46 | 6.45 | 1.594 | 2.966 |
| E | 6.97 | 6.97 | 2.334 | 3.656 |
| H | 8.41 | 8.40 | 0.128 | 0.272 |
| A | 8.84 | 8.83 | 0.578 | 1.111 |
| R | 10.65 | 10.64 | 0.278 | 0.867 |

| PEAK ID | R.TIME (mins) | C.TIME (mins) | HEIGHT (mV) | PMOL HT |
|---|---|---|---|---|
| Y | 11.15 | 11.15 | 0.338 | 0.737 |
| P | 13.34 | 13.34 | 0.353 | 0.875 |
| M | 13.99 | 14.00 | 0.121 | 0.250 |
| V | 14.22 | 14.24 | 0.447 | 0.887 |
| dptu | 15.39 | 15.40 | 24.740 | 63.523 |
| F | 16.92 | 16.93 | 0.354 | 0.700 |
| I | 17.31 | 17.33 | 0.582 | 1.304 |
| K | 17.57 | 17.61 | 0.410 | 0.621 |
| L | 17.80 | 17.82 | 0.574 | 1.138 |

Wednesday, March 13, 2019 08:43:08

311LT-2 - Page 7 of 8

## RESIDUE #5 = E

## 5 cycles total

SequencePro™

Tufts Core Facility
Tufts Medical School
136 Harrison Ave
Boston, MA 02111

Raw Pmol Table

| | ASP | ASN | SER | GLN | THR | GLY | GLU | HIS | ALA | ARG | TYR | PRO | MET | VAL | TRP | PHE | ILE | LYS | LEU |
|---|---|---|---|---|---|---|---|---|---|---|---|---|---|---|---|---|---|---|---|
| 1 | 0.777 | 0.486 | 1.414 | 0.000 | 0.000 | 2.426 | 0.606 | 0.419 | 0.776 | 0.736 | 0.574 | 0.650 | 2.788 | 0.684 | 0.095 | 0.524 | 0.482 | 0.436 | 0.956 |
| 2 | 0.653 | 0.533 | 0.932 | 0.777 | 0.592 | 2.956 | 0.693 | 0.100 | 0.786 | 0.874 | 0.718 | 0.735 | 0.348 | 0.708 | 0.000 | 0.754 | 0.511 | 3.090 | 0.000 |
| 3 | 0.772 | 0.674 | 0.918 | 0.854 | 0.543 | 2.940 | 0.842 | 0.103 | 0.887 | 0.910 | 0.688 | 0.832 | 0.294 | 0.837 | 0.057 | 0.764 | 4.327 | 0.997 | 1.139 |
| 4 | 0.659 | 0.577 | 0.617 | 0.559 | 0.253 | 2.391 | 2.703 | 0.248 | 0.750 | 0.785 | 0.553 | 0.670 | 0.233 | 0.718 | 0.000 | 0.639 | 1.337 | 0.589 | 1.009 |
| 5 | 0.771 | 0.806 | 0.837 | 0.825 | 0.625 | 2.966 | 3.656 | 0.272 | 1.111 | 0.867 | 0.737 | 0.875 | 0.250 | 0.887 | 0.000 | 0.700 | 1.304 | 0.621 | 1.138 |

## Figure S5 B.

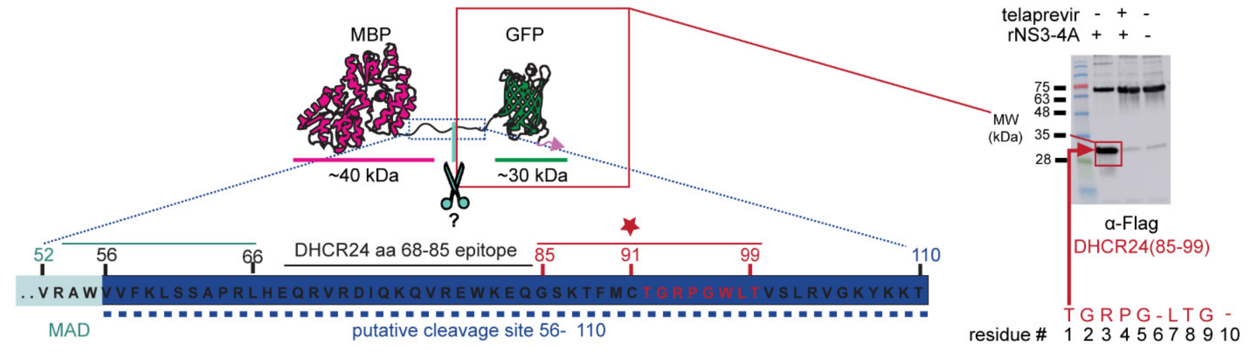


SequencePro™

Tufts Core Facility
Tufts Medical School
136 Harrison Ave
Boston, MA 02111

Cycle 2:Standard 1

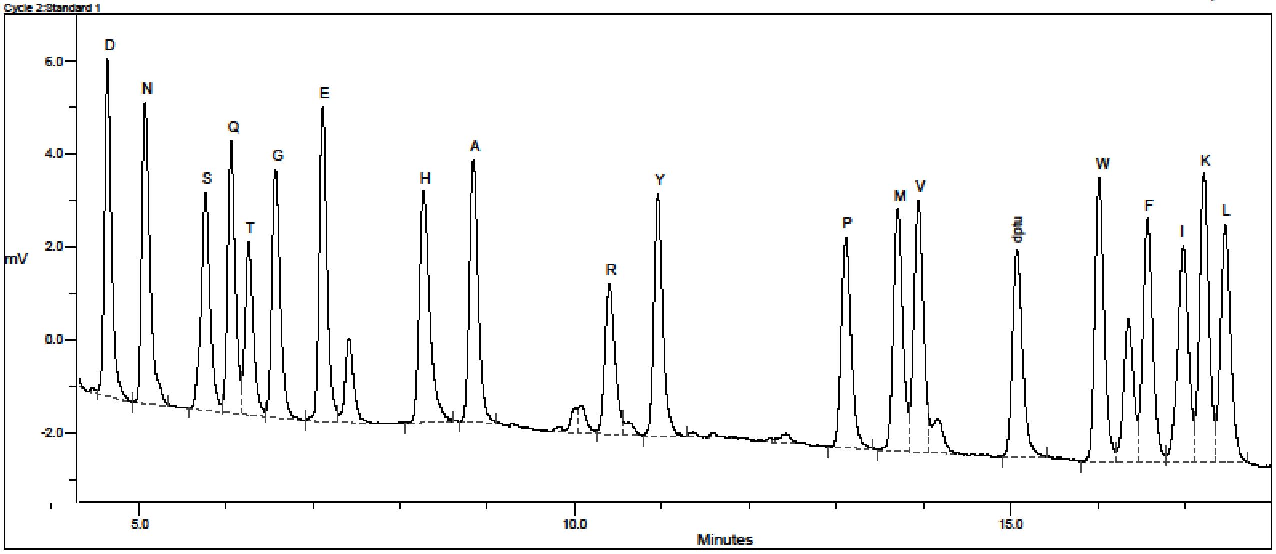


| PEAK ID | R.TIME (mins) | C.TIME (mins) | HEIGHT (mV) | PMOL HT |
|---|---|---|---|---|
| | 4.11 | | 14.413 | |
| | 4.48 | | 0.105 | |
| D | 4.65 | 4.65 | 7.261 | 10.000 |
| N | 5.08 | 5.08 | 6.499 | 10.000 |
| | 5.50 | | 0.029 | |
| S | 5.77 | 5.77 | 4.706 | 10.000 |
| Q | 6.06 | 6.06 | 5.866 | 10.000 |
| T | 6.26 | 6.26 | 3.726 | 10.000 |
| G | 6.57 | 6.57 | 5.347 | 10.000 |
| E | 7.11 | 7.11 | 6.781 | 10.000 |
| | 7.41 | | 1.827 | |
| | 7.74 | | 0.023 | |
| | 8.01 | | 0.040 | |
| H | 8.27 | 8.27 | 4.987 | 10.000 |
| A | 8.84 | 8.84 | 5.658 | 10.000 |
| | 9.82 | | 0.126 | |
| | 10.00 | | 0.547 | |
| | 10.07 | | 0.590 | |
| R | 10.40 | 10.40 | 3.240 | 10.000 |
| | 10.63 | | 0.184 | |
| Y | 10.96 | 10.96 | 5.216 | 10.000 |
| | 11.37 | | 0.095 | |
| | 11.60 | | 0.090 | |

| PEAK ID | R.TIME (mins) | C.TIME (mins) | HEIGHT (mV) | PMOL HT |
|---|---|---|---|---|
| | 11.80 | | 0.041 | |
| | 12.25 | | 0.084 | |
| | 12.43 | | 0.191 | |
| | 12.78 | | 0.037 | |
| P | 13.11 | 13.11 | 4.552 | 10.000 |
| M | 13.70 | 13.70 | 5.221 | 10.000 |
| V | 13.95 | 13.95 | 5.438 | 10.000 |
| | 14.16 | | 0.574 | |
| | 14.68 | | 0.029 | |
| | 14.86 | | 0.028 | |
| dptu | 15.07 | 15.07 | 4.453 | 10.000 |
| W | 16.02 | 16.02 | 6.128 | 10.000 |
| | 16.35 | | 3.079 | |
| F | 16.57 | 16.57 | 5.242 | 10.000 |
| I | 16.98 | 16.98 | 4.676 | 10.000 |
| K | 17.22 | 17.22 | 6.224 | 10.000 |
| L | 17.46 | 17.46 | 5.137 | 10.000 |
| | 18.02 | | 0.045 | |
| | 18.15 | | 0.044 | |
| | 18.44 | | 0.042 | |
| | 18.54 | | 0.047 | |
| | 18.83 | | 0.039 | |

Wednesday, March 13, 2019 08:45:43

311LT-3 - Page 2 of 13

**STANDARD**

## Figure S5. B) continued

SequencePro™

Tufts Core Facility
Tufts Medical School
136 Harrison Ave
Boston, MA 02111

Cycle 3:Residue 1

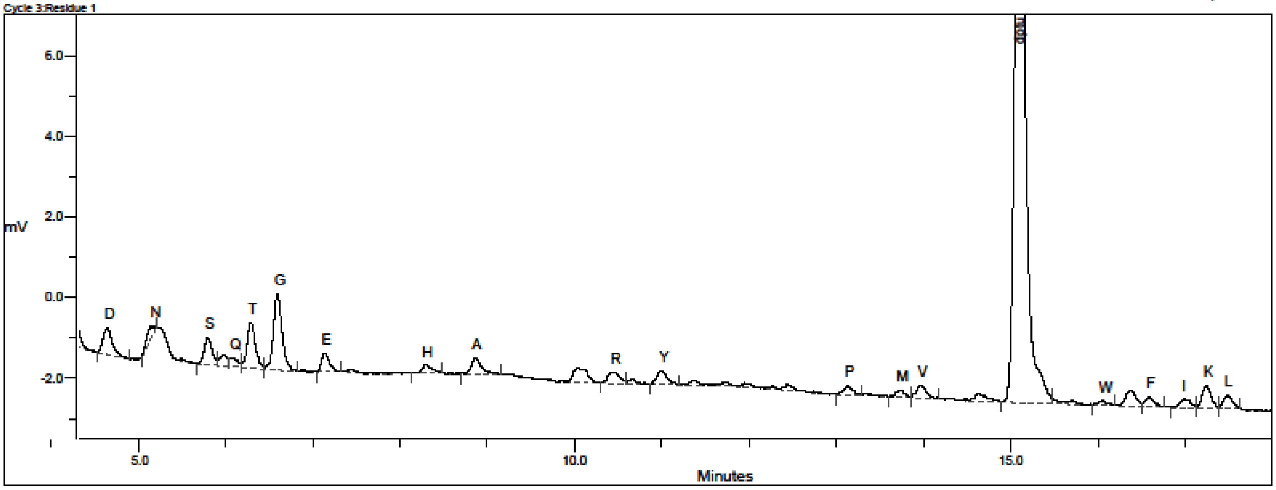


| PEAK ID | R.TIME (mins) | C.TIME (mins) | HEIGHT (mV) | PMOL HT |
|---|---|---|---|---|
| | 4.13 | | 14.525 | |
| D | 4.65 | 4.65 | 0.666 | 0.917 |
| N | 5.17 | 5.08 | 0.189 | 0.292 |
| | 5.50 | | 0.061 | |
| S | 5.80 | 5.77 | 0.693 | 1.472 |
| | 5.97 | | 0.272 | |
| Q | 6.08 | 6.06 | 0.224 | 0.383 |
| T | 6.29 | 6.26 | 1.130 | 3.034 |
| G | 6.59 | 6.57 | 1.888 | 3.531 |
| | 6.86 | | 0.045 | |
| | 7.02 | | 0.023 | |
| E | 7.14 | 7.11 | 0.470 | 0.694 |
| | 7.46 | | 0.083 | |
| | 7.80 | | 0.031 | |
| | 7.91 | | 0.023 | |
| H | 8.29 | 8.27 | 0.200 | 0.400 |
| | 8.57 | | 0.032 | |
| | 8.68 | | 0.021 | |
| A | 8.86 | 8.84 | 0.418 | 0.739 |
| | 9.16 | | 0.036 | |
| | 9.62 | | 0.033 | |
| | 9.85 | | 0.040 | |
| | 10.03 | | 0.368 | |
| R | 10.45 | 10.40 | 0.283 | 0.874 |
| | 10.66 | | 0.115 | |
| | 10.80 | | 0.037 | |
| Y | 11.01 | 10.96 | 0.332 | 0.637 |
| | 11.25 | | 0.033 | |

| PEAK ID | R.TIME (mins) | C.TIME (mins) | HEIGHT (mV) | PMOL HT |
|---|---|---|---|---|
| | 11.37 | | 0.118 | |
| | 11.73 | | 0.091 | |
| | 11.95 | | 0.098 | |
| | 12.28 | | 0.068 | |
| | 12.44 | | 0.132 | |
| | 12.74 | | 0.047 | |
| P | 13.13 | 13.11 | 0.227 | 0.500 |
| | 13.55 | | 0.046 | |
| M | 13.73 | 13.70 | 0.168 | 0.322 |
| V | 13.97 | 13.95 | 0.315 | 0.579 |
| | 14.23 | | 0.031 | |
| | 14.29 | | 0.023 | |
| | 14.48 | | 0.058 | |
| | 14.64 | | 0.194 | |
| dptu | 15.10 | 15.07 | 16.794 | 37.709 |
| | 15.70 | | 0.081 | |
| W | 16.05 | 16.02 | 0.113 | 0.185 |
| | 16.37 | | 0.403 | |
| F | 16.59 | 16.57 | 0.237 | 0.452 |
| I | 17.00 | 16.98 | 0.219 | 0.470 |
| K | 17.25 | 17.22 | 0.581 | 0.934 |
| L | 17.49 | 17.46 | 0.331 | 0.645 |
| | 17.75 | | 0.044 | |
| | 17.84 | | 0.058 | |
| | 18.00 | | 0.042 | |
| | 18.45 | | 0.029 | |
| | 18.60 | | 0.030 | |

Wednesday, March 13, 2019 08:45:43

311LT-3 - Page 3 of 13

## RESIDUE #1 = T

## Figure S5. B) continued

SequencePro™

Tufts Core Facility
Tufts Medical School
136 Harrison Ave
Boston, MA 02111

Cycle 4:Residue 2

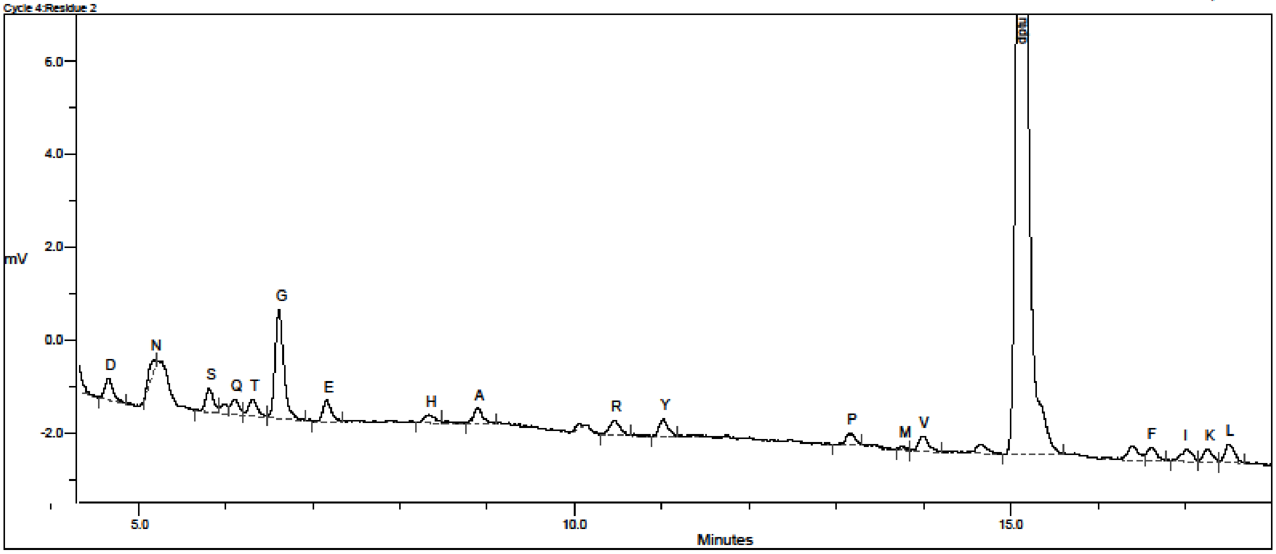


| PEAK ID | R.TIME (mins) | C.TIME (mins) | HEIGHT (mV) | PMOL HT |
|---|---|---|---|---|
| | 4.15 | | 14.125 | |
| D | 4.66 | 4.65 | 0.461 | 0.635 |
| N | 5.18 | 5.08 | 0.251 | 0.386 |
| S | 5.82 | 5.77 | 0.523 | 1.112 |
| | 6.00 | | 0.201 | |
| Q | 6.10 | 6.06 | 0.322 | 0.548 |
| T | 6.32 | 6.26 | 0.370 | 0.994 |
| G | 6.61 | 6.57 | 2.356 | 4.407 |
| | 6.95 | | 0.039 | |
| E | 7.17 | 7.11 | 0.461 | 0.679 |
| | 7.85 | | 0.028 | |
| | 7.95 | | 0.024 | |
| H | 8.33 | 8.27 | 0.184 | 0.370 |
| | 8.62 | | 0.036 | |
| | 8.72 | | 0.039 | |
| A | 8.89 | 8.84 | 0.342 | 0.604 |
| | 9.13 | | 0.044 | |
| | 9.42 | | 0.039 | |
| | 9.74 | | 0.027 | |
| | 10.07 | | 0.085 | |
| R | 10.46 | 10.40 | 0.317 | 0.978 |
| | 10.68 | | 0.038 | |
| | 10.83 | | 0.034 | |

| PEAK ID | R.TIME (mins) | C.TIME (mins) | HEIGHT (mV) | PMOL HT |
|---|---|---|---|---|
| Y | 11.02 | 10.96 | 0.376 | 0.720 |
| | 11.22 | | 0.032 | |
| | 11.42 | | 0.044 | |
| | 12.16 | | 0.034 | |
| | 12.49 | | 0.047 | |
| | 12.89 | | 0.028 | |
| P | 13.16 | 13.11 | 0.249 | 0.547 |
| M | 13.75 | 13.70 | 0.089 | 0.170 |
| V | 13.99 | 13.95 | 0.332 | 0.610 |
| | 14.30 | | 0.048 | |
| | 14.67 | | 0.197 | |
| dptu | 15.13 | 15.07 | 21.124 | 47.434 |
| | 16.15 | | 0.049 | |
| | 16.23 | | 0.035 | |
| | 16.39 | | 0.313 | |
| F | 16.60 | 16.57 | 0.281 | 0.536 |
| I | 17.02 | 16.98 | 0.278 | 0.595 |
| K | 17.27 | 17.22 | 0.279 | 0.449 |
| L | 17.50 | 17.46 | 0.398 | 0.775 |
| | 18.06 | | 0.017 | |
| | 18.30 | | 0.021 | |
| | 18.64 | | 0.037 | |
| | 18.70 | | 0.042 | |

Wednesday, March 13, 2019 08:45:44

311LT-3 - Page 4 of 13

**RESIDUE #2 = G**

## Figure S5. B) continued

SequencePro™

Tufts Core Facility
Tufts Medical School
136 Harrison Ave
Boston, MA 02111

Cycle 5:Residue 3

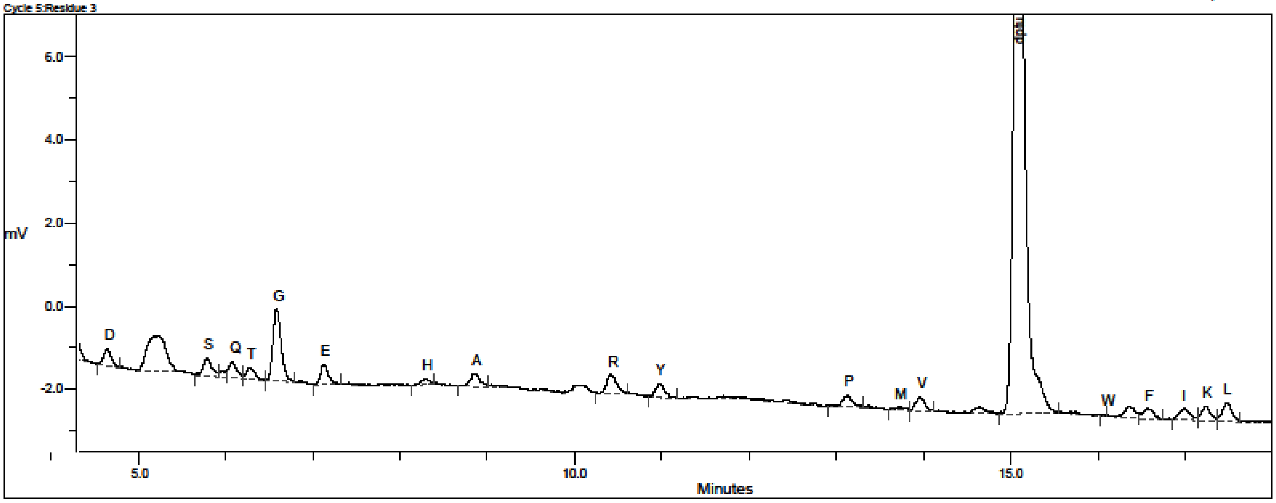


| PEAK ID | R.TIME (mins) | C.TIME (mins) | HEIGHT (mV) | PMOL HT |
|---|---|---|---|---|
| | 4.13 | | 14.342 | |
| D | 4.65 | 4.65 | 0.409 | 0.564 |
| | 4.92 | | 0.029 | |
| | 5.22 | | 0.846 | |
| S | 5.78 | 5.77 | 0.442 | 0.939 |
| | 5.97 | | 0.156 | |
| Q | 6.09 | 6.06 | 0.393 | 0.670 |
| T | 6.28 | 6.26 | 0.286 | 0.769 |
| G | 6.58 | 6.57 | 1.732 | 3.239 |
| E | 7.13 | 7.11 | 0.489 | 0.721 |
| | 7.42 | | 0.042 | |
| | 7.80 | | 0.041 | |
| | 7.92 | | 0.044 | |
| | 8.08 | | 0.025 | |
| H | 8.29 | 8.27 | 0.134 | 0.268 |
| | 8.57 | | 0.035 | |
| A | 8.86 | 8.84 | 0.307 | 0.543 |
| | 9.05 | | 0.041 | |
| | 9.21 | | 0.042 | |
| | 9.61 | | 0.043 | |
| | 9.89 | | 0.042 | |
| | 10.01 | | 0.058 | |
| R | 10.42 | 10.40 | 0.451 | 1.392 |
| Y | 10.97 | 10.96 | 0.320 | 0.613 |
| | 11.31 | | 0.028 | |
| | 11.71 | | 0.060 | |
| | 12.11 | | 0.059 | |

| PEAK ID | R.TIME (mins) | C.TIME (mins) | HEIGHT (mV) | PMOL HT |
|---|---|---|---|---|
| | 12.28 | | 0.052 | |
| | 12.41 | | 0.057 | |
| | 12.61 | | 0.037 | |
| | 12.73 | | 0.039 | |
| P | 13.13 | 13.11 | 0.281 | 0.618 |
| | 13.37 | | 0.072 | |
| M | 13.71 | 13.70 | 0.075 | 0.144 |
| V | 13.96 | 13.95 | 0.350 | 0.644 |
| | 14.34 | | 0.033 | |
| | 14.48 | | 0.032 | |
| | 14.64 | | 0.147 | |
| dptu | 15.09 | 15.07 | 17.097 | 38.390 |
| | 15.70 | | 0.049 | |
| | 15.98 | | 0.023 | |
| W | 16.08 | 16.02 | 0.028 | 0.045 |
| | 16.36 | | 0.275 | |
| F | 16.57 | 16.57 | 0.255 | 0.486 |
| | 16.77 | | 0.030 | |
| I | 17.00 | 16.98 | 0.268 | 0.573 |
| K | 17.23 | 17.22 | 0.343 | 0.551 |
| L | 17.47 | 17.46 | 0.440 | 0.857 |
| | 17.68 | | 0.038 | |
| | 17.75 | | 0.028 | |
| | 17.84 | | 0.028 | |
| | 18.27 | | 0.028 | |
| | 18.60 | | 0.040 | |

Wednesday, March 13, 2019 08:45:44

311LT-3 - Page 5 of 13

## RESIDUE #3 = R

## Figure S5. B) continued

SequencePro™

Tufts Core Facility
Tufts Medical School
136 Harrison Ave
Boston, MA 02111

Cycle 6:Residue 4

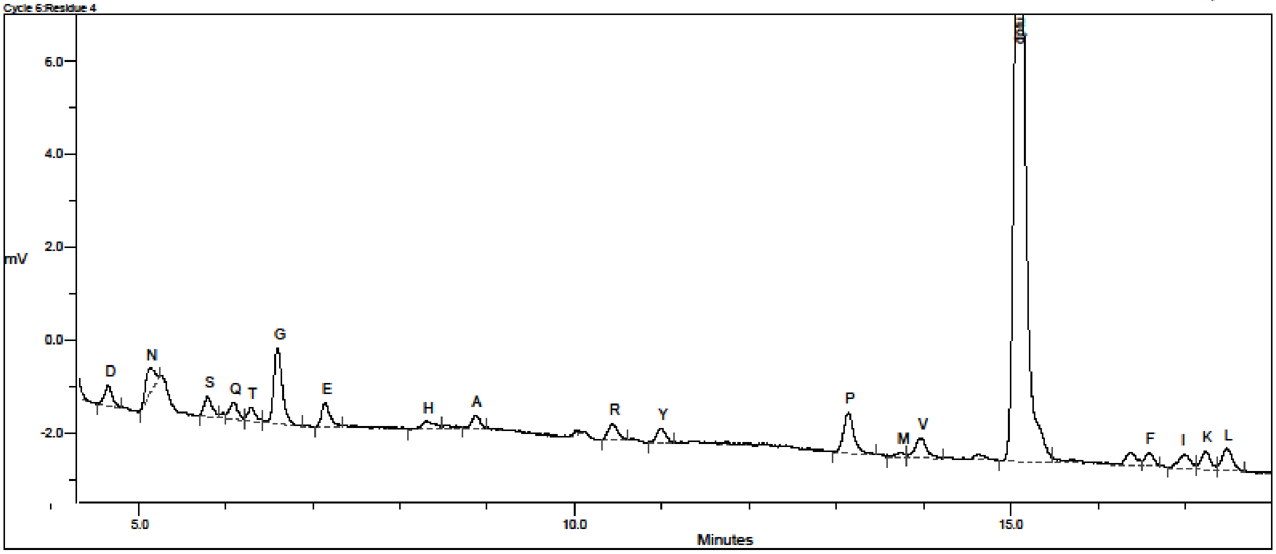


| PEAK ID | R.TIME (mins) | C.TIME (mins) | HEIGHT (mV) | PMOL HT |
|---|---|---|---|---|
| | 4.13 | | 13.724 | |
| D | 4.66 | 4.65 | 0.441 | 0.608 |
| N | 5.13 | 5.08 | 0.567 | 0.872 |
| S | 5.80 | 5.77 | 0.445 | 0.945 |
| | 5.97 | | 0.130 | |
| Q | 6.09 | 6.06 | 0.361 | 0.615 |
| T | 6.29 | 6.26 | 0.285 | 0.764 |
| G | 6.60 | 6.57 | 1.643 | 3.073 |
| | 6.90 | | 0.038 | |
| E | 7.14 | 7.11 | 0.525 | 0.774 |
| | 7.45 | | 0.029 | |
| | 7.85 | | 0.029 | |
| | 8.03 | | 0.048 | |
| H | 8.30 | 8.27 | 0.178 | 0.358 |
| | 8.54 | | 0.078 | |
| | 8.64 | | 0.058 | |
| A | 8.86 | 8.84 | 0.292 | 0.516 |
| | 9.16 | | 0.052 | |
| | 9.68 | | 0.032 | |
| | 10.02 | | 0.106 | |
| R | 10.44 | 10.40 | 0.358 | 1.105 |
| Y | 11.00 | 10.96 | 0.308 | 0.590 |
| | 11.23 | | 0.030 | |

| PEAK ID | R.TIME (mins) | C.TIME (mins) | HEIGHT (mV) | PMOL HT |
|---|---|---|---|---|
| | 11.33 | | 0.023 | |
| | 11.74 | | 0.057 | |
| | 11.89 | | 0.032 | |
| | 11.96 | | 0.047 | |
| | 12.07 | | 0.042 | |
| P | 13.14 | 13.11 | 0.878 | 1.930 |
| M | 13.74 | 13.70 | 0.109 | 0.210 |
| V | 13.98 | 13.95 | 0.432 | 0.794 |
| | 14.45 | | 0.041 | |
| | 14.63 | | 0.128 | |
| dptu | 15.10 | 15.07 | 15.947 | 35.807 |
| | 15.73 | | 0.078 | |
| | 16.20 | | 0.037 | |
| | 16.37 | | 0.290 | |
| F | 16.58 | 16.57 | 0.282 | 0.538 |
| I | 16.99 | 16.98 | 0.304 | 0.651 |
| K | 17.23 | 17.22 | 0.398 | 0.639 |
| L | 17.48 | 17.46 | 0.478 | 0.930 |
| | 17.92 | | 0.030 | |
| | 18.14 | | 0.039 | |
| | 18.35 | | 0.027 | |
| | 18.43 | | 0.042 | |

Wednesday, March 13, 2019 08:45:44

311LT-3 - Page 6 of 13

**RESIDUE #4 = P**

## Figure S5. B) continued

SequencePro™

Tufts Core Facility
Tufts Medical School
136 Harrison Ave
Boston, MA 02111

Cycle 7:Residue 5

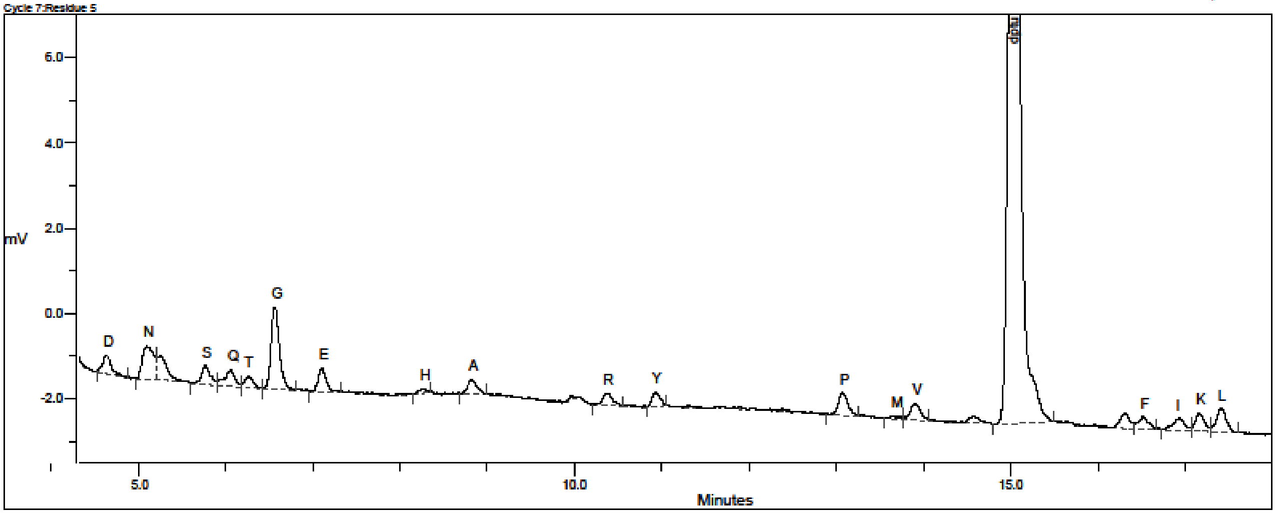


| PEAK ID | R.TIME (mins) | C.TIME (mins) | HEIGHT (mV) | PMOL HT |
|---|---|---|---|---|
| | 4.11 | | 13.317 | |
| D | 4.63 | 4.65 | 0.449 | 0.618 |
| N | 5.10 | 5.08 | 0.778 | 1.198 |
| | 5.25 | | 0.572 | |
| S | 5.77 | 5.77 | 0.447 | 0.951 |
| Q | 6.06 | 6.06 | 0.385 | 0.657 |
| T | 6.25 | 6.26 | 0.265 | 0.710 |
| G | 6.56 | 6.57 | 1.937 | 3.623 |
| E | 7.11 | 7.11 | 0.558 | 0.822 |
| | 7.42 | | 0.036 | |
| | 7.55 | | 0.046 | |
| | 7.75 | | 0.021 | |
| | 8.03 | | 0.039 | |
| | 8.11 | | 0.028 | |
| H | 8.27 | 8.27 | 0.105 | 0.210 |
| | 8.52 | | 0.033 | |
| | 8.68 | | 0.037 | |
| A | 8.82 | 8.84 | 0.351 | 0.621 |
| | 9.24 | | 0.043 | |
| | 9.48 | | 0.032 | |
| | 9.96 | | 0.083 | |
| R | 10.37 | 10.40 | 0.286 | 0.883 |
| | 10.60 | | 0.040 | |
| | 10.80 | | 0.044 | |
| Y | 10.93 | 10.96 | 0.328 | 0.630 |
| | 11.15 | | 0.030 | |

| PEAK ID | R.TIME (mins) | C.TIME (mins) | HEIGHT (mV) | PMOL HT |
|---|---|---|---|---|
| | 11.30 | | 0.052 | |
| | 11.64 | | 0.026 | |
| | 11.83 | | 0.040 | |
| | 11.90 | | 0.053 | |
| | 12.24 | | 0.031 | |
| | 12.37 | | 0.054 | |
| P | 13.07 | 13.11 | 0.536 | 1.177 |
| | 13.30 | | 0.026 | |
| M | 13.67 | 13.70 | 0.075 | 0.144 |
| V | 13.91 | 13.95 | 0.390 | 0.718 |
| | 14.10 | | 0.035 | |
| | 14.31 | | 0.041 | |
| | 14.57 | | 0.151 | |
| dptu | 15.03 | 15.07 | 22.251 | 49.964 |
| | 15.58 | | 0.020 | |
| | 15.76 | | 0.028 | |
| | 16.31 | | 0.353 | |
| F | 16.52 | 16.57 | 0.286 | 0.546 |
| | 16.70 | | 0.044 | |
| I | 16.93 | 16.98 | 0.285 | 0.609 |
| K | 17.16 | 17.22 | 0.419 | 0.674 |
| L | 17.41 | 17.46 | 0.543 | 1.056 |
| | 18.10 | | 0.030 | |
| | 18.25 | | 0.038 | |
| | 18.39 | | 0.040 | |
| | 18.72 | | 0.035 | |

Wednesday, March 13, 2019 08:45:44

311LT-3 - Page 7 of 13

## RESIDUE #5 = G

## Figure S5. B) continued

SequencePro™

Tufts Core Facility
Tufts Medical School
136 Harrison Ave
Boston, MA 02111

Cycle 8:Residue 6

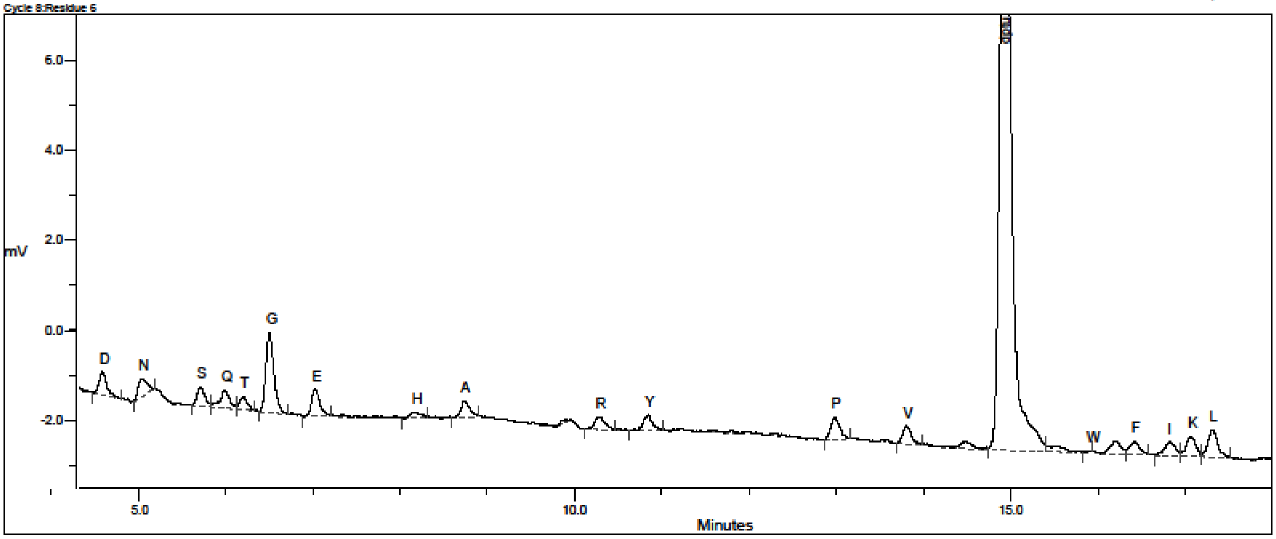


| PEAK ID | R.TIME (mins) | C.TIME (mins) | HEIGHT (mV) | PMOL HT |
|---|---|---|---|---|
| | 4.08 | | 12.434 | |
| D | 4.59 | 4.65 | 0.514 | 0.708 |
| N | 5.04 | 5.08 | 0.410 | 0.631 |
| S | 5.70 | 5.77 | 0.431 | 0.917 |
| Q | 5.99 | 6.06 | 0.398 | 0.678 |
| T | 6.20 | 6.26 | 0.283 | 0.760 |
| G | 6.50 | 6.57 | 1.768 | 3.307 |
| | 6.75 | | 0.038 | |
| E | 7.03 | 7.11 | 0.604 | 0.891 |
| | 7.33 | | 0.044 | |
| | 7.53 | | 0.042 | |
| | 7.58 | | 0.049 | |
| | 7.74 | | 0.039 | |
| | 7.93 | | 0.036 | |
| H | 8.17 | 8.27 | 0.128 | 0.256 |
| | 8.51 | | 0.033 | |
| | 8.57 | | 0.034 | |
| A | 8.73 | 8.84 | 0.353 | 0.623 |
| | 9.75 | | 0.023 | |
| | 9.91 | | 0.037 | |
| R | 10.28 | 10.40 | 0.282 | 0.871 |
| | 10.50 | | 0.040 | |
| | 10.58 | | 0.029 | |
| Y | 10.85 | 10.96 | 0.342 | 0.657 |

| PEAK ID | R.TIME (mins) | C.TIME (mins) | HEIGHT (mV) | PMOL HT |
|---|---|---|---|---|
| | 11.13 | | 0.035 | |
| | 11.58 | | 0.047 | |
| | 11.80 | | 0.064 | |
| | 12.29 | | 0.062 | |
| P | 12.98 | 13.11 | 0.494 | 1.085 |
| V | 13.80 | 13.95 | 0.405 | 0.745 |
| | 14.25 | | 0.038 | |
| | 14.49 | | 0.151 | |
| | 14.72 | | 0.056 | |
| dptu | 14.93 | 15.07 | 17.263 | 38.764 |
| | 15.53 | | 0.104 | |
| W | 15.91 | 16.02 | 0.021 | 0.034 |
| | 16.20 | | 0.292 | |
| F | 16.42 | 16.57 | 0.282 | 0.538 |
| I | 16.83 | 16.98 | 0.304 | 0.651 |
| K | 17.07 | 17.22 | 0.447 | 0.718 |
| L | 17.31 | 17.46 | 0.599 | 1.166 |
| | 17.59 | | 0.038 | |
| | 17.78 | | 0.036 | |
| | 17.95 | | 0.029 | |
| | 18.22 | | 0.031 | |
| | 18.34 | | 0.032 | |
| | 18.75 | | 0.034 | |
| | 18.88 | | 0.043 | |

Wednesday, March 13, 2019 08:45:44

311LT-3 - Page 8 of 13

**RESIDUE #6 = -**

## Figure S5. B) continued

SequencePro™

Tufts Core Facility
Tufts Medical School
136 Harrison Ave
Boston, MA 02111

Cycle 9:Residue 7

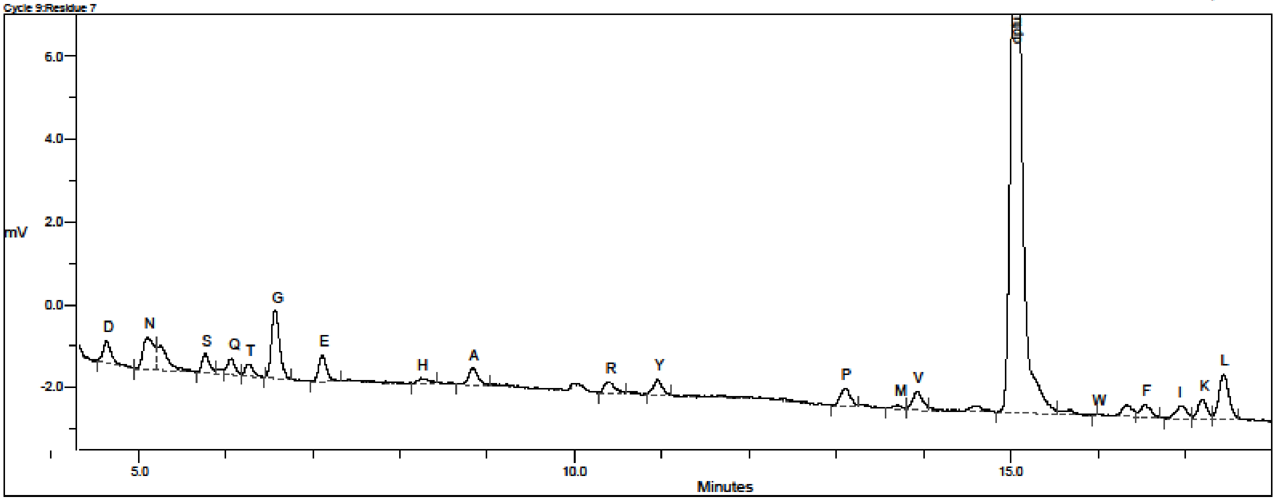


| PEAK ID | R.TIME (mins) | C.TIME (mins) | HEIGHT (mV) | PMOL HT |
|---|---|---|---|---|
| | 4.12 | | 13.677 | |
| D | 4.63 | 4.65 | 0.540 | 0.744 |
| N | 5.11 | 5.08 | 0.769 | 1.183 |
| | 5.26 | | 0.598 | |
| S | 5.77 | 5.77 | 0.453 | 0.964 |
| | 5.95 | | 0.123 | |
| Q | 6.07 | 6.06 | 0.395 | 0.674 |
| T | 6.27 | 6.26 | 0.300 | 0.804 |
| G | 6.57 | 6.57 | 1.638 | 3.064 |
| | 6.92 | | 0.031 | |
| E | 7.11 | 7.11 | 0.655 | 0.967 |
| | 7.42 | | 0.060 | |
| | 7.61 | | 0.033 | |
| | 7.83 | | 0.050 | |
| H | 8.24 | 8.27 | 0.134 | 0.269 |
| | 8.58 | | 0.043 | |
| A | 8.83 | 8.84 | 0.402 | 0.711 |
| | 9.05 | | 0.025 | |
| | 9.22 | | 0.042 | |
| | 9.82 | | 0.039 | |
| | 9.99 | | 0.063 | |
| R | 10.40 | 10.40 | 0.270 | 0.833 |
| | 10.80 | | 0.043 | |
| Y | 10.95 | 10.96 | 0.370 | 0.710 |
| | 11.35 | | 0.069 | |
| | 11.57 | | 0.033 | |
| | 11.69 | | 0.055 | |

| PEAK ID | R.TIME (mins) | C.TIME (mins) | HEIGHT (mV) | PMOL HT |
|---|---|---|---|---|
| | 11.88 | | 0.043 | |
| | 12.12 | | 0.034 | |
| | 12.35 | | 0.044 | |
| | 12.42 | | 0.062 | |
| | 12.64 | | 0.038 | |
| | 12.80 | | 0.040 | |
| P | 13.10 | 13.11 | 0.417 | 0.915 |
| M | 13.71 | 13.70 | 0.098 | 0.189 |
| V | 13.93 | 13.95 | 0.458 | 0.843 |
| | 14.20 | | 0.044 | |
| | 14.28 | | 0.044 | |
| | 14.34 | | 0.032 | |
| | 14.61 | | 0.125 | |
| dptu | 15.06 | 15.07 | 15.700 | 35.253 |
| | 15.68 | | 0.102 | |
| | 15.91 | | 0.019 | |
| W | 15.98 | 16.02 | 0.024 | 0.039 |
| | 16.34 | | 0.280 | |
| F | 16.54 | 16.57 | 0.311 | 0.593 |
| I | 16.95 | 16.98 | 0.308 | 0.659 |
| K | 17.20 | 17.22 | 0.471 | 0.757 |
| L | 17.44 | 17.46 | 1.063 | 2.069 |
| | 17.90 | | 0.046 | |
| | 18.09 | | 0.027 | |
| | 18.28 | | 0.035 | |
| | 18.48 | | 0.022 | |
| | 18.68 | | 0.029 | |

Wednesday, March 13, 2019 08:45:44

311LT-3 - Page 9 of 13

## RESIDUE #7 = L

## Figure S5. B) continued

SequencePro™

Tufts Core Facility
Tufts Medical School
136 Harrison Ave
Boston, MA 02111

Cycle 10:Residue 8

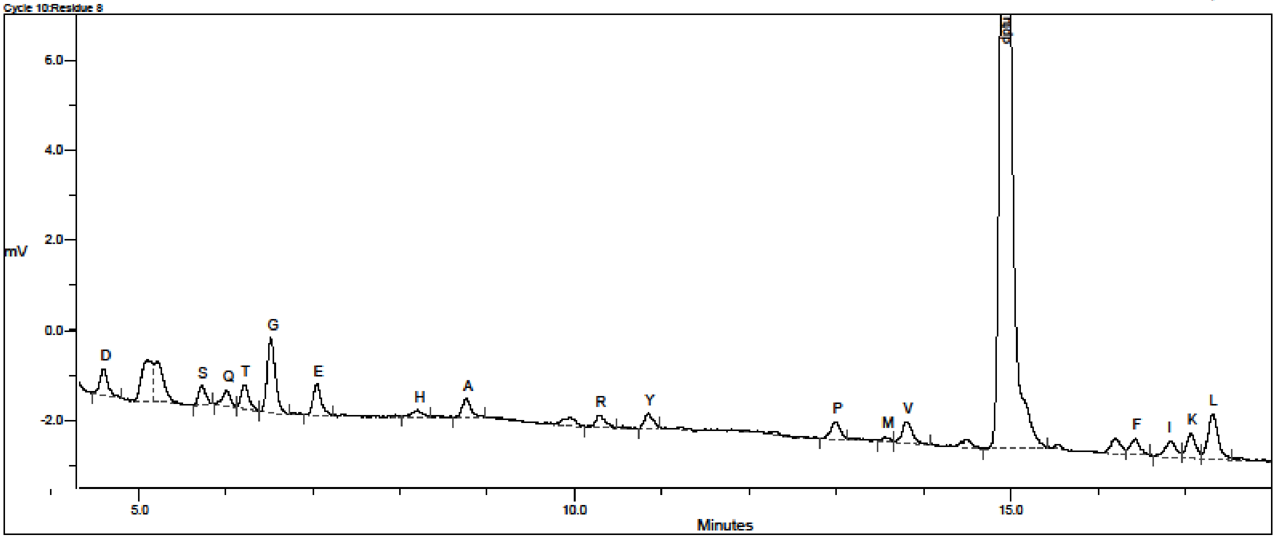


| PEAK ID | R.TIME (mins) | C.TIME (mins) | HEIGHT (mV) | PMOL HT |
|---|---|---|---|---|
| | 4.09 | | 14.082 | |
| D | 4.60 | 4.65 | 0.567 | 0.781 |
| | 5.10 | | 0.912 | |
| | 5.21 | | 0.895 | |
| S | 5.72 | 5.77 | 0.422 | 0.896 |
| Q | 6.01 | 6.06 | 0.363 | 0.619 |
| T | 6.22 | 6.26 | 0.516 | 1.386 |
| G | 6.52 | 6.57 | 1.656 | 3.098 |
| | 6.78 | | 0.043 | |
| E | 7.05 | 7.11 | 0.686 | 1.012 |
| | 7.34 | | 0.046 | |
| | 7.81 | | 0.047 | |
| | 7.95 | | 0.047 | |
| H | 8.20 | 8.27 | 0.150 | 0.302 |
| | 8.57 | | 0.044 | |
| A | 8.76 | 8.84 | 0.426 | 0.753 |
| | 9.94 | | 0.182 | |
| R | 10.28 | 10.40 | 0.259 | 0.799 |
| | 10.55 | | 0.059 | |
| | 10.63 | | 0.027 | |
| Y | 10.84 | 10.96 | 0.333 | 0.638 |
| | 11.03 | | 0.028 | |
| | 11.19 | | 0.050 | |
| | 11.50 | | 0.032 | |

| PEAK ID | R.TIME (mins) | C.TIME (mins) | HEIGHT (mV) | PMOL HT |
|---|---|---|---|---|
| | 11.95 | | 0.050 | |
| | 12.29 | | 0.076 | |
| | 12.60 | | 0.043 | |
| | 12.77 | | 0.039 | |
| P | 13.00 | 13.11 | 0.390 | 0.858 |
| | 13.20 | | 0.038 | |
| M | 13.56 | 13.70 | 0.108 | 0.207 |
| V | 13.81 | 13.95 | 0.480 | 0.882 |
| | 14.26 | | 0.028 | |
| | 14.49 | | 0.180 | |
| dptu | 14.94 | 15.07 | 19.670 | 44.169 |
| | 15.56 | | 0.091 | |
| | 16.20 | | 0.328 | |
| F | 16.43 | 16.57 | 0.352 | 0.672 |
| I | 16.83 | 16.98 | 0.354 | 0.757 |
| K | 17.06 | 17.22 | 0.551 | 0.885 |
| L | 17.32 | 17.46 | 1.003 | 1.952 |
| | 17.65 | | 0.067 | |
| | 17.82 | | 0.044 | |
| | 18.03 | | 0.031 | |
| | 18.11 | | 0.034 | |
| | 18.49 | | 0.050 | |
| | 18.59 | | 0.046 | |
| | 18.75 | | 0.030 | |

Wednesday, March 13, 2019 08:45:45

311LT-3 - Page 10 of 13

## RESIDUE #8 = T

## Figure S5. B) continued

SequencePro™

Tufts Core Facility
Tufts Medical School
136 Harrison Ave
Boston, MA 02111

Cycle 11:Residue 9

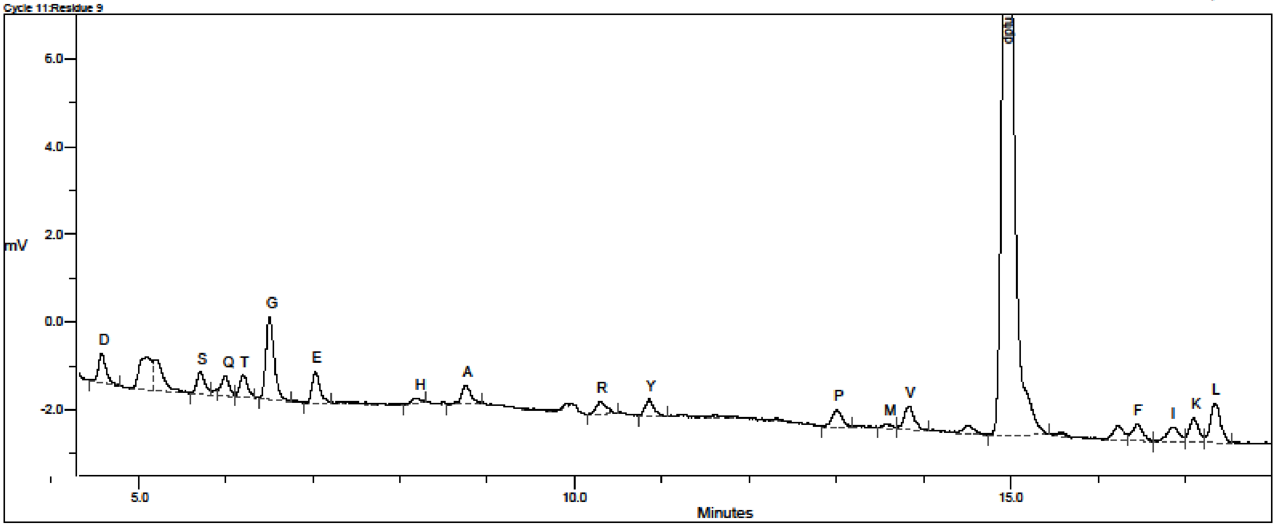


| PEAK ID | R.TIME (mins) | C.TIME (mins) | HEIGHT (mV) | PMOL HT |
|---|---|---|---|---|
| | 4.07 | | 14.041 | |
| D | 4.58 | 4.65 | 0.668 | 0.919 |
| | 5.09 | | 0.762 | |
| | 5.19 | | 0.695 | |
| S | 5.71 | 5.77 | 0.507 | 1.077 |
| | 5.90 | | 0.142 | |
| Q | 6.00 | 6.06 | 0.449 | 0.766 |
| T | 6.20 | 6.26 | 0.506 | 1.358 |
| G | 6.50 | 6.57 | 1.884 | 3.524 |
| E | 7.03 | 7.11 | 0.717 | 1.057 |
| | 7.35 | | 0.037 | |
| | 7.61 | | 0.047 | |
| | 7.71 | | 0.044 | |
| | 8.02 | | 0.045 | |
| H | 8.21 | 8.27 | 0.111 | 0.223 |
| | 8.50 | | 0.046 | |
| A | 8.75 | 8.84 | 0.424 | 0.749 |
| | 9.04 | | 0.021 | |
| | 9.41 | | 0.026 | |
| | 9.54 | | 0.030 | |
| | 9.72 | | 0.026 | |
| | 9.91 | | 0.070 | |
| R | 10.29 | 10.40 | 0.283 | 0.875 |
| Y | 10.86 | 10.96 | 0.385 | 0.739 |
| | 11.11 | | 0.030 | |

| PEAK ID | R.TIME (mins) | C.TIME (mins) | HEIGHT (mV) | PMOL HT |
|---|---|---|---|---|
| | 11.52 | | 0.036 | |
| | 11.62 | | 0.052 | |
| | 11.84 | | 0.042 | |
| | 11.98 | | 0.025 | |
| | 12.31 | | 0.065 | |
| P | 13.01 | 13.11 | 0.391 | 0.859 |
| | 13.27 | | 0.033 | |
| M | 13.59 | 13.70 | 0.104 | 0.199 |
| V | 13.83 | 13.95 | 0.538 | 0.989 |
| | 14.30 | | 0.038 | |
| | 14.51 | | 0.180 | |
| dptu | 14.96 | 15.07 | 19.453 | 43.680 |
| | 15.58 | | 0.100 | |
| | 16.23 | | 0.336 | |
| F | 16.44 | 16.57 | 0.388 | 0.741 |
| I | 16.87 | 16.98 | 0.346 | 0.740 |
| K | 17.10 | 17.22 | 0.551 | 0.886 |
| L | 17.35 | 17.46 | 0.912 | 1.775 |
| | 17.73 | | 0.049 | |
| | 17.86 | | 0.030 | |
| | 18.07 | | 0.035 | |
| | 18.23 | | 0.039 | |
| | 18.35 | | 0.035 | |
| | 18.60 | | 0.036 | |
| | 18.89 | | 0.035 | |

Wednesday, March 13, 2019 08:45:45

311LT-3 - Page 11 of 13

**RESIDUE #9 = G**

## Figure S5. B) continued

SequencePro™

Tufts Core Facility
Tufts Medical School
136 Harrison Ave
Boston, MA 02111

Cycle 12:Residue 10

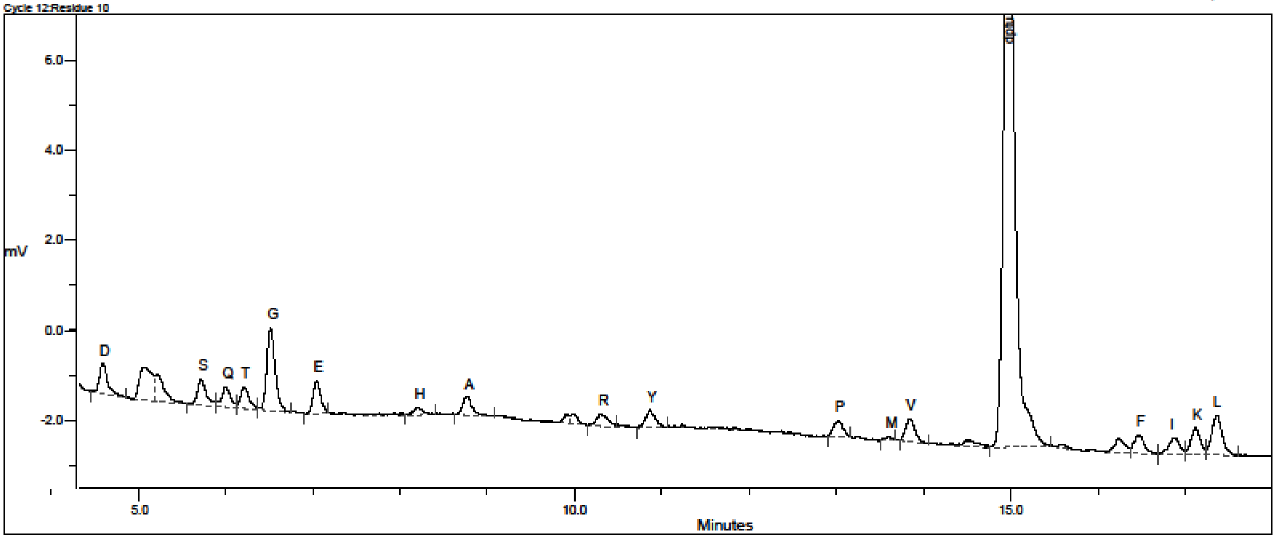


| PEAK ID | R.TIME (mins) | C.TIME (mins) | HEIGHT (mV) | PMOL HT |
|---|---|---|---|---|
| | 4.08 | | 13.828 | |
| D | 4.59 | 4.65 | 0.673 | 0.927 |
| | 5.08 | | 0.732 | |
| | 5.22 | | 0.607 | |
| S | 5.72 | 5.77 | 0.581 | 1.235 |
| Q | 6.00 | 6.06 | 0.462 | 0.787 |
| T | 6.21 | 6.26 | 0.489 | 1.314 |
| G | 6.51 | 6.57 | 1.850 | 3.460 |
| E | 7.05 | 7.11 | 0.742 | 1.094 |
| | 7.65 | | 0.036 | |
| | 7.76 | | 0.047 | |
| | 8.03 | | 0.048 | |
| H | 8.20 | 8.27 | 0.167 | 0.334 |
| | 8.42 | | 0.038 | |
| A | 8.77 | 8.84 | 0.403 | 0.712 |
| | 9.50 | | 0.039 | |
| | 9.90 | | 0.183 | |
| | 9.98 | | 0.207 | |
| R | 10.31 | 10.40 | 0.266 | 0.822 |
| | 10.56 | | 0.040 | |
| | 10.70 | | 0.029 | |
| Y | 10.87 | 10.96 | 0.392 | 0.751 |
| | 11.15 | | 0.026 | |
| | 11.23 | | 0.064 | |

| PEAK ID | R.TIME (mins) | C.TIME (mins) | HEIGHT (mV) | PMOL HT |
|---|---|---|---|---|
| | 11.45 | | 0.026 | |
| | 11.62 | | 0.039 | |
| | 11.83 | | 0.061 | |
| | 12.86 | | 0.039 | |
| P | 13.03 | 13.11 | 0.373 | 0.820 |
| M | 13.60 | 13.70 | 0.083 | 0.158 |
| V | 13.84 | 13.95 | 0.503 | 0.924 |
| | 14.33 | | 0.039 | |
| | 14.53 | | 0.153 | |
| | 14.74 | | 0.057 | |
| dptu | 14.98 | 15.07 | 15.201 | 34.132 |
| | 15.57 | | 0.079 | |
| | 16.25 | | 0.313 | |
| F | 16.48 | 16.57 | 0.405 | 0.773 |
| I | 16.86 | 16.98 | 0.375 | 0.802 |
| K | 17.12 | 17.22 | 0.605 | 0.972 |
| L | 17.36 | 17.46 | 0.883 | 1.720 |
| | 17.68 | | 0.038 | |
| | 18.07 | | 0.036 | |
| | 18.22 | | 0.033 | |
| | 18.53 | | 0.046 | |
| | 18.60 | | 0.030 | |
| | 18.73 | | 0.032 | |
| | 18.90 | | 0.035 | |

Wednesday, March 13, 2019 08:45:45

311LT-3 - Page 12 of 13

## RESIDUE #10 = -

## 10 cycles total

SequencePro™

Tufts Core Facility
Tufts Medical School
136 Harrison Ave
Boston, MA 02111

Raw Pmol Table

| | ASP | ASN | SER | GLN | THR | GLY | GLU | HIS | ALA | ARG | TYR | PRO | MET | VAL | TRP | PHE | ILE | LYS | LEU |
|---|---|---|---|---|---|---|---|---|---|---|---|---|---|---|---|---|---|---|---|
| 1 | 0.917 | 0.292 | 1.472 | 0.383 | 3.034 | 3.531 | 0.694 | 0.400 | 0.739 | 0.874 | 0.637 | 0.500 | 0.322 | 0.579 | 0.185 | 0.452 | 0.470 | 0.934 | 0.645 |
| 2 | 0.635 | 0.386 | 1.112 | 0.548 | 0.994 | 4.407 | 0.679 | 0.370 | 0.604 | 0.978 | 0.720 | 0.547 | 0.170 | 0.610 | 0.000 | 0.536 | 0.595 | 0.449 | 0.775 |
| 3 | 0.564 | 0.000 | 0.939 | 0.670 | 0.769 | 3.239 | 0.721 | 0.268 | 0.543 | 1.392 | 0.613 | 0.618 | 0.144 | 0.644 | 0.045 | 0.486 | 0.573 | 0.551 | 0.857 |
| 4 | 0.608 | 0.872 | 0.945 | 0.615 | 0.764 | 3.073 | 0.774 | 0.358 | 0.516 | 1.105 | 0.590 | 1.930 | 0.210 | 0.794 | 0.000 | 0.538 | 0.651 | 0.639 | 0.930 |
| 5 | 0.618 | 1.198 | 0.951 | 0.657 | 0.710 | 3.623 | 0.822 | 0.210 | 0.621 | 0.883 | 0.630 | 1.177 | 0.144 | 0.718 | 0.000 | 0.546 | 0.609 | 0.674 | 1.056 |
| 6 | 0.708 | 0.631 | 0.917 | 0.678 | 0.760 | 3.307 | 0.891 | 0.256 | 0.623 | 0.871 | 0.657 | 1.085 | 0.000 | 0.745 | 0.034 | 0.538 | 0.651 | 0.718 | 1.166 |
| 7 | 0.744 | 1.183 | 0.964 | 0.674 | 0.804 | 3.064 | 0.967 | 0.269 | 0.711 | 0.833 | 0.710 | 0.915 | 0.189 | 0.843 | 0.039 | 0.593 | 0.659 | 0.757 | 2.069 |
| 8 | 0.781 | 0.000 | 0.896 | 0.619 | 1.386 | 3.098 | 1.012 | 0.302 | 0.753 | 0.799 | 0.638 | 0.858 | 0.207 | 0.882 | 0.000 | 0.672 | 0.757 | 0.885 | 1.952 |
| 9 | 0.919 | 0.000 | 1.077 | 0.766 | 1.358 | 3.524 | 1.057 | 0.223 | 0.749 | 0.875 | 0.739 | 0.859 | 0.199 | 0.989 | 0.000 | 0.741 | 0.740 | 0.886 | 1.775 |
| 10 | 0.927 | 0.000 | 1.235 | 0.787 | 1.314 | 3.460 | 1.094 | 0.334 | 0.712 | 0.822 | 0.751 | 0.820 | 0.158 | 0.924 | 0.000 | 0.773 | 0.802 | 0.972 | 1.720 |

**Figure S6.** Original scans of immunoblots from Figures 1D, 2, and 4B showing MW ladder.

**Original scanned immunoblot for Figure 1D with ladder.** Note that MW marks were unfortunately not preserved for the actin blot. The location of the actin band relative to core and NS5A was consistent between this and other immunoblots in which the MW markers were recorded. We have therefore indicated the approximate location of the 46 kDa marker relative to actin on comparable immunoblots and indicated this with an "*" mark.

**Figure 1D**

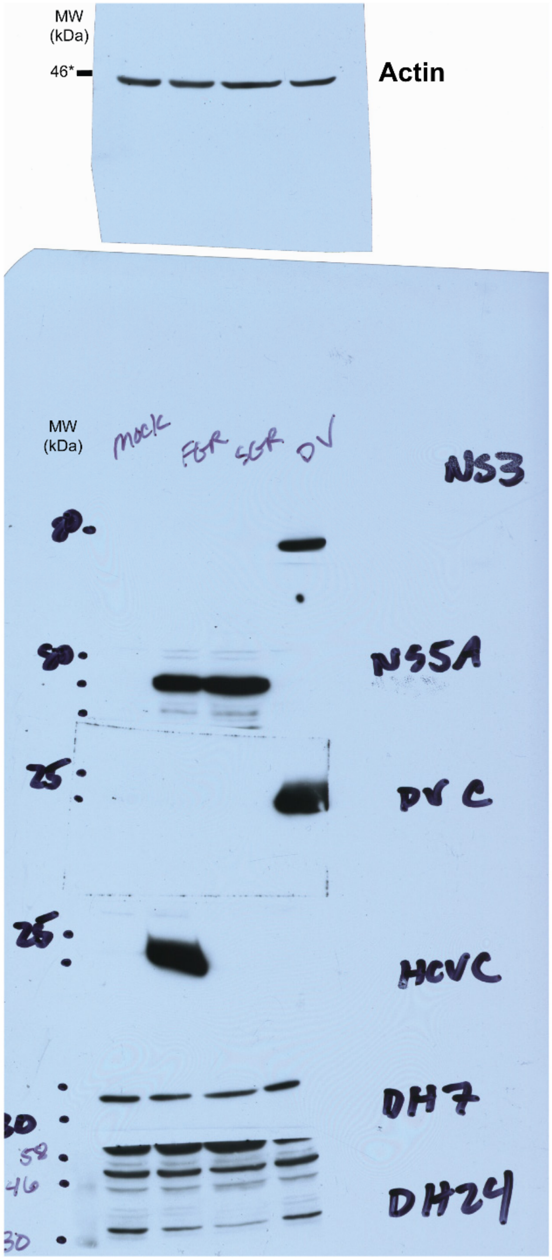

**Original scanned immunoblots for Figure 2 with respective MW ladders.** The areas highlighted by boxes in Figure 2C were discussed in the main study. While there is no marker indicated in the image presented in Figure 2E (excised during processing), the relative locations of the upper and lower bands are consistent with DHCR24 (higher) and DHCR24* (lower) observed in other experiments.

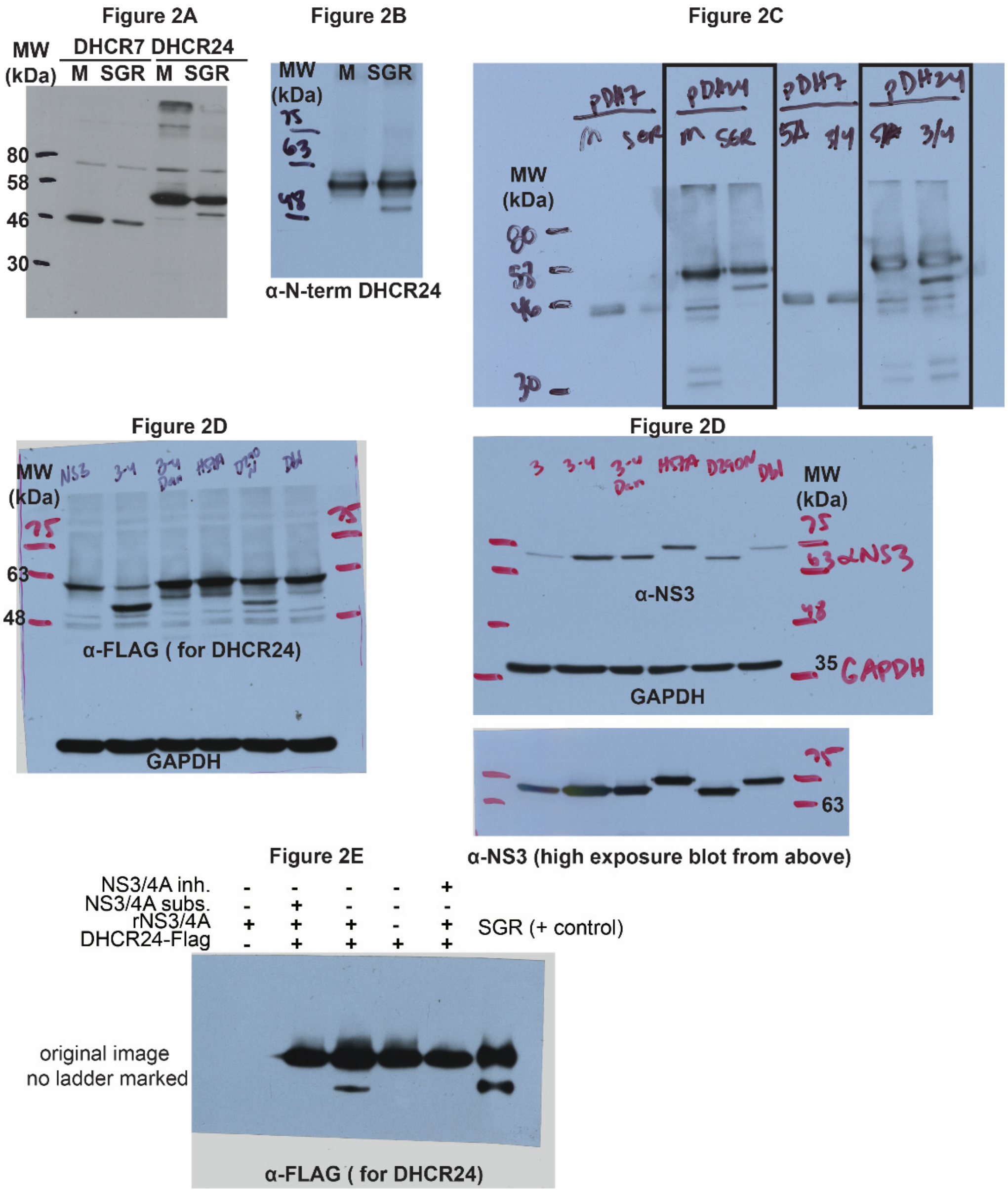

**Original scanned immunoblots for Figure 4B with respective MW ladders.**

Figure 4B

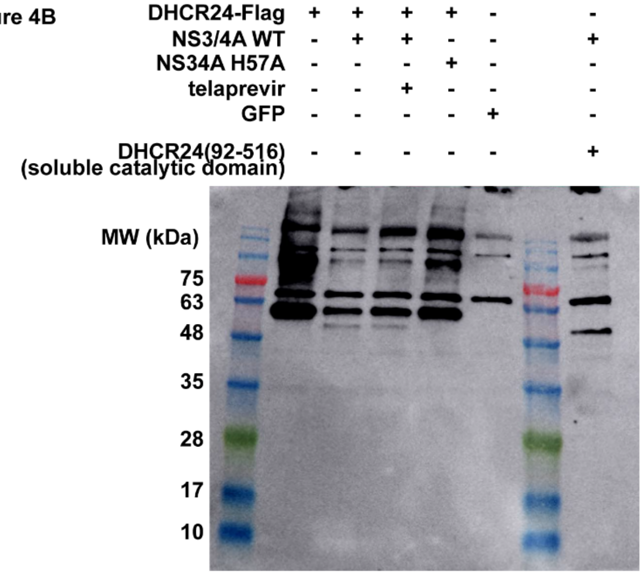


α-Flag

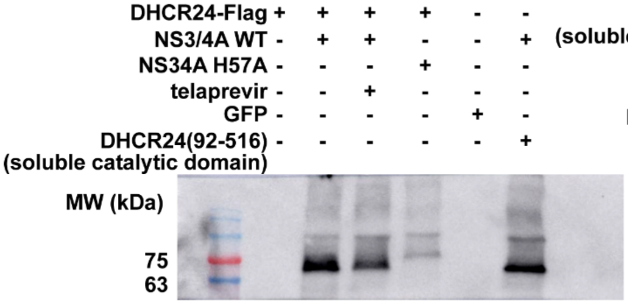


α-NS3

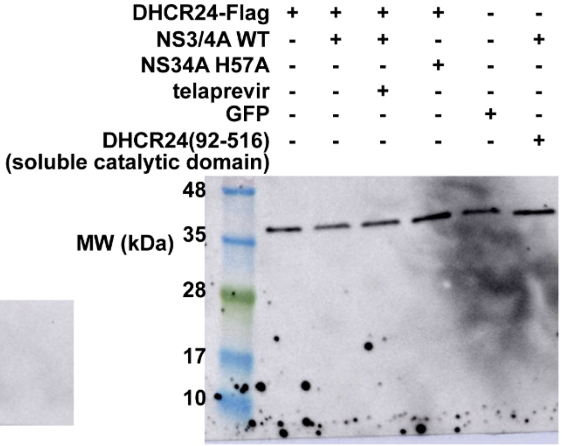


α-GAPDH

## DETAILED EXPERIMENTAL PROCEDURES

### A. Cells

**A1. Huh7.5 cells wild-type.** Huh7.5 cells were obtained from Charles Rice (Rockefeller University) and cultured in Dulbecco's Modified Eagle's medium (DMEM) supplemented with nonessential amino acids and 10% fetal bovine serum (FBS, Invitrogen) at 37°C incubator with 5% $CO_2$ (53).

**A2. Huh7.5-SGR cells-JFH1 (2a)*.*** Huh7.5-SGR cells stably replicating the HCV JFH1 (2a) subgenomic replicon Huh7.5 cells were electroporated with 7.5 µg of JFH1 subgenomic replicon *in vitro* transcript as previously described [Kato 2003]. Twenty-four hours after electroporation, cells were maintained in Huh7.5 cell in DMEM supplemented with nonessential amino acids, 10% FBS (FBS, Invitrogen) supplemented with 750 ug/mL G418 sulfate (18).

**A3. Generation of *DHCR24$^{KO}$* Huh7.5 cells.** *DHCR24$^{KO}$* Huh7.5 cells were generated by CRISPR/Cas9-mediated genome editing as previously described (55). DHCR24 has two isoforms that largely differ in the *N*-terminal region encoded in Exon 1. For this reason, Exon 2 was chosen as the target sequence. The removal introduced a frame shift resulting in a non-functional gene and partial removal of the *N*-terminal DHCR24 antibody epitope (DHCR24 residues 68-84). Two guide RNAs targeting removal of the splicing junction between intron1/exon2 encoded in exon 2 of DHCR24 (gRNA1: 5'-GCTTGTGGTGTAGCAATATGT-3' and gRNA2: 5'-GCTCACTGTCTCACTACGTGTG-3') were employed.

The CRISPR-DHCR24 double gRNA construct was transfected into Huh7.5 cells using Lipofectamine 2000 (Thermo Fisher Scientific) according to manufacturer's protocolin a T-75 flask seeded at a density of 2.1 x$10^6$ cells/mL following the manufacturer's protocol. The cells were cultured overnight at 37°C with 5% $CO_2$. Following transfection, the medium was replaced with medium containing a final concentration of 4 µg/mL puromycin (InvivoGen) to select for Huh7.5 cells containing transfected pCRISPR-DHCR24 2x-gRNA-*DHCR24$^{KO}$*. Cells viable after selection with puromycin over several days were subjected to monoclonal selection by dilution of the cells to 10 cells/10 mL resuspended in sterile-filtered 50% preconditioned media for monoclonal limiting dilution in a 96-well plate in the absence of puromycin. Medium in the wells was replaced every 7 days with non-conditioned media until the appearance of single colonies (~5 weeks) was observed. Upon observation of single colonies, wells containing single colonies were amplified and characterized by Sanger sequencing and immunoblot analysis for the elimination of the *N*-terminal DHCR24 epitope (Figure S3). Genome editing was confirmed by PCR. Diagnostic primers: Forward primer: 5'-AGA TGC CTA GTG TGT TGG GAA T-3' and Reverse primer: 5'-AAA GGG TTC CCC TGA CCT ATT A-3'.

**A4. *DHCR24$^{KO}$* Huh7.5 cells.** *DHCR24$^{KO}$* Huh7.5 cells were cultured in Dulbecco's Modified Eagle's medium (DMEM) supplemented with nonessential amino acids and 10% fetal bovine serum (FBS, Invitrogen) at 37°C incubator with 5% $CO_2$ without any supplementation of cholesterol.

**A5. VEEV-NS3-5B cells Huh7.5 cells.** VEEV-NS3-5B Huh7.5 cells were obtained from Brett Lindenbach (Yale University) and cultured in Dulbecco's Modified Eagle's medium (DMEM) supplemented with nonessential amino acids and 10% fetal bovine serum (FBS, Invitrogen) at 37°C with 5% $CO_2$ (36) with puromycin (5 µg/mL).This experimental model was utilized for immunofluorescence studies because it supports physiological expression of the nonstructural proteins in a system not dependent upon HCV RNA replication.

## B. Plasmids and cloning

**B1. *In cellulo* plasmids.** pCMV-DHCR24-MYC-DYKDDDDK and pCMV-DHCR7-MYC-DYKDDDDK were purchased from Origene. NS3, NS3-4A, NS4B, NS5A, and NS5B were amplified from pJFH1 and subcloned into pCMV-Entry6 (Origene) using standard restriction cloning. Site-directed mutagenesis was applied to introduce a stop codon to eliminate inclusion of MYC- DYKDDDDK for viral proteins and to introduce mutations (H57A, D290A, and double mutant H57A/D290A) to NS3-4A by QuikChange II Site-Directed Mutagenesis (Agilent). pIRES_DHCR24-Flag constructs containing a hygromycin resistant cassette were made using NEBuilder and NEBuilder HiFi DNA Assembly Master Mix (NEB). A silent mutation at Arg103 was made to prevent any possible guide RNAs from recognizing newly transfected pIRES_DHCR24-Arg103-Flag constructs and mutants in *DHCR24*$^{KO}$ Huh7.5 cells. pIRES_DHCR24-Flag mutants (C91P, C91N, C91F, C91A-T92A, C91V-T92V, C91S, C91T, and ΔC91-92) were designed and generated using In-Fusion Cloning Primer Design Tool online software, CloneAmp HiFi PCR Premix for PCR amplification of the linearized template (pIRES_DHCR24-Arg103-Flag) (Takara), and In-Fusion HD Cloning Plus (Takara).

**B2. *In vitro* plasmids.** pMALc5x_maltose binding protein (MBP)-DHCR24 and pMALc5x-DHCR24 (full-length)-super folded green fluorescent protein (sfGFP) containing an ampicillin resistant cassette were purchased from GenScript USA Inc. The super-folded (sf)GFP cassettes were based on constructs that have previously been expressed and described (56). The various surrogate DHCR24 peptide constructs spanning residues 56-110, 52-66, and 85-99, and the 85-99-C91P mutant were expressed as fusions between an *N*-terminal MBP and sfGFP with a *C*-terminal DYKDDDDK (Flag-Tag). Expressions constructs were generated by overlapping extension PCR using Q5 High-Fidelity DNA Polymerase (NEB) and were subcloned into a pET28b plasmid containing a kanamycin resistance cassette. These plasmids were transformed into Rosetta2 (DE3) pLysS host strain (Novagen) following the manufacturer's protocol.

## C. Immunoprecipitation

**C1. Preparation of samples for immunoprecipitation of DHCR7-Flag and DHCR24-Flag.** Transfection of Huh7.5 cells, or Huh7.5 cells stably harboring SGR or SGR-GFP under G418 selection (750 µg/mL), were transfected with plasmids using Lipofectamine 2000 (Thermo Fisher Scientific) within 30 minutes of seeding. For a T75 flask, Huh7.5 cells were seeded at 60% confluency and transfected with 20 µg of either DHCR7-Flag or DHCR24-Flag, 20 µL of Lipofectamine 2000 (Thermo Fisher Scientific), and one mL of Opti-MEM (Gibco) with incubation times followed according to manufacturer's protocol. Sixteen hours later, the cell culture medium was removed and replaced with fresh medium. Forty eight hours after the addition of transfection reagents, the cells were lysed with RIPA buffer (Boston BioProducts) and HALT protease inhibitor cocktail (Roche) and stored at -20ºC until analysis.

**C2. Immuno precipitation and immunoblot analysis of immunoprecipitates.** Samples for immunoprecipitation were thawed on ice and clarified by centrifugation (18,000 x *g*) for five minutes. Protein lysate was collected by vigorously pipeting cell pellets in RIPA buffer supplemented with Halt protease cocktail inhibitor (Roche), and protein content was quantified by Bradford assay (Pierce). 50 µg of protein (each of two biological replicate) was applied to the

magnetic anti-DYKDDDDK resin (Clontech), which was incubated with supernatant overnight at 4ºC with a tube rotisserie. After incubation, the resin was separated magnetically and washed six times with RIPA buffer. Sample was boiled and eluted with 1X SDS denaturing loading dye to elute immunoprecipitated protein and subjected to separation by SDS-PAGE followed by transfer to a PVDF membrane (semidry transfer at 10 volts for 1.5 hours). The membrane was washed with TBST followed by blocking in 5% milk (TBST, w/v). Post-blocking, the membranes were incubated with their respective primary antibodies overnight at 4°C rocking in the dark. The membranes were then washed with TBST, followed by incubation with a species-specific secondary antibody. After washing with TBST, the membrane was incubated with a chemiluminescent reagent (Pierce) and exposed to an X-ray film for imaging.

Samples containing DHCR24-MYC-DYKDDDDK and DHCR7-MYC-DYKDDDDK were detected with anti-FLAG-horseradish peroxidase M2 (Sigma). Additional antibodies were purchased or acquired for immunoblot analysis: Anti-NS3 (1:1000) (Abcam), anti-NS5A (1:1000) (9E10 from C. Rice), anti-GAPDH (1:25,000) (Genetex GTX28245), and anti-DHCR24 (1:500) (Sigma S8571). Each respective blot was incubated with their respective species-specific secondary horseradish peroxidase (HRP)-conjugated goat anti-mouse IgG (BioRad 170-6516) and anti-rabbit IgG (BioRad 170-6515) for 1 hour at room temperature.

**C3. Co-transfection of viral genes and DHCR24-Flag.** 3 x $10^5$ Huh7.5 cells were seeded per well in a 6-well plate. Cells were co-transfected with 1 µg pCMV-NS3-4A (variants or NS3) + 2.6 µg pCMV-DHCR24-FLAG (WT) + 4 µL Lipofectamine and 250 µL Opti-Mem. Cells were allowed to recover at 37 °C for 20 min prior to addition of Lipo-DNA mix. Danoprevir, an NS3-4A inhibitor, (10 µM) was immediately added after delivery of Lipo-DNA mix. After 48 hours post-transfection, cells were lysed with RIPA buffer and frozen at 20°C. Thawed, clarified lysates were analyzed with anti-DHCR24, NS3 or GAPDH antibody. Immunoblot analysis is repeated as described in *Immunoblot analysis of immunoprecipitates.*

**C4. Co-transfection studies of DHCR24-Flag with variants of NS3-4A.** Analysis of ectopically expressed DHCR24-Flag: 90% confluent Huh7.5 cells or Huh7.5/SGR cells (750 µg/mL G418) were plated in a T75 flask (1:2 split) and allowed to recover in complete medium for 25 minutes prior to addition of Lipofectamine-DNA complexes containing Lipofectamine 2000 + DNA: 20 µL Lipofectamine + 20 µg pCMV-DHCR24-MYC- DYKDDDDK. For experiments involving NS5A and NS3: 40 µL Lipofectamine + 20 µg pCMV DHCR24-MYC- DYKDDDDK + 20 µg pCMV-NS5A or pCMV-NS3-4A. Danoprevir (10 µM) was immediately added after delivery of the Lipofectamine-DNA mix. After 18 hours, the medium was changed. Cells were then harvested 68 hours post-transfection with non-denaturing lysis buffer (10 mM phosphate, 150 mM NaCl, 1% NP40 pH 7.5). DHCR24-Flag was immunoprecipitated as previously described. Analysis of endogenous DHCR24: T150 flasks were seeded with Huh7.5/SGR (under 750 µg/mL G418 selection) or Huh7.5 cells. 72 hours later, cells were harvested with RIPA non-denaturing lysis buffer. ~1 mL supernatant was incubated with 2 µL of anti-DHCR24 antibody (Sigma S8571) overnight at 4° C. 15 hours later, 40 µL of goat anti-rabbit resin was added and incubated for four hours. The resin was washed with non-denaturing lysis buffer using gravity flow. Samples were eluted with 1X SDS denaturing loading dye. Thawed, clarified samples were analyzed by immunoblot using anti-DHCR24, NS3 or GAPDH antibodies.

**D. Protein expression**

**D1. Recombinant expression.** MBP-DHCR24-56-110-GFP, MBP-DHCR24-52-66-GFP, MBP-DHCR24-85-99, and MBP-DHCR24-85-99-C91P-GFP all have a *C*-terminal DYKDDDDK (Flag) tag and were recombinantly expressed in the Rosetta2 (DE3) pLysS (Novagen) *E. coli* host strain. Cells were grown in terrific broth media containing chloramphenicol (25 µg/mL) and kanamycin sulfate (50 µg/mL). When the optical density reached 0.8-1.0 at 600 nm, cells were induced by the addition of isopropyl-β-D-thiogalactopyranoside (IPTG, 500 µM). After growing overnight at 16°C, the cells were harvested by centrifugation, resuspended in lysis buffer (50 mM HEPES, pH 7.8, 100 mM NaCl, 10% glycerol, 5 mM DTT), and supplemented with 0.1 mg/ mL lysozyme (Sigma) and 5 µg/ mL DNAse I (Sigma). The cell lysate was passed through an EmulsiFlex-C5 disrupter (Avestin) at 15,000 PSI three times and centrifuged for 30 minutes (15,000 x *g*, 4°C) to remove cell debris. A three-step purification process was followed to obtain purified test substrates. This included an initial purification on amylose resin (NEB) following the manufacturer's protocol, followed by size-exclusion chromatography on a Size Superdex 200 Increase 10/300 GL column (GE Healthcare), using degassed and 0.2 µm sterile filtered (Corning) SEC buffer (50 mM HEPES, pH 7.8, 100 mM NaCl, 10% glycerol, 5 mM DTT) and a protocol from the UNICORN6 software package (GE Healthcare) specific to the Superdex column.  was used . Finally, the protein (~71.6 KDa) was concentrated in a 50 kDa Amicon ultra centrifugation filter (MilliporeSigma) and purified using a magnetic anti-Flag affinity resin (Pierce) following the manufacturer's protocol. The eluted sample was immediately buffer exchanged into SEC buffer using a 7K MWCO Zebra spin desalting column (Thermo Scientific) to eliminate any excess FLAG peptide following the manufacturer's protocol. The purified proteins were flash frozen in liquid nitrogen and stored at -80 °C.

**D2. *In vitro* protein synthesis.** MBP-DHCR24 (full-length) in a pET28b vector (Novagen) was expressed using the PURExpress *in vitro* synthesis kit (NEB E6800S) as previously described (Shimazu 2001). Briefly, the pET28_MBP-DHCR24 plasmid was prepared using a maxi-prep kit (Qiagen), eluted in ultra-pure water (Invitrogen), and subsequently used in the *in vitro* translation reaction. To the *in vitro* translation reaction mixture, RNAsin (Promega) and 450 ng of purified plasmid were added and incubated for 4 hours (37 °C, 250 RPM). The reaction mixture was concentrated using a 0.5 mL Ultra Centrifugal Filter MWCO of 50 kDa to eliminate transcription and translation proteins from the kit. The protein mixture was flash frozen in liquid nitrogen and stored at -80°C.

**E. Biochemical assays**

**E1. *In vitro* experiments with recombinant NS3-4A.** The various DHCR24 fusion constructs either purified from recombinant expression in *E.coli* or through *in vitro* translation were incubated with recombinant NS3-4A (AnaSpec) for 16 hours (30 °C, 250 RPM) containing: 50 µM DHCR24 test substrate and 18 µM NS3-4A in 50 mM HEPES (pH 7.5), 100 mM NaCl, 20% glycerol, 1 µM DTT. The reaction was quenched by the direct addition of 1X SDS denaturing loading dye.

**E2. Immunoblot analysis to of NS3-4A cleavage assays.** 100 µg of the reaction mixture (25 µL) was analyzed by SDS-PAGE using a 8-16% TGX Mini-protean gel (BioRad). The gel was transferred onto nitrocellulose membrane (BioRad Trans-blot Turbo). The membrane was

washed in TBST and blocked with 5% milk (TBST, w/v). After incubation overnight with primary antibodies at 4 °C, membranes were washed with TBST and incubated with their respective species-specific secondary antibodies. After washing with TBST, blots were developed with enhanced chemiluminescence reagents (Thermo Fisher Scientific), and the chemiluminescence signal was processed using the Amersham Imager 600 (GE Healthcare).

Primary antibodies and their dilutions were as follows: anti-DHCR24 (rabbit) (epitope region 68-85) (1:2000), anti-MBP-horseradish peroxidase (NEB E8038) (1:2000), and anti-FLAG-horseradish peroxidase M2 (Sigma A8592) (1:1000). Anti-rabbit IgG-HRP (BioRad 170-6515) was used to blot against the anti-DHCR24 (epitope region 68-85) (Sigma S8571) (1:2000).

**F. Lipid analyses**

**F1. Lipid isolation.** Cells were washed once with 1X PBS and harvested by trypsinization, addition of medium to quench trypsin, and centrifugation (1000 x *g*, 5 minutes, 4 °C). Cell pellets were washed twice with cold 1X PBS, resuspended in 1 mL of PBS and processed with a Dounce homogenizer using 2:1:1 $CHCl_3$:$CH_3OH$:PBS buffer. The organic and aqueous layers were separated by centrifugation (2000 x *g*, 5 minutes, 4°C). The organic layer was removed, concentrated under nitrogen gas ($N_2$), and then dissolved in 90 µL $CHCl_3$ prior to analysis by LC-MS or GC-MS. 30 µL of this sample was used for LC-MS. Absolute quantitation of sterol levels was accomplished by isotope dilution mass spectrometry and adding deuterated ketocholesterol-d7 (5 µL of 10 µM stock) as an internal quantitation standard. For GC-MS samples, samples were dried down with the internal standard (ketocholesterol-d7).

**F2. Liquid Chromatography- Mass Spectrometry.** LC-MS was performed on an Agilent 6530 QTOF mass spectrometer with 1290 Infinity Binary LC in positive ion mode. For the quantification of desmosterol, lipids were chromatographically separated on a UHPLC Phenomenex Kinetex 2.6 µM XBC18, 100 Å column. Gradients using mobile phase A (85% methanol, 15% water, 5 mM ammonium acetate, and 0.1% formic acid) and mobile phase B (100% methanol, 5 mM ammonium acetate, and 0.1% formic acid) were run as follows: 0−2 min 100% A, 2−15 min 100% B, 15−25.5 100% B, and 25.5−30 min 100% A.

**F3. Gas Chromatography- Mass Spectrometry.** GC/MS analysis was performed on a Thermo Scientific TRACE 1310 Gas Chromatograph equipped with a Thermo Scientific Q Exactive Orbitrap mass spectrometry system. 50 µL of the (BSTFA+10% TMCS)/pyridine (1/1 v/v) derivatized product was added into each vial, vortexed well, and heated at 70˚C for 30 min. One µL of the sample was injected into a Thermo fused-silica capillary column of cross-linked TG-5SILMS (30 m x 0.25 mm x 0.25 µm). The GC conditions were as follows: inlet and transfer line temperatures, 290˚C; oven temperature program, 50˚C for 0 min, 24˚C/min to 325˚C for 5.7 min; inlet helium carrier gas flow rate, 1 mL/min; split ratio, 5. The electron impact (EI)-MS conditions were as follows: ion source temperature, 310˚C; full scan m/z range, 30 - 750 Da; resolution, 60,000; AGC target, 1e6; maximum IT, 200ms. Data were acquired and analyzed with Thermo TraceFinder 4.1 software package.

**G. Mass Spectrometry Analysis of Protein Bands** Excised gel bands were cut into approximately 1 $mm^3$ pieces and submitted to the Taplin Mass Spectrometry Facility for subsequent processing and analysis. The samples were reduced with 1 mM DTT for 30 minutes

at 60ºC and then alkylated with 5 mM iodoacetamide for 15 minutes in the dark at room temperature. Gel pieces were then subjected to a modified in-gel trypsin digestion procedure (57). Gel pieces were washed and dehydrated with acetonitrile for 10 min, followed by removal of acetonitrile. Pieces were then completely dried in a Speed-Vac. Rehydration of the gel pieces was with 50 mM ammonium bicarbonate solution containing 12.5 ng/µl modified sequencing-grade trypsin (Promega, Madison, WI) at 4ºC. Samples were then placed in a 37 ºC room overnight. Peptides were later extracted by removing the ammonium bicarbonate solution, followed by one wash with a solution containing 50% acetonitrile and 1% formic acid. The extracts were then dried in a speed-vac (~1 hr). The samples were then stored at 4 ºC until analysis.

On the day of analysis the samples were reconstituted in 5 - 10 µL of HPLC solvent A (2.5% acetonitrile, 0.1% formic acid). A nano-scale reverse-phase HPLC capillary column was created by packing 2.6 µm C18 spherical silica beads into a fused silica capillary (100 µm inner diameter x ~30 cm length) with a flame-drawn tip (58). After equilibrating the column each sample was loaded via a Famos auto sampler (LC Packings, San Francisco CA) onto the column. A gradient was formed and peptides were eluted with increasing concentrations of solvent B (97.5% acetonitrile, 0.1% formic acid). As each peptide was eluted, they were subjected to electrospray ionization followed by entry into an LTQ Orbitrap Velos Pro ion-trap mass spectrometer (Thermo Fisher Scientific, San Jose, CA). Eluting peptides were detected, isolated, and fragmented to produce a tandem mass spectrum of specific fragment ions for each peptide. Peptide sequences (and hence protein identity) were determined by matching protein or translated nucleotide databases with the acquired fragmentation pattern by the software program, Sequest (ThermoFinnigan, San Jose, CA) (59). The modification of 79.9663 mass units to serine, threonine, and tyrosine was included in the database searches to determine phosphopeptides. Phosphorylation assignments were determined by the Ascore algorithm (60). All databases include a reversed version of all the sequences and the data was filtered to between a 1-2% peptide false discovery rate.

### H. Immunofluorescence Microscopy

VEEV-NS3-5B cells were seeded at a density of 0.5 x $10^5$ cells/mL in µ-Slide 8 Well Glass Bottom: # 1.5H (170 µm +/- 5 µm) D 263 M Schott glass, chambered slides (Ibidi) and incubated for 48 hours under 5 µg/mL puromycin selection (5% $CO_2$, 37 °C). At approximately 70% cell confluency, the medium was replaced with pre-warmed DMEM without phenol red for 1 hour (5% $CO_2$, 37 °C). Cells were washed twice with filtered PBS and fixed with cold methanol for 1-2 hours at 4 °C, then were washed twice with PBS and permeabilized with 0.1% Triton-X 100 (15 minutes, room temperature). Cells were then washed with PBS and blocked in blocking solution (10% fetal bovine serum and 5% bovine serum albumin in PBS) (1 hour, 37°C). Cell were then sequentially stained for DHCR24 or PDI followed by staining for NS3. First, cells were incubated with anti-DHCR24 or anti-PDI primary antibodies in blocking buffer (overnight, 4 °C). Cells were washed in PBS and incubated with the appropriate secondary antibodies in a humidity chamber (1 hour, at 37 °C dark). After this first staining, cells were washed with PBS and stained with anti-NS3 in blocking buffer (1 hour, 37 °C, dark) followed by secondary antibody staining as described. Samples were mounted with ProLong Gold antifade reagent (Invitrogen P36930).

Primary and secondary antibody combinations and their dilutions were as follows: anti-DHCR24 (epitope region 68-85) (Sigma S8571) (1:1500) used with AlexaFluor647-anti-rabbit (Invitrogen A21244) (1:1000), anti-PDI (Abcam ab110195) (1:500) used with AlexaFluor555 anti-

goat (Invitrogen A32816) (1:1000), and anti-NS3 (Abcam ab65407) (1:500) used with AlexaFluor488 anti-mouse (Invitrogen A11001) (1:1000).

Images were acquired with a Nikon Ti-E inverted microscope system using a Plan Apo 100x/1.45 objective with a Hamamatsu Orca Flash sCMOS camera, SOLA light engine (Lumencor), Shutter and LUDL motorized excitation/emission filter wheels on a Nikon Ti-E inverted microscope equipped with a Yokogawa CSU-W1 spinning disk scanhead using laser lines 488, 514, 561 and 640 (Toptica) and a Plan Apo 100x/1.45 objective DIC. Images were acquired with the NIS-Elements software package.

## References for Supporting Information

18. Kato, T., Date, T., Miyamoto, M., Furusaka, A., Tokushige, K., Mizokami, M., and Wakita, T. (2003) Efficient replication of the genotype 2a hepatitis C virus subgenomic replicon. *Gastroenterology*. **125**, 1808–1817

36. Kazakov, T., Yang, F., Ramanathan, H. N., Kohlway, A., Diamond, M. S., and Lindenbach, B. D. (2015) Hepatitis C Virus RNA Replication Depends on Specific Cis- and Trans-Acting Activities of Viral Nonstructural Proteins. *PLOS Pathogens*. **11**, e1004817

53. Lindenbach, B. D., Evans, M. J., Syder, A. J., Wölk, B., Tellinghuisen, T. L., Liu, C. C., Maruyama, T., Hynes, R. O., Burton, D. R., McKeating, J. A., and Rice, C. M. (2005) Complete Replication of Hepatitis C Virus in Cell Culture. *Science*. **309**, 623–626

55*. Langereis, M. A., Rabouw, H. H., Holwerda, M., Visser, L. J., and Kuppeveld, F. J. M. van (2015) Knockout of cGAS and STING Rescues Virus Infection of Plasmid DNA-Transfected Cells. *Journal of Virology*. **89**, 11169–11173

56*. Pédelacq, J.-D., Cabantous, S., Tran, T., Terwilliger, T. C., and Waldo, G. S. (2006) Engineering and characterization of a superfolder green fluorescent protein. *Nature Biotechnology*. **24**, 79–88

57*. Shevchenko, A., Wilm, M., Vorm, O., and Mann, M. (1996) Mass spectrometric sequencing of proteins silver-stained polyacrylamide gels. *Anal. Chem.* **68**, 850–858

58*. Peng, J., and Gygi, S. P. (2001) Proteomics: the move to mixtures. *J Mass Spectrom*. **36**, 1083–1091

59*. Eng, J. K., McCormack, A. L., and Yates, J. R. (1994) An approach to correlate tandem mass spectral data of peptides with amino acid sequences in a protein database. *J. Am. Soc. Mass Spectrom.* **5**, 976–989

60*. Beausoleil, S. A., Villén, J., Gerber, S. A., Rush, J., and Gygi, S. P. (2006) A probability-based approach for high-throughput protein phosphorylation analysis and site localization. *Nat. Biotechnol.* **24**, 1285–1292

* = Denotes additional reference number after reference list located in the main text.
